# Integrated Proteomic and Metabolic Analysis of Breast Cancer Progression

**DOI:** 10.1371/journal.pone.0076220

**Published:** 2013-09-27

**Authors:** Patrick G. Shaw, Raghothama Chaerkady, Tao Wang, Shauna Vasilatos, Yi Huang, Bennett Van Houten, Akhilesh Pandey, Nancy E. Davidson

**Affiliations:** 1 University of Pittsburgh Cancer Institute, Pittsburgh, Pennsylvania, United States of America; 2 Department of Medicine, University of Pittsburgh, Pittsburgh, Pennsylvania, United States of America; 3 Department of Pharmacology and Chemical Biology, University of Pittsburgh, Pittsburgh, Pennsylvania, United States of America; 4 Department of Biochemistry and Molecular Biology, Johns Hopkins University Bloomberg School of Public Health, Baltimore, Maryland, United States of America; 5 McKusick-Nathans Institute of Genetic Medicine and Departments of Biological Chemistry, Johns Hopkins University School of Medicine, Baltimore, Maryland, United States of America; University of Alabama at Birmingham, United States of America

## Abstract

One of the most persistent hallmarks of cancer biology is the preference of tumor cells to derive energy through glycolysis as opposed to the more efficient process of oxidative phosphorylation (OXPHOS). However, little is known about the molecular cascades by which oncogenic pathways bring about this metabolic switch. We carried out a quantitative proteomic and metabolic analysis of the MCF10A derived cell line model of breast cancer progression that includes parental cells and derivatives representing three different tumor grades of Ras-driven cancer with a common genetic background. A SILAC (Stable Isotope Labeling by Amino acids in Cell culture) labeling strategy was used to quantify protein expression in conjunction with subcellular fractionation to measure dynamic subcellular localization in the nucleus, cytosol and mitochondria. Protein expression and localization across cell lines were compared to cellular metabolic rates as a measure of oxidative phosphorylation (OXPHOS), glycolysis and cellular ATP. Investigation of the metabolic capacity of the four cell lines revealed that cellular OXPHOS decreased with breast cancer progression independently of mitochondrial copy number or electron transport chain protein expression. Furthermore, glycolytic lactate secretion did not increase in accordance with cancer progression and decreasing OXPHOS capacity. However, the relative expression and subcellular enrichment of enzymes critical to lactate and pyruvate metabolism supported the observed extracellular acidification profiles. This analysis of metabolic dysfunction in cancer progression integrated with global protein expression and subcellular localization is a novel and useful technique for determining organelle-specific roles of proteins in disease.

## Introduction

Breast cancer remains a leading cause of death among women in the U.S. and around the world. Therefore, it is important to understand the critical molecular steps in the development, proliferation and metastasis of breast cancer. Cell line models have been useful tools for understanding the progression of the disease. One well studied model was generated when an immortalized mammary epithelial cell line, MCF10A [1], was transfected with *HRAS* oncogene and implanted into mice to form xenograft tumors [2]. A cell line derived from these tumors, MCF10A-T1K, was found to form tumors in 25% of immunocompromised mice when implanted as xenografts, without significant morbidity. However, repeated passaging in mice as xenografts led to the selection of cell lines that produced tumors in 100% of implanted mice with the animals becoming moribund within weeks because of metastases [3]. This study makes use of the parental MCF10A line (10A), the pre-neoplastic MCF10A-T1K cells (T1K), the low-grade tumorigenic MCF10CA1h cells (CA1h) which result in animal mortality within six weeks, and the high-grade tumorigenic MCF10CA1a (CA1a) cells which kill animals within three weeks and form lung metastases upon tail-vein injection.

Multiple groups have utilized this MCF10A based progression model to study the progression of breast cancer over a common genetic background. For instance, karyotype abnormalities and gene copy number variations have been observed across the cells as a result of the genomic instability inevitably associated with cancer progression [4]. Other groups have described single nucleotide polymorphisms (SNPs) and alterations in global gene expression, which identified extensive copy number-independent variation in gene expression across the progression series [5-7]. Additionally, various combinations of the MCF10A-derived breast cancer progression cell lines have been profiled using proteomic techniques including immunological arrays [8] and mass spectrometry based methods [9-11]. Some proteomic studies have observed dynamic regulation of cellular metabolism pathways in these cell lines [9,11] suggesting that this model system could be used to study the relationship between oncogenesis and metabolism originally hypothesized by Warburg [12]. Also, recent studies have indicated that *HRAS* transfection alone can cause metabolic dysfunction [13] although the mechanism by which metabolism is altered over the course of cancer development is still unclear.

Here, we evaluate each of these four MCF10A-derived cell lines for basal OXPHOS and glycolysis and use a series of metabolic inhibitors to further probe the cellular metabolic capacity. In order to fully elucidate the molecular changes that coincide with or directly lead to metabolic dysfunction, we measured cellular protein expression as well as subcellular localization of proteins using a quantitative SILAC approach. Subcellular fractionation enabled the identification and quantification of both the cytoplasmic and mitochondrial isoforms of several major metabolic enzymes. Overall, our strategy revealed enrichment of some oncoproteins within subcellular fractions that may play a functional role in metabolic dysfunction.

## Materials and Methods

### Cell culture

Each of the MCF10A progression cell lines was passaged in DMEM/F12 medium (Thermo Scientific) as previously described [14]. SILAC experiments were carried out using Lysine-^13^C_4_ and Arginine-^13^C_6_ for ‘medium’ and Lysine-^13^C_6_-^15^N_2_ and Arginine-^13^C_6_-^15^N_4_ for ‘heavy’ labeling, respectively, as previously described [15,16]. Cells were grown in a humidified environment with 5% CO_2_ and changed to serum free minimal medium 4 hours prior to each experiment.

### Subcellular fractionation and sample preparation

**Figure 1:**
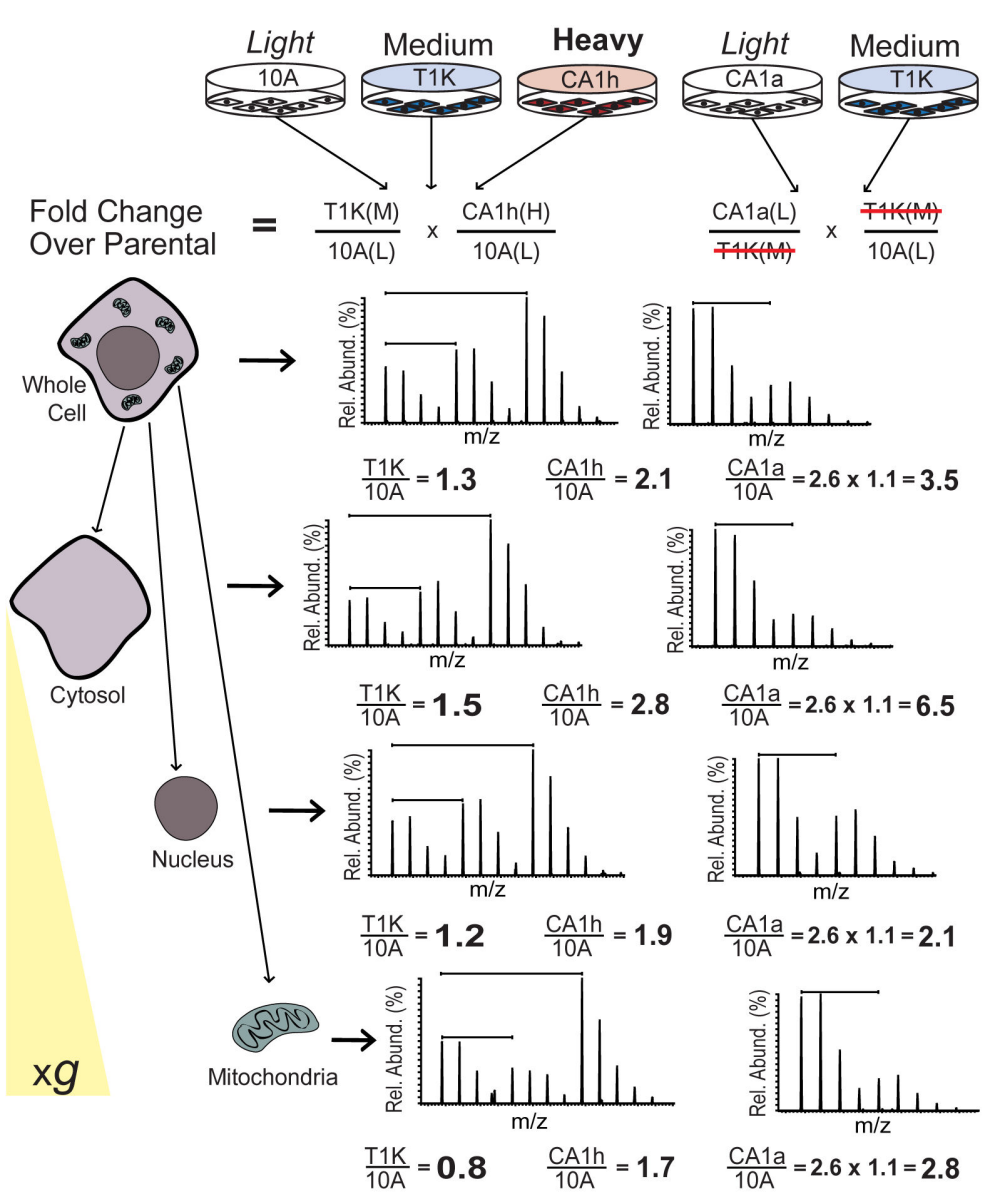
Summary of experimental workflow and data normalization. Lysates from 10A, T1K and CA1h cells were grown in light, medium or heavy SILAC medium, respectively, and equal numbers of cells were combined to obtain ratios of protein expression relative to parental cells. Light labeled CA1a cells were likewise combined with medium labeled T1K, and light to heavy ratios were normalized to the T1K fold change ratios in the 3-state experiment to calculate fold change of protein expression in CA1a cells relative to parental cells. After combining equal numbers of labeled cells, subcellular fractions of cytosolic, nuclear and mitochondrial proteins were obtained by centrifugation (500xg) over a sucrose gradient. Spectra from the peptide NPDDITQEEYGEFYK are shown to illustrate the fold change calculation for heat shock protein 90, which was identified in every cellular fraction and is disproportionately localized from the nucleus to the cytosol with breast cancer progression.

After dissociation by trypsinization (TrypLE Express, Gibco), cells were centrifuged at 500 xg for 5 minutes and pellets were washed twice by resuspending in 100x volume of Dulbecco’s PBS (Gibco). Cells were counted twice using a hemocytometer and confirmed to be greater than 95% viable by Trypan blue exclusion assay. Cells were assayed in triplicate for copy number analysis and an equal number of cells were combined for the respective SILAC experiments (Figure 1). Subcellular fractionation was performed as previously described [17] with some modifications. Briefly, a pellet of combined SILAC cells was set aside for whole cell protein analysis by centrifugation at 500 x*g* for 5 minutes. The remaining combined SILAC cells were lysed for subcellular fractionation by stroking in a Dounce homogenizer. Nuclei and mitochondria were extracted by iterative centrifugation steps as described [17]. The resulting supernatant containing cytosolic proteins along with Golgi, endoplasmic reticulum, vesicles and plasma membrane was retained as a single sample. Whole cell, nuclear and mitochondrial pellets were resuspended in modified hypertonic RIPA buffer (50mM Tris pH 7.4, 500mM NaCl, 0.25% Na deoxycholate, 1% NP-40, 0.5% SDS) and cytosolic fractions were adjusted to a final concentration equivalent to hypertonic RIPA. Cells and organelles were then homogenized by sonicating over ice with a Branson 250 Sonifier 3 times for 30 seconds (30% duty cycle, 3 output) and cleared by centrifugation at 16,100 x g for 10 minutes at 4°C. Protein concentration was determined by the Lowry method (Bio-Rad) and approximately 100 µg of each sample was boiled for 10 minutes in Laemmli buffer prior to separation by SDS-PAGE. Protein bands were visualized by colloidal Coomassie staining (Invitrogen) and each sample lane was divided into six regions of identical molecular weight range before in-gel tryptic digestion following a previously published protocol [15].

### LC-MS/MS

Peptides were resuspended in 0.1% formic acid and analyzed by direct online injection into an LTQ-Orbitrap XL ETD mass spectrometer (Thermo Scientific) using an Agilent 1100 autosampler coupled to an Eksigent nanopump. Chromatographic separation was performed over a gradient of 9-55% acetonitrile containing 0.1% formic acid over 55 minutes on a 50 µm x 15 cm analytical column packed in-house (Magic 5.0 µm, 300Å pore size, Michrom) with a flow rate of 300 nl/min. Peptides were sprayed using 15 µm emitter tip (PF3360-75-15-N-5, New Objective, www.newobjective.com) using spray voltage of 2.0 kV and capillary temperature of 200°C. Survey scans were acquired in the Orbitrap from a range of m/z 350-1800 at 60,000 resolution with poly dimethylcyclosiloxane from ambient air (m/z 445.120025) as a lock mass [18]. For each cycle, the 8 most intense ions were fragmented by collision induced dissociation (CID) in the ion-trap at a target of 20,000 ions or 100 ms maximum injection time before being excluded for 30 seconds. An exclusion list was generated for quantified spectra from whole cell lysate peptides by executing data analysis with Mascot (see below) and whole cell lysate peptides were analyzed a second time, excluding these masses for the full duration.

### Data analysis

SILAC ratios were quantified using Proteome Discoverer (Version 1.3) and spectra were searched against the human RefSeq 46 database with up to 2 missed cleavages, a precursor mass tolerance of 20 ppm and a fragment mass tolerance of 0.5 Dalton. Peptides and proteins were identified using the Mascot (v.2.2, Matrix Science) and Sequest search algorithms. Carbamidomethyl cysteine was searched as a fixed modification while oxidized methionine, deamidation of aspargine and glutamine, labels; Lys^13^C_4_, Lys^13^C_6_^15^N_2_, Arg^13^C_6_, and Arg^13^C_6_^15^N_4_ were set as variable modifications. Data from the 2-state SILAC experiment were analyzed to yield light over heavy ratios (CA1a/T1K) for which whole protein ratios were normalized to the corresponding medium over light ratio from the 3-state experiment (T1K/10A). Peptides were identified with a false discovery rate cut-off of 1% and protein ratios were sorted and normalized using Excel (Office 2010, Microsoft). Protein quantitation data is included as Table S1.

Functional annotation clustering was performed for proteins enriched in the cytosol, nuclear and mitochondrial fractions of tumorigenic cell lines using DAVID with the classification stringency at the highest setting [19,20] (Tables S2 and S3). Table 1 was generated to include mitochondrial membrane-related proteins. Raw mass spectrometer output files and data search results from Proteome Discoverer are available for download at the Proteome Commons Tranche repository (https://proteomecommons.org/tranche/) under the project name “MCF10A Progression SILAC”.

**Table 1:** Expression of mitochondrial membrane proteins in cancer progression cells relative to 10A.

		**Whole Cell**			**Mitochondria**		
**Gene**	**Protein Name**	**T1K**	**CA1h**	**CA1a**	**T1K**	**CA1h**	**CA1a**
NDUFA13	NADH dehydrogenase [ubiquinone] 1 alpha	0.52	0.63	0.76	0.56	0.91	1.10
SDHB	Succinate dehydrogenase [ubiquinone]	0.77	0.62	ND	ND	ND	ND
UQCRC1	Complex III subunit 1	0.55	0.57	0.69	0.25	0.62	0.54
UQCRC2	Complex III subunit 2	0.45	0.62	0.52	0.26	0.56	0.64
COX2	Cytochrome c oxidase subunit 2	0.51	0.42	0.64	0.29	0.36	0.54
ATP5A1	ATP synthase subunit alpha	0.68	0.79	0.88	0.41	0.83	0.81
ATP5B	ATP synthase subunit beta	0.67	0.83	0.83	0.40	0.80	0.82
ATP5H	ATP synthase subunit d	0.59	0.76	0.72	0.44	0.81	1.00
ATP5O	ATP synthase subunit O	0.63	0.79	0.76	0.34	0.78	0.63
VDAC1	Outer mitochondrial membrane protein porin 1	0.58	0.56	0.90	0.31	0.55	0.75
VDAC2	Outer mitochondrial membrane protein porin 2	0.66	0.73	1.79	0.42	0.87	1.84
VDAC3	Outer mitochondrial membrane protein porin 3	0.37	0.81	0.57	ND	ND	ND

(*ND = Not Detected)

### Cellular and mitochondrion copy number analysis

Genomic DNA was isolated from cell pellets in triplicate by phenol-chloroform extraction after incubating overnight at 52°C in TNES buffer (10mM Tris pH 8.0, 150 mM NaCl, 2mM EDTA, 0.5% SDS) plus 0.5 mg/ml Proteinase K. DNA concentration of each sample was approximated by absorbance at 260nm UV light, and the average value was used to derive a dilution ratio, such that 5 µL of a 200x dilution of genomic DNA was used per reaction. Quantitative real time polymerase chain reaction (qPCR) was carried out with TaqMan® FAM conjugated gene expression probes for *ALB, RNSP1*, *CS* and *MT7S* genes (Applied Biosystems) on an Agilent Mx3000p QPCR System. Relative quantification was performed by comparative threshold cycle (C_T_) with the threshold set at the exponential phase of the amplification curve. Box plot graphs of the distribution of data points were generated using Microsoft Excel.

### Analysis of OXPHOS, glycolysis and cellular ATP

Cells were seeded in an XF24 cell culture plate to reach 90% confluence, and then equilibrated in unbuffered low-glucose DMEM at 0% CO_2_ for 1 hour prior to analysis. Metabolic response to inhibitors was measured using a Seahorse XF24 Extracellular Flux Analyzer (Seahorse Bioscience) [21], which measures oxidative phosphorylation (OXPHOS) and glycolysis in real time. The apparatus contains two fluorophores, one sensitive to changes in pH and the other sensitive to changes in oxygen concentration. The pH-sensitive fluorophore measures the extracellular acidification rate (ECAR), which is proportional to the rate of lactate production by glycolysis.

The oxygen-sensitive fluorophore measures the oxygen consumption rate (OCR), which enables it to accurately measure the rate at which cytochrome c oxidase (complex IV) reduces one O2 molecule to two H2O molecules during OXPHOS. OCR and ECAR were measured to establish a baseline. Additional automated measurements were performed after the injection of four compounds that affect bioenergetic capacity: oligomycin (1 µM) at injection port A, FCCP (300 nM) at injection port B, 2-DG (100 mM) at injection port C, and rotenone (1 µM) at injection port D. Oligomycin is a complex V inhibitor and measures the amount of oxygen consumption directly attributable to ATP production. FCCP provides information on the maximum respiration capacity. 2-Deoxyglucose blocks glucose utilization in glycolysis and subsequently in the TCA cycle. Some cells show in increase in OCR after 2-DG that might be caused by a shift to a different carbon source such as fatty acids. Finally, rotenone is a complex I inhibitor and completely blocks all respiration; the remaining oxygen consumption is thus from non-mitochondrial sources. The difference between the OCR after oligomycin and rotenone is a direct measure of the relative mitochondrial uncoupling. Data were reported in pmol/min for OCR and mpH/min for ECAR. After the completion of the experiment, cells were immediately trypsinized and counted with the CASY Cell Counter (Innovatis, Bielefeld, Germany) to normalize individual well rate data to cell counts. Each experimental condition was carried out in quintuplicate and the experiment was repeated on three different days. The mean and standard deviation of the three experiments were calculated using Microsoft Excel.

Cellular ATP levels were measured using an ATPlite^TM^ Luminescence Assay System (PerkinElmer). Cells were seeded and treated with inhibitors at the same concentrations used in the Seahorse analysis. Cells were lysed after 45 minutes of treatment with inhibitors and then combined with luciferase and D-Luciferin substrate before being incubated in the dark for 10 minutes. Luminescence was measured using a Synergy 2 microplate reader (BioTek Instruments). Each experimental condition was carried out in quintuplicate and the experiment was repeated on three different days. The mean, standard deviation and statistical significance (by Student’s T-test) were calculated using Microsoft Excel.

### Immunocytochemistry

Cells were grown in an 8-well chamberslide overnight and fixed with 3.7% formalin followed by permeabilization with 0.1% Triton X-100 and blocking with 2.5% BSA for 1 hour. Slides were then incubated overnight at 4°C with primary rabbit monoclonal antibody directed against β1-integrin or mouse monoclonal antibody against mitochondrial ATPase (Mitosciences). Anti-rabbit Alexa Fluor 488 or anti-mouse Alexa Fluor 568 secondary antibodies were added for 2 hours the next day and the cell images were captured on a Nikon 90i microscope at 100x magnification. Stacked images were taken within a 3 µm section at a step of 0.2 µm, and the resulting images were deconvoluted using Volocity Imaging software (Version 5.0). Deconvoluted images were overlaid for FITC and Cy3 channels to observe relative colocalization of the fluorescence indicated by yellow fluorescence in the merged image.

## Results

### SILAC proteomic strategy for the quantification of protein expression and localization

Relative expression and subcellular localization of proteins were measured amongst the breast cancer progression series of cell lines in a multiplexed SILAC experiment. Briefly, ratios from a 2-state experiment were normalized to those of a 3-state experiment conducted in parallel using a common cell line (T1K), similar to previously established methods for normalizing multiple SILAC experiments [22]. After equal numbers of cells were combined and an aliquot was set aside and lysed for whole cell protein analysis, the rest of the cell mixture was fractionated into cytosolic, nuclear and mitochondrial fractions (Figure 1). The purity and the quality of fractionations for mitochondria as well as for nuclear compartments were examined by using Western blot of SILAC whole cell lysate, as well as cytosolic, nuclear and mitochondrial fractions, probed for EGFR and Mitochondrial Porin (Figure S1). Expression ratios across cell lines and for each cellular fraction were grouped by protein (Table S1). In total, 882 proteins were quantified across the MCF10A progression cell lines, of which 476 were quantified across all cell lines at the whole cell level as well as one or more cellular fractions. The relative protein expression in the *HRAS* transformed cell lines, T1K, CA1h and CA1a, was calculated as the expression ratio for each cell line over that of parental 10A cells.

Relative subcellular enrichment and depletion of proteins across cell lines were measured by dividing the protein fold change ratios observed in a given subcellular fraction by the ratio observed in the whole cell lysate. In Figure S2, the proportions of stably localized and specifically delocalized proteins are displayed as pie charts for each cell line. The subcellular flux of proteins with respect to cancer progression is observed as relative enrichment into the cytosolic and mitochondrial fractions with consequent depletion in the nuclear fraction.

Functional annotation clustering analysis was performed to determine whether functional classes of proteins were specifically enriched to a given organelle. This analysis revealed that glycolysis-related and annexin signaling proteins were significantly enriched in the cytosol (Table S2), while mitochondria were enriched with intermediate filaments as well as integrin and ras signaling proteins with cancer progression (Table S3).

### Confirmation of cell counts and determination of relative cellular mitochondrial copy number

In order to confirm the precision of cell input across experiments, multiple aliquots of each cell line were collected during each experiment and were verified by DNA copy number. DNA was extracted from triplicate SILAC cell aliquots (before mixing) of 1.0 x 10^6^ cells, and the relative gene copy number was measured by qPCR. Real-time PCR analysis was carried out such that, for each gene, the exact center of the exponential phase of the fluorescence curve (as determined by the instrument software) was used to calculate a fluorescence value for each gene probe. Gene copy number was then determined as the threshold cycle (C_T_), or the number of thermal cycles necessary to achieve the optimal fluorescence threshold.

**Figure 2:**
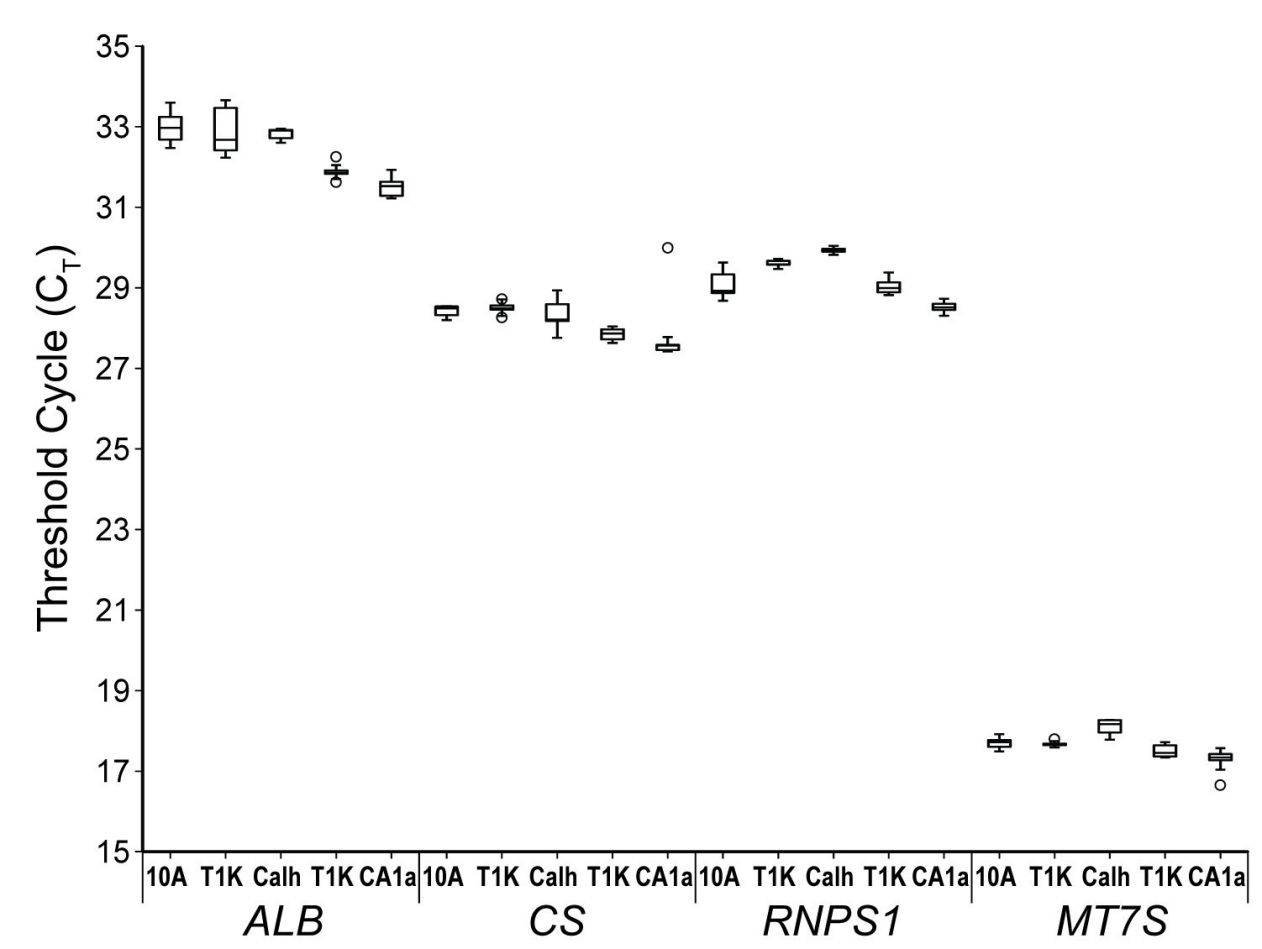
Uniformity of cell ratios in SILAC experiments is confirmed by independent DNA copy number measurements for four genes, albumin (*ALB*) at chromosome locus 4q13.3; RNA-binding protein S1 (RNPS1) at chromosomal locus 16p13.3; citrate synthase (CS) at chromosome locus 12q13.2; mitochondrial D-loop (MT7S) which is the origin of replication for the mitochondrial genome. The exponential phase of the amplification curve was determined using the same parameters for each probe, and gene copy number is reflected as the threshold cycle (CT) at the optimal fluorescence point in the amplification curve determined for that probe. Box plots represent the upper and lower quartiles of the data, with black lines representing the median value and dashed error bars representing standard deviation from the median value. Statistical outliers are represented by open circles.

The gene copy numbers for albumin [23], RNA-binding protein S1 serine-rich domain (*RNSP1*), and citrate synthase (CS) displayed in Figure 2 reflects absolute cell counts as these are all genes within the stable karyotype shared among all four cell lines [3]. Additionally, the relative number of mitochondria per cell line was determined using a probe against the mitochondrial D-loop gene (*MT7S*). Median C_T_ values were consistent within experiments for all three genes, with the greatest variation seen with *RNPS1* for which 10A and CA1h differ by a full cycle. However, the difference between C_T_ values of 29 (10A) and 30 (CA1h) is equivalent to only 3.3% difference in overall cell count. Mitochondrial copy numbers were uniform across cell lines, indicating that this cell line model is not reflective of the breast cancer risk factor of high mitochondrial copy number [23]. This observation supports the use of this model for examining metabolic dysfunction in breast cancer progression that is independent of mitochondrial DNA (mtDNA) copy number.

**Figure 3:**
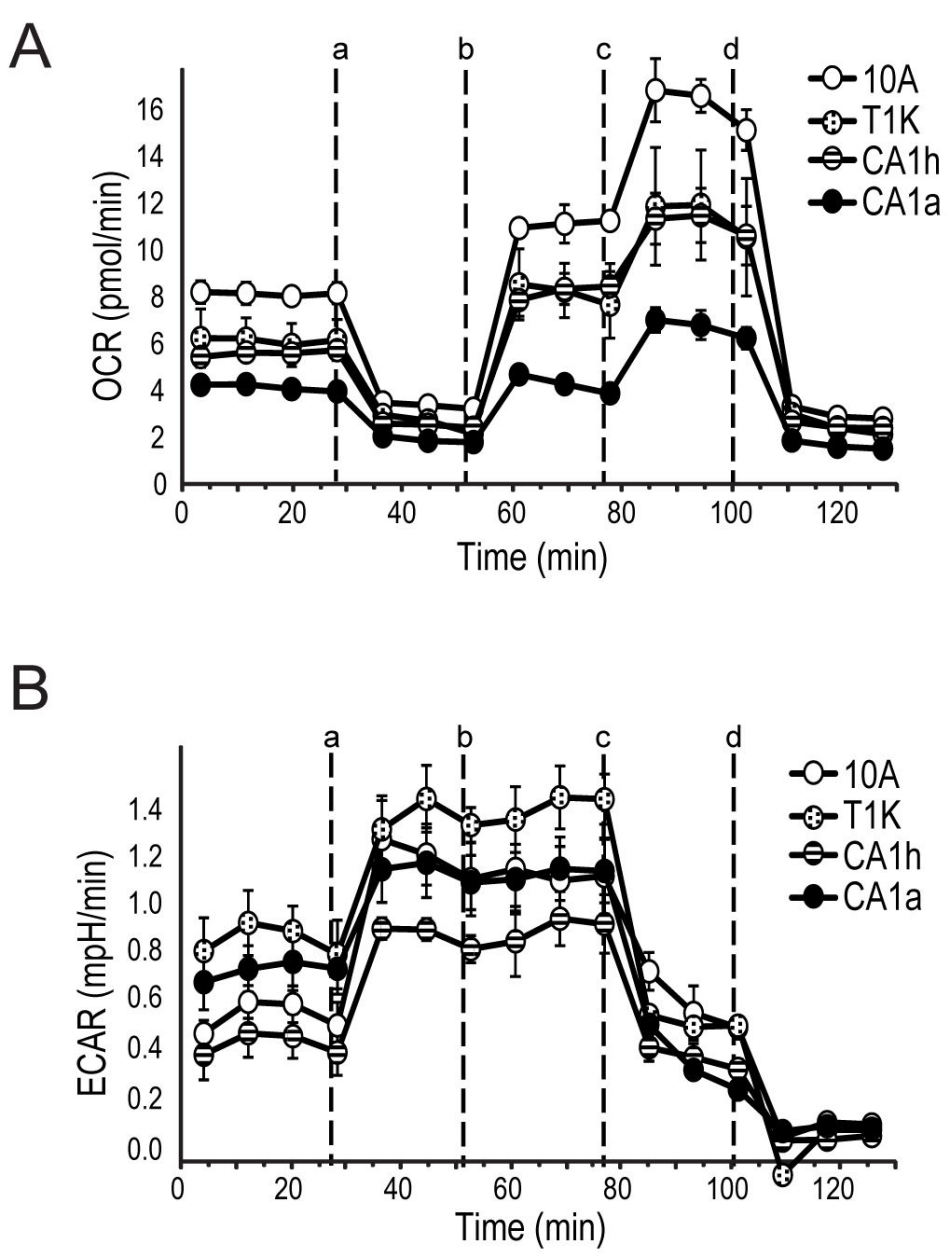
Pharmacological profile of cellular rates of oxygen consumption and lactate production over time with the sequential injection (indicated by dashed lines) of the inhibitors (a) 1 μg/ml oligomycin, (b) 300nM FCCP, (c) 100mM 2-DG, and (d) 1 μM rotenone. A. Oxygen consumption rate (OCR) and B. extracellular acidification rate (ECAR) were measured in a Seahorse XF24 analyzer. Each data-point represents the mean of five independent samples measured in triplicate. Error bars indicate standard deviation of the mean.

### Expression of metabolic enzymes in the MCF10A progression cell lines

While cellular copy number of mtDNA is considered a marker of several diseases, including breast cancer [23], the expression of mitochondrial membrane proteins is a more informative measure of cellular mitochondrial capacity. Several mitochondrial membrane proteins, including pore complex proteins and multiple subunits of the electron transport chain (ETC) complexes, were quantified across all cell lines by our proteomic strategy. Although there was little variation in mtDNA copy number observed across the four cell lines, all three transformed cell lines showed at least a modest reduction compared to parental 10A cells for most mitochondrial membrane proteins (Table 1). These data suggest that *HRAS* transformed MCF10A cells have impaired capacity for OXPHOS, consistent with the findings by Yang et al who observed impaired OXPHOS in *HRAS* transformed NIH-3T3 fibroblasts [13]. However, the greatest reduction in mitochondrial membrane proteins compared to parental cells was observed in the low-grade cancer cell line T1K, while higher levels were observed in CA1h and CA1a cells.

### Assessment of cellular metabolic capacity by pharmacologic modulation

The dynamic expression and subcellular localization of metabolic enzymes in the MCF10A breast cancer progression cell lines reflect a correlation with, and possibly a dependency upon, metabolic dysfunction with cancer progression. To further investigate the differences in metabolic capacity with cancer progression, a Seahorse X24 extracellular flux analyzer (Seahorse Bioscience) was used to measure the cellular oxygen consumption rate (OCR) and the extracellular acidification rate (ECAR). The capacity for or dependency upon OXPHOS and glycolysis for energy metabolism was assessed using a recently developed pharmacological profiling approach [21].

**Figure 4:**
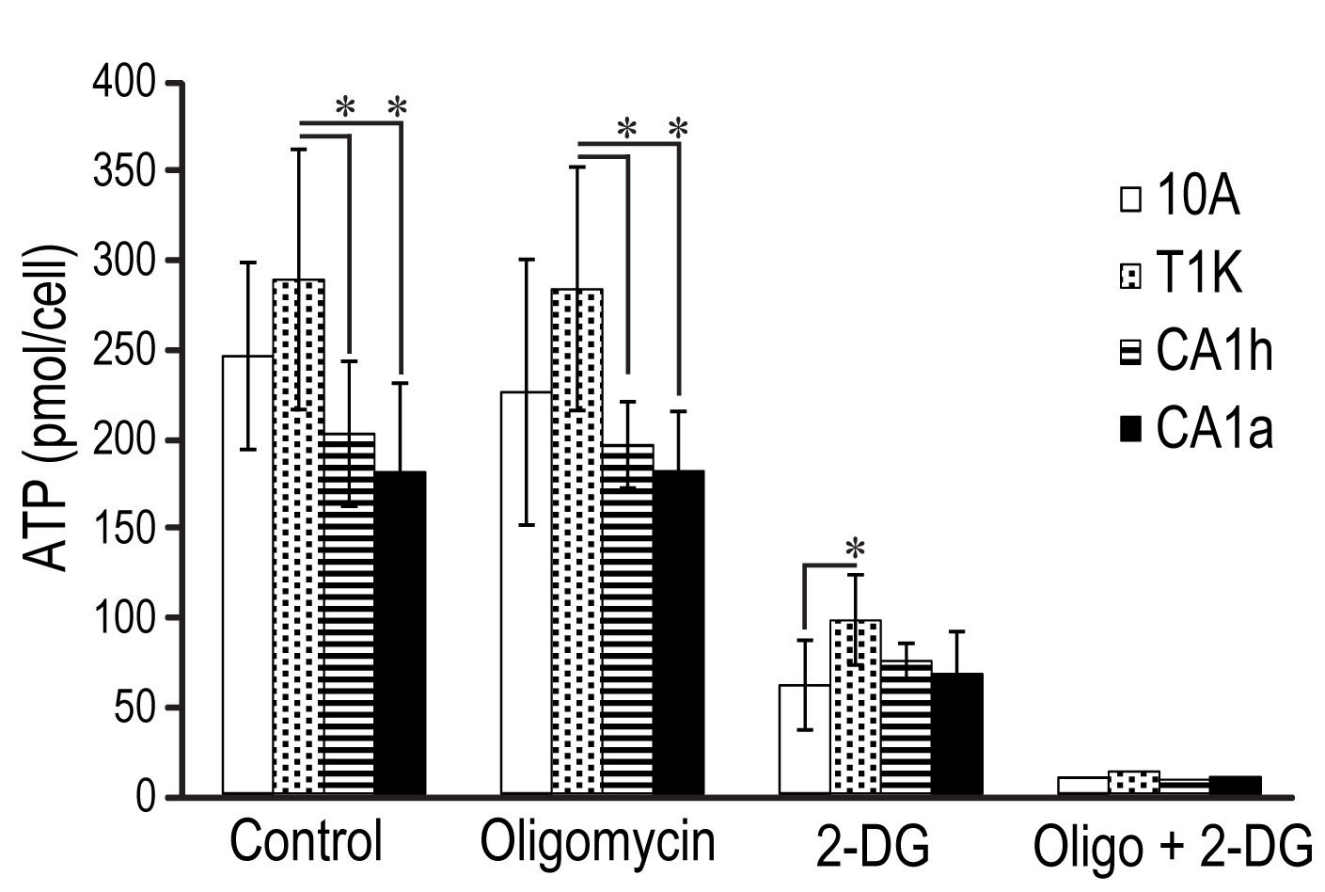
ATP generation relative to cell type and in response to the metabolic inhibitors, oligomycin alone, 2-DG alone, and the combination. Absolute ATP levels were quantified in each cell line. Each bar represents the mean of four independent measurements, and error bars are standard deviation of the mean. Significant differences were determined by comparing values for each cell line to 10A by Wilson’s T-Test (*p<0.05).

**Figure 5:**
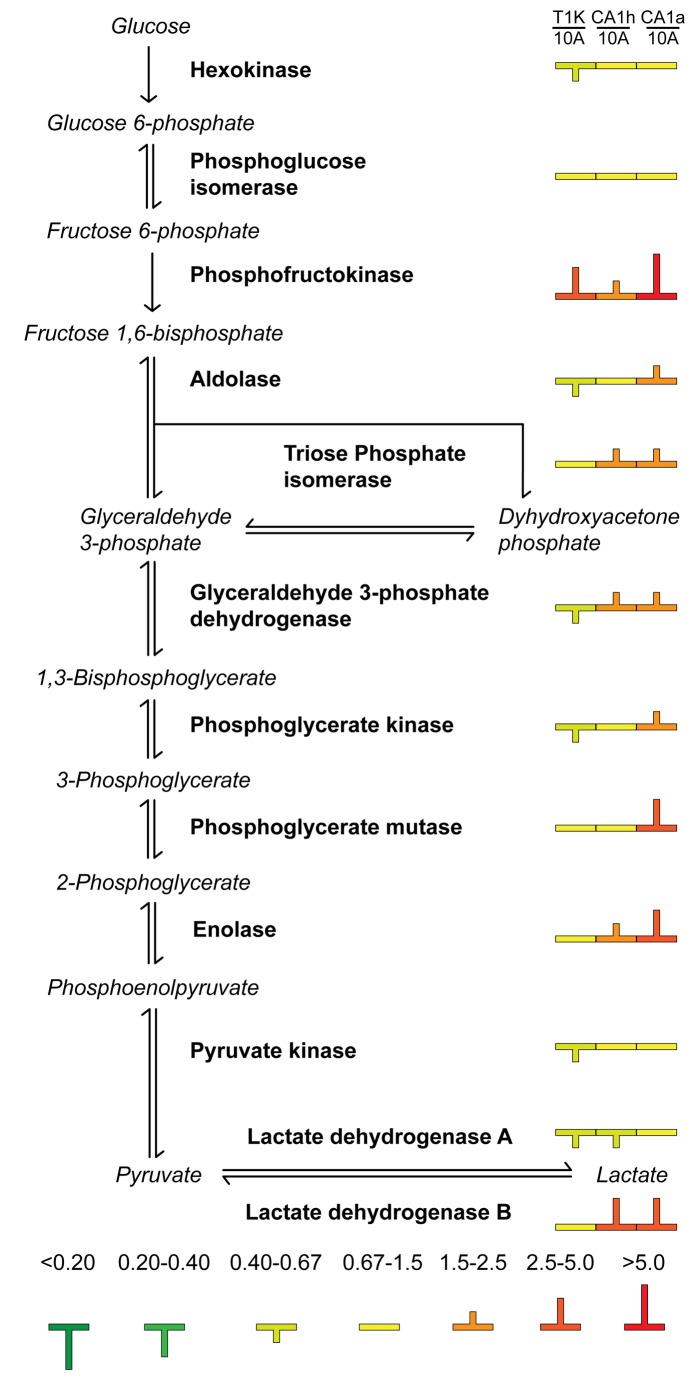
Cytosolic enrichment of glycolytic enzymes with breast cancer progression. Glycolytic enzymes (bold) are featured in the order by which they process intermediate molecules (italic) in the metabolic process of glycolysis. Protein expression in the cytosol is displayed as color-coded symbols to indicate fold-change relative to parental 10A cells.

Basal OCR, a measure of OXPHOS, was observed to decrease with cancer progression with the lowest initial rate of mitochondrial respiration observed in CA1a cells (Figure 3A). Upon application of oligomycin (a), an inhibitor of ATP synthase, all cell lines showed a decrease in OCR and concomitant increase in ECAR, reflecting a shift to glycolysis with inhibition of OXPHOS in the mitochondria. However, upon uncoupling of electron transport and ATP synthesis with FCCP treatment (b), CA1a cells showed the poorest recovery of oxygen consumption, suggesting a greater degree of mitochondrial dysfunction in this cell line. Next, addition of the hexokinase inhibitor 2-DG (c) led to an increase in oxygen consumption that represents the cellular reserve capacity for OXPHOS and again, this capacity is lowest for CA1a cells. Finally, treatment with the complex I inhibitor, rotenone (d), shuts down total mitochondrial electron transport and OCR drops below baseline. These results show that the capacity for OXPHOS decreases with cancer progression in this cell line model.

Basal ECAR, a measure of lactate production from glycolysis, was highest in T1K cells and lowest in CA1h cells (Figure 3B). ECAR rates remain elevated for all four cell lines even after electron transport is restored with FCCP (b), because ATP synthesis remained shut-down from oligomycin. However, the parental 10A cell line showed the greatest proportional increase relative to basal ECAR in response to inhibition of OXPHOS. As expected, ECAR values dropped in response to 2-DG (d) for all cell lines.

Ultimately, the objective of cellular respiration is the generation of ATP to drive the metabolic pathways that enable cell growth and division. In order to link proteomic dynamics and cellular metabolism to this outcome, cellular ATP was determined by measurement of luminescence formed by the ATP-dependent oxidation of D-luciferin by luciferase in cell lysates (Figure 4). Relative proficiency of ATP production was ascribed to mitochondrial or glycolytic means by measuring cellular ATP in the presence of inhibitors specific to each pathway. Oligomycin was used to measure ATP production without a mitochondrial contribution [24], while 2-DG was used to measure ATP synthesis in the absence of glycolytic ATP production, and a combination of oligomycin and 2-DG was used to completely shut down ATP synthesis.

As seen in Figure 4, the absolute concentration of ATP per cell was similar in 10A and T1K cells, but was significantly lower in CA1h and CA1a cells compared to 10A and T1K. This may reflect the fact that CA1h and CA1a have less capacity for OXPHOS but still divide at a faster rate than 10A and T1K cells, and therefore have both a lower rate of ATP production and a higher rate of ATP consumption. Inhibition of glycolysis with 2-DG resulted in a greater than 60% reduction in ATP for all cell lines. These data indicate that 10A cells, and its transformed derivatives, are more dependent on glycolysis for ATP production than mitochondrial OXPHOS. In particular, the ATP levels of parental 10A cells were significantly more affected by 2-DG than the transformed cell lines, despite having the highest reserve capacity for OXPHOS as shown in Figure 3A. Furthermore, oligomycin treatment did not affect any of the four cell lines, suggesting an intact hereditary capacity to respond to mitochondrial dysfunction by compensation of ATP levels through glycolysis.

Although the glycolytic rates as a measure of lactate secretion are counter to the results expected based on the Warburg hypothesis, the global dataset of metabolic enzymes validates these results. As seen in Figure 5, phosphofructokinase, the rate limiting enzyme of glycolysis, is most upregulated in CA1a cells followed by T1K and then last by CA1h. Additionally, levels of LDHB, which is associated with lactate clearance, are highest in CA1h and CA1a cells.

### Dynamic expression and localization of oncoproteins are linked to metabolic dysfunction

The expression of metabolic enzymes and rates of cellular respiration measured in the MCF10A progression series are consistent with the Warburg effect that is characteristic of many cancers, in which cells undergo aerobic glycolysis in lieu of OXPHOS in order to support cellular division. To correlate the expression and activity of non-metabolic proteins with this shift in cellular metabolism, we next examined the expression and subcellular localization of cancer-related proteins that are significantly dysregulated in this cell line model of breast cancer (Table S1). In particular, proteins related to integrin and Ras signaling were significantly enriched in the mitochondria in tumorigenic cell lines (Table S3). As expected, *HRAS*, which was overexpressed in parental MCF10A cells to generate the progression series [2], was highly expressed in all three tumorigenic cell lines and dramatically enriched in the mitochondrial fraction. Several dynamically regulated Ras-related proteins were identified in this study, including RAB7A and RAN, which were increasingly overexpressed at the whole cell level with increased cancer progression. However, examination of changes in protein in the subcellular fractions reflects a much more dynamic regulation of Ras oncoproteins than can be discerned from its total expression levels. For example, the nuclear GTPase, RAN, was increasingly localized to the cytosol in CA1h and CA1a cells, as indicated by cytosolic fold changes that are disproportionately higher than whole cell fold changes and a concomitant reduction in nuclear protein levels. Furthermore, IQGAP1, a CDC42 GTPase effector, is hyper-localized to the cytosol in CA1a cells while whole cell levels are similar to that of 10A. IQGAP1 expression enhances cellular transformation and mobility and has been correlated with reduction in cellular volume, both characteristics of the CA1a cell line [25]. Similarly, RAC1, a Rho GTPase that affects cell migration and chemotaxis by regulation of actin polymerization [26], was increased in CA1h and CA1a mitochondria, but not at the whole cell level.

**Figure 6:**
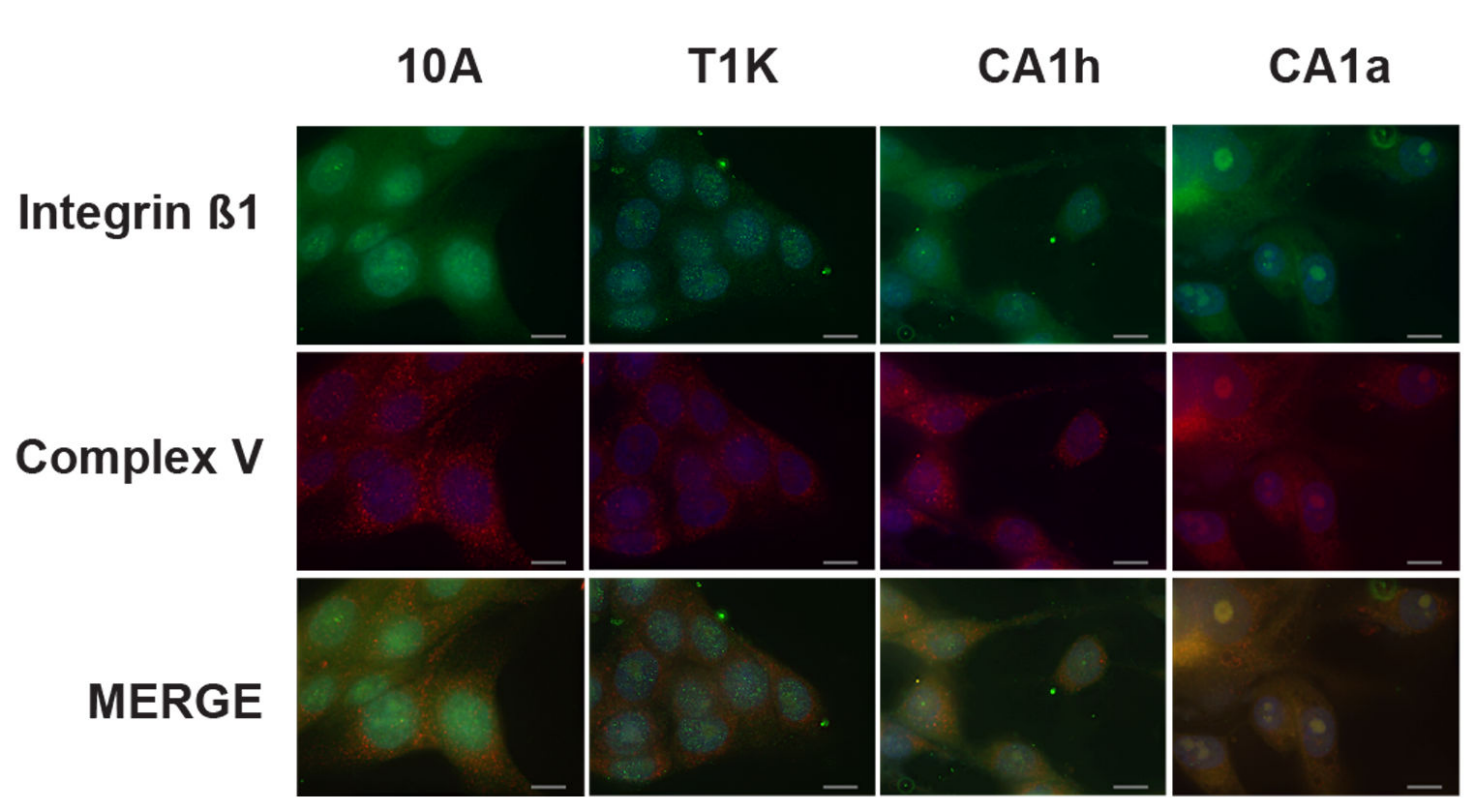
Immunofluorescent colocalization analysis of β1-integrin with mitochondrial ATPase (Complex V). Cells from each of the four lines were formalin fixed and permeabilized on a microscope slide and incubated with monoclonal antibodies raised against mitochondrial ATPase and β1-integrin followed by fluorescent labeled secondary antibody. Expression of Complex V is visualized in red while β1-integrin is in green and co-localization of the two proteins is indicated by yellow color in the merged image. Nuclei are visualized using DAPI (blue) and the gray bars represent 20µm as a reference scale.

Several proteins associated with hypoxia were also up-regulated with progression in these cell lines even though the cells have been chronically cultured in normoxic conditions. This suggests that the hypoxic phenotype is permanently encoded in the malignant cell lines, thus sustaining aerobic glycolysis. It is recognized that, under hypoxic stress, transcriptional activation by hypoxia inducible factor 1 (HIF1) promotes angiogenesis, metastasis, glucose transport and glycolysis and inhibits mitochondrial biogenesis [27,28]. Several proteins that lead to HIF1 stabilization were enriched in the cytosol of CA1h and CA1a cells, including the HIF1 target gene product, prostaglandin-E synthase (PTGES3) [29]. In addition, heat shock protein 90 (HSP90AB1) and multiple subunits of chaperonin containing TCP1 (CCT2, CCT4, CCT5, CCT7 and CCT8) were upregulated in malignant cells, both of which prevent HIF1 degradation by the Von Hippel-Lindau (VHL) factor [30,31].

Perhaps the most striking trend was the localization of integrin and catenin proteins to the mitochondrial and cytosolic fractions in the malignant cell lines. Although previous work has shown that integrins are trafficked through the endosomal pathway [32] and mitochondrial-derived transport vesicles are regulated by components of endosomal transport [33], this is the first evidence of the mitochondrial localization of integrin proteins in cancer. The mitochondrial localization of β1-integrin was validated by immunocytochemistry in Figure 6. The mitochondria were visualized by probing against mitochondrial ATPase (complex V of the electron transport chain) in red and β1-integrin is in green. Mitochondria and integrins appear to be evenly distributed in 10A and T1K cells with limited overlap of the respective puncta. However, in the tumorigenic CA1h and CA1a cells, overlay of the fluorescence is increasingly yellow and some specific punctate structures were observed to colocalize, indicating increasing co-localization of β1-integrin to the mitochondria with cancer progression.

## Discussion

An increased dependence on glycolysis for ATP synthesis as opposed to the more efficient mitochondrial oxidative phosphorylation (OXPHOS) has long been established as a hallmark of carcinogenesis [12]. This subject has received increased attention recently as researchers have more closely considered the role of mitochondrial uncoupling in cancer metabolism and carcinogenesis [34,35] or conversely, the direct role that some oncogenes may serve as metabolic switches [36]. Because previous studies [8-11] of protein expression and signaling disruption in the MCF10A breast cancer progression model of cell lines have demonstrated dysregulation of metabolic proteins, these cell lines were used to interrogate the poorly understood relationship between Ras-driven oncogenesis, metabolism and cellular respiration.

The MCF10A progression series of cell lines is a useful tool to study the process of tumor formation and metastatic progression driven by the *HRAS* oncogene, which is overexpressed in half of all breast cancers [37]. To understand the disruption of cellular signaling and metabolism in this breast cancer progression model, we employed quantitative SILAC proteomic analysis to accurately determine protein expression changes at the cellular and subcellular level. The ultimate benefit of using SILAC based subcellular fractionation is that protein trafficking can be assessed because the inevitable cross-contamination from other organelles is normalized for each cell line. Therefore, when the fold-change difference between cell lines increases or decreases in a specific subcellular fraction relative to the whole cell value, it is indicative of a difference in subcellular enrichment or depletion, respectively, across the cell lines (Figure 1). Using this method, translocation of proteins from one organelle to another can be appreciated, offering insight into potential mechanisms in metabolic dysfunction and metastatic progression beyond that garnered from total protein expression levels. This technique uncovered organelle specific regulation of several proteins involved in cellular respiration, and the observed dysregulation of specific respiratory pathways was consistent with metabolic analysis.

The diverse metabolic profiles of the MCF10A progression series were investigated by comparing the different cell lines for relative mitochondrial copy number, protein expression and localization, capacity for oxidative phosphorylation versus glycolysis, and steady-state ATP levels. All four cell lines had similar expression of mtDNA, while the preneoplastic cell line, T1K, had the lowest expression of mitochondrial membrane proteins, most of which were reduced or unchanged in the malignant CA1h and CA1a cells as well. One notable exception is that the mitochondrial outer membrane protein, porin 2, was up-regulated in CA1a cells whereas porins 1 and 3 were not. Mitochondrial porins 2 and 3 overexpression has been implicated in Ras-activated cancers as the functional target of the anti-cancer drug, erastin [38], which cuts off the supply of ATP from the mitochondria to hexokinase.

The overall reduction in mitochondrial protein expression in the transformed cell lines is indicative of a reduced capacity for OXPHOS in these cells. Some of the proteins of the TCA cycle, which feed NADH to drive OXPHOS, were also impaired. The mitochondrial TCA enzymes (CS, ACO2, DLST, SDHB, FH and MDH2) were somewhat reduced but largely unchanged in transformed cells compared to 10A. Figure 3A demonstrates that basal oxygen consumption rates as well as total cellular capacity of OXPHOS decline with increasing tumor grade. Although reduced capacity in transformed cells compared to parental 10A cells is expected, since all three tumorigenic cell lines have lowered expression of ETC proteins compared to 10A (Table 1), the progressive impairment of OXPHOS is not reflective of the trend in ETC proteins. In fact, ETC protein expression is lowest in T1K cells and only slightly reduced in CA1a cells compared to MCF10A. The observation that CA1a has the lowest capacity for OXPHOS then suggests that electron transport is uncoupled by exogenous factors in the CA1a mitochondria. In this dataset, direct perturbation of the mitochondria is reflected in the mitochondrial enrichment of integrins, catenins and HIF1 target genes.

While mitochondrial TCA enzymes appeared to be largely unchanged, the cytosolic paralogs of these proteins were higher with respect to cancer progression. Cytosolic MDH1 and IDH1 protein expression were both higher in the cytosol of CA1a, and overall expression and cytosolic localization of the cytosolic aconitase (ACO1) was elevated in all three transformed cell lines. High levels of these cytosolic enzymes can draw intermediates from the TCA cycle toward the production of pyruvate as part of pyruvate-malate or pyruvate-isocitrate cycling in CA1h and CA1a cells. The ECAR measurements in Figure 3B indicate that T1K cells have the highest lactate secretion, 10A and CA1a cells are similar, and CA1h cells have the lowest lactate secretion, but are contrary to the predicted trends in glycolytic activity based on the observed OCR values and expression of phosphofructokinase. One contributing factor leading to decreased ECAR in the aggressive cancer cells relative to the preneoplastic T1K cells is the increase in LDHB (Figure 5). Higher LDHB will elevate the stoichiometry of LDHB to LDHA, leading to the formation of more LDH1 and LDH2 tetramers that have a preference for conversion of lactate to pyruvate in a reverse reaction, which can then feed cellular respiration through the TCA cycle [39].

Furthermore, CA1h and CA1a mitochondria were found to have increased expression of the hypoxia-inducible, tumor-associated lactate/proton symporter, monocarboxylate transporter 4 (MCT4), as well as the MCT4 cofactor, basigin, that is required for its activity [40]. These data strongly suggest that, in the CA1h and CA1a cells, cellular lactate is pumped back into the mitochondria to be converted to pyruvate and fed into the TCA cycle. With levels of pyruvate carboxylase that are higher in CA1h compared to CA1a and the weaker capacity for OXPHOS seen in CA1a, it is reasonable that CA1h cells can more efficiently clear lactate through this process.

Our analysis provides the first evidence that some oncogenic signaling proteins are increasingly trafficked to the mitochondria in malignant cells, offering key insight into their role in the Warburg effect of carcinogenesis. The increase in mitochondrial integrins coupled with decreasing OXPHOS in concert with tumor cell grade is consistent with the recent report that siRNA knock-down of β4-integrin rescues cell senescence in aged cardiac myocytes, in part by restoring mitochondrial function [41]. The exact role of this unique integrin signaling in cancer progression and metabolic dysfunction requires further study, but the advantage of using quantitative subcellular proteomics to elucidate these mechanisms is substantial. The increase in mitochondrial integrins coupled with decreasing OXPHOS in concert with tumor cell grade is consistent with the recent report that siRNA knock-down of β4-integrin rescues cell senescence in aged cardiac myocytes, in part by restoring mitochondrial function [41]. The exact role of this important oncoprotein in cancer progression and metabolic dysfunction require further study, but the advantage of using quantitative subcellular proteomics to elucidate these mechanisms is substantial. Finally, the combination of proteomic and metabolic analysis applied to this model system provides a foundation for the integrated analysis of metabolic dysfunction in cancer.

## Supporting Information

Table S1 **Protein fold change relative to parental 10A cells at whole cell, cytosol, nuclear and mitochondrial level.**
(PDF)

Table S2 **Functional annotation clustering of cytosol enriched proteins.**
(PDF)

Table S3 **Functional annotation clustering of mitochondrial enriched proteins.**
(PDF)

Figure S1 **Western blot of approximately 25 µg of SILAC whole cell lysate, as well as cytosolic, nuclear and mitochondrial fractions, probed for EGFR and Mitochondrial Porin.**
(TIF)

Figure S2 **Quantitative delocalization of proteins relative to MCF10A parental cells.** Relative enrichment of proteins was calculated by dividing fold change ratios within subcellular fractions by fold change ratios at the whole cell level to determine if changes in subcellular stoichiometries were indicative of protein delocalization (colors) or if the differences were consistent with changes in whole cell protein expression (gray color). Proteins were considered to be delocalized if there was greater than a 50% difference between the subcellular fraction ratio and the whole cell lysate ratio.
(TIF)
