# Supplementary material for: Integrated Proteomic and Metabolic Analysis of Breast Cancer Progression
Source: PLoS One. 2013 Sep 27;8(9):e76220. doi: 10.1371/journal.pone.0076220 (PMC3785415; doi:10.1371/journal.pone.0076220)
Supplement: Table S1 — Protein fold change relative to parental 10A cells at whole cell, cytosol, nuclear and mitochondrial level. (PDF) [file pone.0076220.s001.pdf]

Table S1. Protein fold change relative to parental 10A cells at whole cell, cytosol, nuclear and mitochondrial level

			Whole Cell			Cytosol			Nucleus			Mitochondria		
**Accession**	**Gene**	**Protein Name**	**T1K**	**CA1h**	**CA1a**	**T1K**	**CA1h**	**CA1a**	**T1K**	**CA1h**	**CA1a**	**T1K**	**CA1h**	**CA1a**
NP_003371.2	VIM	vimentin	28.73	1.00	27.78				29.58	1.00	19.76	34.13	1.00	64.95
NP_005334.1	HRAS	GTPase HRas isoform 1	5.73	2.41	12.86				4.61	5.38		5.01	16.78	81.53
NP_001003.1	RPS8	40S ribosomal protein S8	4.73	1.25	11.15	5.89	10.21	14.15	0.80	1.17	0.97	0.49	0.98	1.84
NP_002195.1	ITGA3	integrin alpha-3 isoform a precursor	1.31	1.60	8.95							0.54	2.64	3.33
NP_006398.1	ARL6IP5	PRA1 family protein 3	0.34	1.30	8.81									
NP_003079.1	FSCN1	fascin	2.37	2.43	6.80	2.79	3.48	13.08						
NP_002452.1	MVD	diphosphomevalonate decarboxylase	0.55	0.48	6.63									
NP_006507.2	SLC2A1	solute carrier family 2, facilitated glucose transporter member 1	1.77	1.55	6.17									
NP_004719.2	DDX21	nucleolar RNA helicase 2	1.52	2.02	5.90									
NP_006133.1	SFN	14-3-3 protein sigma	1.17	0.51	5.67	1.40	0.72	10.37	1.30	0.42	3.15	0.72	0.62	6.04
NP_004095.4	FASN	fatty acid synthase	0.87	1.68	5.67	0.92	2.06	9.03	0.84	1.59	2.70	0.55	1.48	4.87
NP_863656.1	ACOT7	cytosolic acyl coenzyme A thioester hydrolase isoform hBACHd	1.42	1.64	5.09									
NP_006614.2	PHGDH	D-3-phosphoglycerate dehydrogenase	2.51	3.53	4.54	42.75	1.00	152.26	3.18	2.34	3.64	1.91	2.31	5.36
NP_001005731.1	ITGB4	integrin beta-4 isoform 3 precursor	3.64	2.13	4.49	6.56	11.17	39.02				2.08	4.77	9.99
NP_060596.2	TMEM33	transmembrane protein 33	1.81	2.66	4.37				1.33	1.89		0.50	1.43	
NP_001073867.1	KIAA0368	proteasome-associated protein ECM29 homolog	2.34	1.72	4.24	1.84	2.87	5.02						
NP_004628.4	RAB7A	ras-related protein Rab-7a	0.95	1.18	4.08				6.91	9.92	75.94	0.40	1.14	1.76
NP_004578.2	RRBP1	ribosome-binding protein 1	1.07	1.91	4.08				1.02	2.01	1.21			
NP_476516.1	NAA15	N-alpha-acetyltransferase 15, NatA auxiliary subunit	1.81	1.07	3.93	1.37	2.04	6.14	1.36	1.84				
NP_002902.2	UPF1	regulator of nonsense transcripts 1	1.26	1.60	3.85	1.48	1.56							
NP_055335.2	MYBBP1A	myb-binding protein 1A isoform 2	1.39	0.97	3.75									
NP_004944.3	EIF4G1	eukaryotic translation initiation factor 4 gamma 1 isoform 4	0.84	1.49	3.73									
NP_000201.2	ITGA6	integrin alpha-6 isoform b precursor	3.25	2.59	3.72							3.19	12.64	17.91
NP_001530.1	DNAJA1	dnaJ homolog subfamily A member 1	1.24	2.88	3.61	1.44	4.86						3.09	
NP_037366.1	EIF3K	eukaryotic translation initiation factor 3 subunit K	1.55	1.77	3.52				1.33	1.58	1.74	0.84	1.37	4.45
NP_031381.2	HSP90AB1	heat shock protein HSP 90-beta	1.27	2.09	3.52	1.46	2.83	6.45	1.22	1.94	2.08	0.78	1.70	2.76
NP_001116841.1	PPP6C	serine/threonine-protein phosphatase 6 catalytic subunit isoform c	1.03	1.31	3.42									
NP_003741.1	EIF3A	eukaryotic translation initiation factor 3 subunit A	1.25	1.40	3.36	1.23	1.85	4.59						
NP_005333.2	HMGB3	high mobility group protein B3	12.52	2.28	3.34									
NP_001129477.1	GART	trifunctional purine biosynthetic protein adenosine-3 isoform 1	1.50	1.46	3.24	1.33	1.65	4.77	1.68	1.37	2.36			
NP_001020605.1	NQO1	NAD(P)H dehydrogenase [quinone] 1 isoform c	1.37	8.06	3.24	1.57	9.47	4.91	1.58	8.46			6.49	
NP_079422.1	NAA50	N-alpha-acetyltransferase 50, NatE catalytic subunit	1.05	1.37	3.22				1.13	1.21				
NP_002262.3	IPO5	importin-5	1.25	1.46	3.12	1.27	1.79	24.09						
NP_001186071.1	EIF3C	eukaryotic translation initiation factor 3 subunit C	1.04	1.36	3.11									
NP_002559.2	PABPC1	polyadenylate-binding protein 1	0.92	1.49	3.10									
NP_006161.2	NOP2	putative ribosomal RNA methyltransferase NOP2	1.21	2.16	3.06									
NP_001407.1	EIF4A1	eukaryotic initiation factor 4A-I isoform 1	1.13	1.78	3.04	1.38	2.48							
NP_002622.2	PGD	6-phosphogluconate dehydrogenase, decarboxylating	0.78	1.48	3.03	0.90	1.90	34.16	1.13	1.29	2.26	0.53	1.27	3.47
NP_005780.2	PSME3	proteasome activator complex subunit 3 isoform 1	1.18	1.23	2.99	0.90	2.68	2.65	0.99	1.43	1.79			
NP_002188.1	ACO1	cytoplasmic aconitate hydratase	2.08	5.08	2.99	2.95	7.88	8.73	3.28	7.90				
NP_000875.2	IMPDH2	inosine-5'-monophosphate dehydrogenase 2	0.97	0.76	2.98									

NP_002799.3	PSMD2	26S proteasome non-ATPase regulatory subunit 2	1.07	1.28	2.95	1.31	1.83	4.50	0.95	1.22	1.54	0.57	1.03	2.44
NP_004085.1	EIF2S1	eukaryotic translation initiation factor 2 subunit 1	1.16	1.21	2.93									
NP_038203.2	IARS	isoleucyl-tRNA synthetase, cytoplasmic	1.32	1.60	2.91	1.57	2.01	4.40						
NP_000691.1	ANXA1	annexin A1	1.42	1.37	2.89	1.96	2.14	5.44	1.40	1.16	1.51	1.03	1.47	2.47
NP_001395.1	EEF1G	elongation factor 1-gamma	1.13	1.25	2.89	1.23	1.80	4.92	1.07	1.17	1.34	0.73	0.92	2.75
NP_006382.1	IPO7	importin-7	1.15	0.66	2.88	1.03	0.80	4.95						
NP_005678.3	FARSB	phenylalanyl-tRNA synthetase beta chain	1.54	1.07	2.86									
NP_006592.3	PTGES3	prostaglandin E synthase 3	1.34	2.33	2.85				1.09	1.92				
NP_003742.2	EIF3B	eukaryotic translation initiation factor 3 subunit B	1.55	1.80	2.85				1.47	1.51	1.52			
NP_004332.2	CAD	CAD protein	1.29	1.39	2.83	1.30	1.88	28.28						
NP_036205.1	CCT5	T-complex protein 1 subunit epsilon	1.44	1.39	2.82	1.10	1.46	4.20	1.15	1.49	1.43			
NP_002291.1	LDHB	L-lactate dehydrogenase B chain	1.25	2.66	2.78	1.35	3.79	4.31	1.14	2.34	1.32	0.78	2.71	2.12
NP_005585.1	NACA	nascent polypeptide-associated complex subunit alpha isoform b	1.21	1.75	2.75	1.06	2.27	3.75						
NP_006421.2	CCT4	T-complex protein 1 subunit delta	1.19	1.23	2.73	1.00	1.76	3.88	1.18	1.25	1.43			
NP_056988.3	EIF5B	eukaryotic translation initiation factor 5B	0.87	1.69	2.72									
NP_478126.1	EXOSC6	exosome complex component MTR3	1.12	0.84	2.71									
NP_001032752.1	EEF1B2	elongation factor 1-beta	1.06	1.16	2.71	1.11	1.55	4.86	1.13	1.29	1.53		0.91	
NP_000261.2	PNP	purine nucleoside phosphorylase	0.96	1.01	2.68	1.32	1.68	6.47						
NP_000414.2	KRT2	keratin, type II cytoskeletal 2 epidermal	0.07	0.07	2.65							0.05	0.05	4.32
NP_001952.1	EEF2	elongation factor 2	0.97	1.25	2.65	1.16	1.72	4.86	0.90	1.09	1.27	0.41	0.92	1.66
NP_114381.1	EIF4H	eukaryotic translation initiation factor 4H isoform 2	1.22	1.16	2.64									
NP_006182.2	PA2G4	proliferation-associated protein 2G4	1.23	1.80	2.63	1.42	2.61							
NP_002793.2	PSMC1	26S protease regulatory subunit 4	1.08	1.17	2.62									
NP_002009.1	FLII	protein flightless-1 homolog	1.15	1.01	2.61	0.85	1.65	13.63						
NP_000980.1	RPL30	60S ribosomal protein L30	1.47	1.22	2.59									
NP_064587.1	NIT2	omega-amidase NIT2	0.89	0.85	2.58									
NP_001307.2	CSE1L	exportin-2	1.15	1.28	2.58	1.19	1.54	4.68	1.07	1.13	1.49			
NP_037473.3	OLA1	obg-like ATPase 1 isoform 1	0.98	1.34	2.58	0.16	1.77	1.09	0.86	1.15	1.17			
NP_002145.3	HSPA4	heat shock 70 kDa protein 4	1.19	1.39	2.55	1.26	1.88	4.30	1.27	1.26	1.42	0.81	1.38	2.67
NP_001029025.1	RPL3	60S ribosomal protein L3 isoform b	1.15	1.51	2.50	1.31	2.10		1.13	1.31	1.22			
NP_001001.2	RPS6	40S ribosomal protein S6	1.00	1.31	2.46	1.08	2.18	3.85	1.23	1.49	1.75	0.39	1.03	1.48
NP_001159757.1	CCT7	T-complex protein 1 subunit eta isoform d	1.05	1.26	2.44	1.16	1.70	4.23	1.00	1.25	1.21			
NP_056473.2	NOC2L	nucleolar complex protein 2 homolog	1.72	1.26	2.43									
NP_003320.2	TXN	thioredoxin	1.26		2.42									
NP_004437.2	EPRS	bifunctional aminoacyl-tRNA synthetase	1.03	1.33	2.40	1.06	1.53	3.38	0.74	0.57	0.90			
NP_006588.1	HSPA8	heat shock cognate 71 kDa protein isoform 1	0.95	1.22	2.40	1.21	1.93	4.61	1.01	1.24	1.40	0.63	1.27	2.29
NP_000969.1	RPL23	60S ribosomal protein L23	1.11	1.38	2.35				0.92	1.20	0.96	0.63	1.23	2.18
NP_009057.1	VCP	transitional endoplasmic reticulum ATPase	1.21	1.69	2.35	1.67	2.47	4.94	1.27	1.71	1.43	0.64	1.28	2.24
NP_072045.1	RPS18	40S ribosomal protein S18	1.09	1.29	2.31				1.12	1.31	1.24	0.55	0.87	2.03
NP_002583.1	PCNA	proliferating cell nuclear antigen	0.92	1.23	2.30	1.38	2.47	4.61	1.11	1.35	1.79			
NP_004490.2	HNRNPAB	heterogeneous nuclear ribonucleoprotein A/B isoform b	1.04	1.24	2.29									
NP_000996.2	RPS3	40S ribosomal protein S3	0.97	1.24	2.28	0.96	1.56	2.70	0.95	1.27	1.18	0.39	0.92	1.31
NP_001009.1	RPS15	40S ribosomal protein S15	1.13	1.37	2.27				0.11	1.09	0.15	0.39	0.97	1.31
NP_596867.1	ITGB1	integrin beta-1 isoform 1A precursor	1.12	1.13	2.26	0.88	2.25	7.40				0.77	2.77	6.03
NP_004913.2	SEC24C	protein transport protein Sec24C	0.79	0.79	2.25									
NP_002511.1	NPM1	nucleophosmin isoform 1	1.21	1.18	2.25				1.25	1.20	1.37			
NP_057212.1	COPG	coatomer subunit gamma	0.96	1.43	2.25	0.97	1.59		0.84	1.37	1.01			

NP_000963.1	RPL7A	60S ribosomal protein L7a	0.98	1.16	2.23	1.12	1.69	3.06	0.97	1.32	1.20	0.42	0.83	1.51
NP_001180346.1	DDX3X	ATP-dependent RNA helicase DDX3X isoform 3	1.08	1.36	2.23									
NP_006296.1	ANP32A	acidic leucine-rich nuclear phosphoprotein 32 family member A	0.99	1.11	2.22	1.24	1.72	3.82	1.05	1.29	1.07			
NP_006635.2	HSPH1	heat shock protein 105 kDa	1.18	1.66	2.22	1.68	2.55	5.18						
NP_001012321.1	RPSA	40S ribosomal protein SA	1.08	1.26	2.22	1.19	1.48		7.96	1.16	8.64	0.50	0.92	1.54
NP_573566.2	LRPPRC	leucine-rich PPR motif-containing protein, mitochondrial precursor	1.05	1.24	2.21								1.14	
NP_112240.1	ARPC5L	actin-related protein 2/3 complex subunit 5-like protein	1.45	1.11	2.21									
NP_004598.1	TBCA	tubulin-specific chaperone A	0.97	1.30	2.19									
NP_002782.1	PSMA6	proteasome subunit alpha type-6	1.19	1.51	2.19	1.01	1.77	3.03	1.01	1.46				
NP_009123.1	SUPT16H	FACT complex subunit SPT16	1.27	1.16	2.19				0.84	0.96	0.90			
NP_000972.1	RPL19	60S ribosomal protein L19	1.00	1.11	2.17				1.18	1.11	1.20	0.12	1.70	0.39
NP_000983.1	RPL29	60S ribosomal protein L29	0.95	1.16	2.16				0.86	1.11	1.04	0.38	0.81	1.37
NP_001393.1	EEF1A1	elongation factor 1-alpha 1	0.81	0.94	2.15	0.89	1.28	4.28	0.70	0.88	1.09	0.44	0.64	2.08
NP_002777.1	PSMA1	proteasome subunit alpha type-1 isoform 2	1.01	1.52	2.15	1.13	1.77	3.08	0.97	1.41	1.17			
NP_004930.1	DDX1	ATP-dependent RNA helicase DDX1	0.91	0.82	2.14									
NP_055629.1	PSMD6	26S proteasome non-ATPase regulatory subunit 6	0.95	1.16	2.14									
NP_001186211.1	LRBA	lipopolysaccharide-responsive and beige-like anchor protein	0.73	0.84	2.14	0.88	1.57	8.85						
NP_444505.1	RPLP0	60S acidic ribosomal protein P0	0.82	1.35	2.13	0.45	0.73							
NP_078934.3	IPO4	importin-4	1.26	1.46	2.11	1.43	1.92	4.71						
NP_005608.1	RPS14	40S ribosomal protein S14	1.05	1.36	2.11				1.02	1.29	1.21	0.55	1.01	1.94
NP_000977.1	RPL24	60S ribosomal protein L24	0.92	1.10	2.10				0.89	1.19	1.17	0.76	0.89	2.68
NP_005339.3	HSP90AA1	heat shock protein HSP 90-alpha isoform 2	1.10	1.66	2.10	1.29	2.20	3.88	1.12	1.60	1.46	0.82	1.47	2.19
NP_000998.1	RPS4X	40S ribosomal protein S4, X isoform X isoform	0.95	1.25	2.10	1.09	1.85	2.91	0.88	1.17	1.06	0.54	0.98	1.83
NP_001185986.1	RPS17L	40S ribosomal protein S17-like	0.98	1.41	2.10	0.02	0.02	0.05	0.97	1.20	1.08			
NP_057154.2	MRPS23	28S ribosomal protein S23, mitochondrial	0.97	0.69	2.10									
NP_036555.1	RPL13A	60S ribosomal protein L13a	1.04	1.35	2.09				0.85	1.17	1.04	0.44	0.74	1.70
NP_110382.3	TMX1	thioredoxin-related transmembrane protein 1 precursor	1.24	1.01	2.09									
NP_150254.1	RPL13	60S ribosomal protein L13 isoform 1	1.01	1.19	2.08	1.19	1.67	3.16	1.11	1.22	1.21			
NP_148982.1	RPS24	40S ribosomal protein S24 isoform a	1.08	1.11	2.08									
NP_001008.1	RPS13	40S ribosomal protein S13	0.94	1.20	2.07	5.70	1.20	15.55	0.85	1.42	1.05	0.43	0.92	1.49
NP_001177966.1	PSMD1	26S proteasome non-ATPase regulatory subunit 1 isoform 2	1.09	1.29	2.06	1.13	1.63	4.11						
NP_055950.1	NUP205	nuclear pore complex protein Nup205	1.18	0.72	2.06									
NP_006004.2	RPL10	60S ribosomal protein L10	1.10	1.19	2.06									
NP_003364.1	VCL	vinculin isoform VCL	0.66	1.02	2.06	0.64	1.22	2.81	0.63	0.84	1.19		1.07	
NP_687033.1	PSMA3	proteasome subunit alpha type-3 isoform 2	1.17	1.47	2.06	1.33	2.00	3.37	0.71	1.05	0.72	0.57	0.97	1.96
NP_001157791.1	FLNB	filamin-B isoform 4	1.06	0.47	2.06	1.08	0.75	4.76	1.10	0.44	1.27	0.77	0.74	4.19
NP_056455.3	SERBP1	plasminogen activator inhibitor 1 RNA-binding protein isoform 4	0.94	1.20	2.05	1.23	1.58	3.08	0.99	1.35	1.26			
NP_000960.2	RPL5	60S ribosomal protein L5	0.99	1.13	2.05	1.05	1.51	2.40	0.79	1.11	1.02	0.52	0.79	1.87
NP_006576.2	CCT8	T-complex protein 1 subunit theta	1.09	1.58	2.04	1.15	1.67	4.49	1.23	0.14	1.85			
NP_002789.1	PSMB6	proteasome subunit beta type-6 precursor	1.15	1.35	2.03				1.05	1.24				
NP_002127.1	HNRNPA1	heterogeneous nuclear ribonucleoprotein A1 isoform a	0.97	1.14	2.03	1.25	1.53	2.35	0.92	1.22	1.01	0.64	1.07	1.41
NP_000971.1	RPL18A	60S ribosomal protein L18a	0.94	1.73	2.02				0.88	1.16		0.40	0.91	1.36

NP_000984.1	RPL31	60S ribosomal protein L31 isoform 1	1.01	1.12	2.02				0.70	0.91	0.72	0.50	0.92	
NP_002943.2	RPS2	40S ribosomal protein S2	0.89	1.17	2.02	1.05	1.64	3.19	0.95	1.21	1.31	0.42	0.82	1.45
NP_001002.1	RPS7	40S ribosomal protein S7	0.87	1.20	2.01	1.04	1.77	2.62	0.84	1.21	0.98	0.39	0.85	1.26
NP_001951.2	EEF1D	elongation factor 1-delta isoform 2	0.85	0.82	2.01									
NP_004406.2	DSP	desmoplakin isoform I	0.60	0.15	2.00				0.63		0.74			
NP_001367.2	DYNC1H1	cytoplasmic dynein 1 heavy chain 1	0.81	0.92	2.00	0.84	1.30	3.72	1.11	0.86	1.29	0.51	0.68	2.43
NP_036565.2	SF3B1	splicing factor 3B subunit 1 isoform 1	0.98	1.19	2.00									
NP_009035.3	RPL10A	60S ribosomal protein L10a	1.01	1.22	2.00	0.97	1.68	2.20	0.89	1.20	1.10			
NP_004300.1	ARHGDIA	rho GDP-dissociation inhibitor 1 isoform a	0.75	0.73	2.00	0.92	1.09	3.76	0.72	0.66	1.04			
NP_001128527.1	TKT	transketolase	0.75	1.08	1.99	1.09	1.81	3.81	0.75	0.91	1.09	0.60	1.03	2.65
NP_001030168.1	RPL14	60S ribosomal protein L14	1.02	1.18	1.98	0.82	1.43	1.95	0.89	1.08	1.30	0.46	0.81	1.67
NP_006286.1	VARS	valyl-tRNA synthetase	0.70	1.00	1.97	0.81	1.24	3.59	1.04	0.96	1.72	0.15	0.19	0.59
NP_004362.2	COPA	coatomer subunit alpha isoform 2	0.89	1.08	1.95	0.85	1.73	2.44						
NP_001030178.1	RPL17	60S ribosomal protein L17 isoform a	0.89	1.28	1.95				0.88	1.17	0.97	0.46	0.80	
NP_001019.1	RPS25	40S ribosomal protein S25	0.88	1.23	1.95				0.95	1.26	1.12			
NP_004672.2	EIF1AY	eukaryotic translation initiation factor 1A, Y-chromosomal	0.94	0.89	1.94				1.06	0.86				
NP_003698.1	RUVBL1	ruvB-like 1	1.01	1.27	1.92	1.22	1.56	3.66				0.87	1.24	2.85
NP_000997.1	RPS3A	40S ribosomal protein S3a	0.97	1.24	1.92	1.05	1.81	2.84	0.89	1.19	1.09	0.42	0.93	1.41
NP_001020092.1	RPL9	60S ribosomal protein L9	0.92	1.08	1.92				0.87	1.14	0.96	0.42	0.80	2.27
NP_001020.2	RPS26	40S ribosomal protein S26	0.86	1.22	1.92	0.93	1.35	2.57				0.47	0.87	1.48
NP_000981.1	RPL27A	60S ribosomal protein L27a	0.95	1.16	1.91				1.01	1.19	1.05	0.40	0.89	1.21
NP_006422.1	CCT2	T-complex protein 1 subunit beta isoform 1	0.88	1.23	1.91	0.89	1.54	3.47						
NP_000975.2	RPL23A	60S ribosomal protein L23a	0.90	1.20	1.91				0.79	1.10	0.92	0.46	0.91	1.65
NP_001553.1	IL18	interleukin-18 isoform 1 proprotein	0.59	0.59	1.91				0.03	0.03				
NP_001072992.1	PAICS	multifunctional protein ADE2 isoform 2	0.94	1.12	1.90				0.71	0.83	0.72			
NP_001027017.1	CPT1A	carnitine O-palmitoyltransferase 1, liver isoform isoform 2	1.32	1.57	1.89									
NP_001014.1	RPS20	40S ribosomal protein S20 isoform 2	0.96	1.26	1.89				1.04	1.18	1.27	0.44	0.99	1.50
NP_000959.2	RPL4	60S ribosomal protein L4	0.87	1.16	1.89	1.20	1.78		0.89	1.15	1.03	0.51	0.78	1.94
NP_036457.1	MAPRE1	microtubule-associated protein RP/EB family member 1	0.76	0.87	1.89	0.78	1.12	0.52	0.80	0.93	1.01			
NP_001004.2	RPS9	40S ribosomal protein S9	1.04	1.22	1.88				0.99	1.27	1.16	0.61	1.13	1.76
NP_150644.1	RPL8	60S ribosomal protein L8	0.94	1.19	1.88	1.14	1.69	2.91	0.93	1.16	1.06	0.45	0.84	1.40
NP_009140.1	RPL35	60S ribosomal protein L35	0.92	1.34	1.88							0.44	0.91	
NP_006827.1	GCN1L1	translational activator GCN1	0.88	1.76	1.87	1.05	2.45	3.33						
NP_001010.2	RPS15A	40S ribosomal protein S15a	0.93	1.28	1.87				0.88	1.24	1.06	0.45	0.79	1.62
NP_001108628.1	GDI2	rab GDP dissociation inhibitor beta isoform 2	0.81	0.96	1.87	1.19	1.43	4.25	0.91	1.32	1.23			
NP_006112.3	KRT1	keratin, type II cytoskeletal 1	0.04	0.04	1.87				0.01	0.01	0.77	0.03	0.03	2.45
NP_000967.1	RPL12	60S ribosomal protein L12	0.88	1.11	1.87				0.85	1.10	1.03	0.46	0.79	1.36
NP_001005.1	RPS10	40S ribosomal protein S10	0.94	1.12	1.86	1.17	1.69	3.50	0.43	1.75	0.40			
NP_005372.2	NCL	nucleolin	1.11	1.10	1.86	2.53	2.04		1.05	1.12	1.06	1.09	1.19	2.93
NP_060979.2	LRRC59	leucine-rich repeat-containing protein 59	0.79	1.06	1.86								0.89	
NP_003503.1	HIST1H2AC	histone H2A type 1-C	0.99	0.85	1.85									
NP_000961.2	RPL6	60S ribosomal protein L6	0.94	1.25	1.84				1.01	1.25	1.26	0.36	0.89	1.32
NP_005331.1	HINT1	histidine triad nucleotide-binding protein 1	1.02	1.15	1.84				1.00	0.88	1.04			
NP_001186731.1	RPL11	60S ribosomal protein L11 isoform 2	0.93	1.13	1.84				0.93	1.18	1.09	0.45	0.78	1.54
NP_001011.1	RPS16	40S ribosomal protein S16	0.96	1.12	1.84				0.93	1.58	1.02	0.49	1.15	1.73
NP_004035.2	ATIC	bifunctional purine biosynthesis protein PURH	0.79	1.03	1.84	1.12	1.72	4.09					1.55	

NP_003893.2	FUBP1	far upstream element-binding protein 1	0.74	1.01	1.83	1.45	1.46							
NP_006079.1	TUBB2C	tubulin beta-2C chain	1.20	1.15	1.83	1.32	1.42	3.03	1.04	0.92	0.86			
NP_031401.1	TARDBP	TAR DNA-binding protein 43	1.13	1.14	1.82									
NP_001748.1	CBR1	carbonyl reductase [NADPH] 1	1.01	1.44	1.81	1.15	2.20	3.67	0.77	1.15				
NP_056229.2	RPL36	60S ribosomal protein L36	0.90	1.04	1.80				0.74	1.26	0.88			
NP_001013.1	RPS19	40S ribosomal protein S19	0.92	1.24	1.80				0.89	1.25	1.15	0.36	0.82	1.29
NP_003366.2	VDAC2	voltage-dependent anion-selective channel protein 2 isoform 2	0.66	0.73	1.79				0.66	0.90	1.03	0.42	0.87	1.84
NP_996756.1	PPP1CA	serine/threonine-protein phosphatase PP1-alpha catalytic subunit isoform 2	1.03	0.71	1.79									
NP_060140.2	OTUB1	ubiquitin thioesterase OTUB1	0.87	0.98	1.79	0.97	1.31	2.86	1.15					
NP_001143.2	SLC25A5	ADP/ATP translocase 2	0.93	0.75	1.78				0.80	0.92	0.66	0.72	1.17	1.89
NP_000962.2	RPL7	60S ribosomal protein L7	0.88	1.17	1.78	1.02	1.53	2.56	1.06	1.12	1.22	0.44	0.78	1.51
NP_002464.1	MYH9	myosin-9	0.65	0.64	1.78	0.71	0.93	2.96	0.65	0.63	1.01	0.35	0.93	2.01
NP_001153145.1	SYNCRIP	heterogeneous nuclear ribonucleoprotein Q isoform 2	0.86	1.10	1.77	1.31	1.81		0.77	0.89	0.96			
NP_001136077.1	EFTUD2	116 kDa U5 small nuclear ribonucleoprotein component isoform b	0.81	1.05	1.77									
NP_002806.2	PSMD11	26S proteasome non-ATPase regulatory subunit 11	0.84	1.12	1.77									
NP_001182375.1	SRSF7	serine/arginine-rich splicing factor 7 isoform 2	0.99	0.98	1.76				0.96	1.04	0.77			
NP_003391.1	XPO1	exportin-1	0.92	1.16	1.75	1.23	1.60	2.42	0.85	0.96	11.01			
NP_004981.2	MARS	methionyl-tRNA synthetase, cytoplasmic	1.06	1.48	1.75	1.15	1.74							
NP_001071634.1	SRSF1	serine/arginine-rich splicing factor 1 isoform 2	0.96	1.01	1.75							0.81	1.26	
NP_001627.2	SLC25A6	ADP/ATP translocase 3	0.49	0.62	1.75									
NP_001000.2	RPS5	40S ribosomal protein S5	0.99	1.26	1.75	1.12	1.64	2.93	1.05	1.30	1.23	0.43	0.88	8.77
NP_005795.2	DDX39A	ATP-dependent RNA helicase DDX39A	1.24	1.33	1.73									
NP_001127836.1	MAP4	microtubule-associated protein 4 isoform 4	0.64	0.78	1.73	0.52	1.22	2.27						
NP_006187.2	PCBP1	poly(rC)-binding protein 1	0.82	1.20	1.72									
NP_000970.1	RPL18	60S ribosomal protein L18	0.91	1.29	1.70				1.08	1.25	1.14	0.44	0.74	1.40
NP_001460.1	XRCC6	X-ray repair cross-complementing protein 6	0.96	1.02	1.70	2.14	2.34	3.06	0.77	0.93	0.84			
NP_954659.1	MATR3	matrin-3 isoform a	1.01	1.05	1.70				1.27	1.15	1.18			
NP_003133.1	SSB	lupus La protein	0.86	1.05	1.69									
NP_002630.2	SERPINB5	serpin B5	0.55	0.29	1.69	0.73	0.52	40.56	0.39	0.22	0.67			
NP_002563.1	PAFAH1B2	platelet-activating factor acetylhydrolase IB subunit beta isoform a	0.79	0.85	1.69	0.86	1.32	2.95						
NP_001279.2	CLIC1	chloride intracellular channel protein 1	0.70	0.83	1.68	0.81	1.22	2.91	0.57	0.68	0.83	0.43	1.01	2.47
NP_057156.1	GOLT1B	vesicle transport protein GOT1B	0.84	1.62	1.67									
NP_006280.3	TLN1	talin-1	0.91	1.64	1.67	1.05	2.42	3.42				0.38	2.04	2.04
NP_000978.1	RPL26	60S ribosomal protein L26	0.82	1.10	1.67				1.00	1.32	1.01			
NP_005338.1	HSPA5	78 kDa glucose-regulated protein precursor	1.19	1.36	1.66	0.66	1.09	1.09	1.48	1.82	1.25	0.51	0.83	1.12
NP_006358.1	CAP1	adenylyl cyclase-associated protein 1	0.67	0.61	1.66	0.68	0.83	2.89				0.36	0.81	3.20
NP_054733.2	SNRNP200	U5 small nuclear ribonucleoprotein 200 kDa helicase	1.08	1.27	1.66									
NP_005337.2	HSPA1B	heat shock 70 kDa protein 1A/1B	0.51	0.85	1.66	0.55	1.21	3.29	0.44	0.73	0.74		0.87	
NP_003123.2	SRM	spermidine synthase	1.08	1.09	1.65	1.41	2.07	3.04						
NP_001129487.1	ANXA2	annexin A2 isoform 2	0.63	0.45	1.65	0.65	0.65	2.49	0.64	0.45	0.77	0.50	0.72	2.46
NP_001427.2	FBL	rRNA 2'-O-methyltransferase fibrillarin	0.84	0.95	1.65									
NP_001006.1	RPS11	40S ribosomal protein S11	0.79	1.18	1.65				0.86	1.21	1.17	0.42	0.95	1.27
NP_001007.2	RPS12	40S ribosomal protein S12	0.78	1.13	1.64									
NP_001159831.1	SHMT2	serine hydroxymethyltransferase, mitochondrial isoform 3	0.82	1.27	1.64				0.92	1.50	1.29	0.10	1.82	0.34
NP_006436.3	PRPF8	pre-mRNA-processing-splicing factor 8	0.80	1.11	1.64									

NP_001005335.1	HNRNPL	heterogeneous nuclear ribonucleoprotein L isoform b	1.04	0.89	1.64									
NP_003339.1	UBE2N	ubiquitin-conjugating enzyme E2 N	0.80	1.25	1.64				0.70	1.02	0.85			
NP_061819.2	NANS	sialic acid synthase	0.82	0.87	1.64	1.06	1.46							
NP_002130.2	RBMX	heterogeneous nuclear ribonucleoprotein G isoform 1	0.77	0.89	1.63				0.98	1.24	0.99			
NP_001030611.1	FAM129B	niban-like protein 1 isoform 2	1.10	0.87	1.63	1.13	1.33	2.64	1.40	1.08	1.23	0.86	1.68	4.24
NP_001036816.1	TPM3	tropomyosin alpha-3 chain isoform 4	0.61	0.52	1.62	0.58	0.73		0.57	0.49	0.68			
NP_004492.2	HNRNPU	heterogeneous nuclear ribonucleoprotein U isoform b	0.97	1.07	1.62				1.13	1.23	1.04	0.86	0.91	2.23
NP_057215.3	RAB10	ras-related protein Rab-10	0.73	0.84	1.62	0.64	1.03	2.68				0.44	1.55	
NP_006752.1	YWHAE	14-3-3 protein epsilon	0.79	0.85	1.61	0.86	1.12	2.38	0.78	0.77	0.90	0.52	1.00	2.07
NP_006316.1	RAN	GTP-binding nuclear protein Ran	0.81	1.44	1.60	1.45	2.73	4.38	0.79	1.21	0.94	0.57	1.17	1.61
NP_003008.1	SRSF3	serine/arginine-rich splicing factor 3	0.99	1.02	1.59	1.12	1.95		0.95	1.05	0.82			
NP_001419.1	ENO1	alpha-enolase isoform 1	0.73	1.11	1.59	0.89	1.67	2.79	0.68	0.95	0.79	0.43	1.10	1.46
NP_000973.2	RPL21	60S ribosomal protein L21	0.91	1.16	1.59	1.20	1.68	2.55	0.95	1.19	1.13	0.38	0.72	1.05
NP_001070911.1	HNRNPC	heterogeneous nuclear ribonucleoproteins C1/C2 isoform b	0.80	1.24	1.58									
NP_002070.1	GOT1	aspartate aminotransferase, cytoplasmic	1.03	1.09	1.57		2.35	0.00						
NP_005310.1	HIST1H1C	histone H1.2	0.56	0.92	1.57				0.71	0.90	1.23	0.63	1.09	1.20
NP_001198.2	BTF3	transcription factor BTF3 isoform B	0.64	2.18	1.57				1.05	1.60	1.44			
NP_001138882.1	NONO	non-POU domain-containing octamer-binding protein isoform 2	0.78	1.02	1.57									
NP_002873.1	RANBP1	ran-specific GTPase-activating protein	0.71	0.71	1.56									
NP_003472.2	USP5	ubiquitin carboxyl-terminal hydrolase 5 isoform 2	0.64	1.36	1.56				1.11	2.97				
NP_000843.1	GSTP1	glutathione S-transferase P	0.71	0.80	1.56	0.59		1.91	0.69	0.64	0.74	0.44	0.81	1.17
NP_001116293.1	SET	protein SET isoform 1	0.91	0.47	1.55				1.09	0.80				
NP_001171691.1	CLTA	clathrin light chain A isoform f	0.46	6.68	1.55									
NP_703194.1	ILF3	interleukin enhancer-binding factor 3 isoform c	0.85	1.16	1.55				1.01	1.28	1.02			
NP_003396.1	YWHAH	14-3-3 protein eta	0.57	0.49	1.55	0.61	0.58	2.76	0.54	0.44	0.74			
NP_036220.1	PGLS	6-phosphogluconolactonase	0.57	0.57	1.55	0.74	0.79	2.16						
NP_060918.2	CAND1	cullin-associated NEDD8-dissociated protein 1	0.83	1.35	1.54	0.94	2.14	2.42	0.95	1.57				
XP_003119578.1	LOC100507855	adenylate kinase isoenzyme 4, mitochondrial-like	0.89	0.60	1.54									
NP_001348.2	DHX9	ATP-dependent RNA helicase A	0.97	0.98	1.52				1.07	0.97	0.90			
NP_001410.2	ELAVL1	ELAV-like protein 1	0.86	1.09	1.52				0.55	0.77	0.64			
NP_055205.2	SND1	staphylococcal nuclease domain-containing protein 1	0.87	1.25	1.52				0.79	1.18	0.67	0.46	1.01	1.33
NP_004960.2	IDE	insulin-degrading enzyme isoform 1 precursor	0.80	1.30	1.51									
NP_001349.2	DHX15	putative pre-mRNA-splicing factor ATP-dependent RNA helicase DHX15	0.92	1.02	1.50	2.14	2.41		1.26	0.95	1.62			
NP_000174.1	HADHB	trifunctional enzyme subunit beta, mitochondrial precursor	0.79	0.72	1.49				0.78	0.88	0.83			
NP_004125.3	HSPA9	stress-70 protein, mitochondrial precursor	1.16	1.23	1.49	1.01	1.19	1.80	1.18	1.30	0.91	0.70	1.27	1.60
NP_038470.1	STOML2	stomatin-like protein 2	1.11	1.06	1.49							0.64	1.09	1.42
NP_004574.2	RAB5C	ras-related protein Rab-5C isoform b	0.83	1.19	1.48				0.91	1.00		0.74	1.63	
NP_056461.1	RAP1B	ras-related protein Rap-1b precursor	0.70	1.34	1.48									
NP_002625.1	PHB	prohibitin	0.90	1.15	1.47				0.82	1.18	0.76	0.52	1.12	1.45
NP_001116847.1	HIST2H3D	histone H3.2	0.81	0.73	1.47				0.80	0.82	0.85	0.68	0.71	1.20
NP_114368.1	PTBP1	polypyrimidine tract-binding protein 1 isoform c	0.98	0.91	1.47				0.92	0.83	0.70			
NP_073591.2	SFXN1	sideroflexin-1	1.01	1.10	1.46				1.14	1.38	0.90			
NP_002620.1	PGAM1	phosphoglycerate mutase 1	0.59	0.87	1.46	0.78	1.42	2.60	0.61	0.73	0.83	0.44	0.89	1.45
NP_733821.1	LMNA	prelamin-A/C isoform 1 precursor	0.67	0.59	1.46									
NP_001186128.1	AK2	adenylate kinase 2, mitochondrial isoform c	0.83	0.71	1.45				0.83	0.83	0.77	0.52	0.75	2.04

NP_005753.1	TRIM28	transcription intermediary factor 1-beta	1.00	1.40	1.44				1.33	1.93	1.19			
NP_005733.1	PDIA6	protein disulfide-isomerase A6 precursor	0.73	1.14	1.43							0.30	0.59	0.80
NP_112480.2	HNRNPM	heterogeneous nuclear ribonucleoprotein M isoform b	0.93	0.94	1.42				1.15	1.16	1.00			
NP_004896.1	PRDX6	peroxiredoxin-6	0.93	1.05	1.42	1.00	1.46	2.32	0.74	0.89	0.56	0.50	0.86	
NP_001156758.1	EWSR1	RNA-binding protein EWS isoform 4	0.76	0.89	1.42	1.69	1.94		0.74	0.84	0.81			
NP_000173.2	HADHA	trifunctional enzyme subunit alpha, mitochondrial precursor	0.65	0.72	1.41							0.50	0.85	1.55
NP_005947.3	MTHFD1	C-1-tetrahydrofolate synthase, cytoplasmic	0.73	0.75	1.41	0.82	0.95	2.42	0.67	0.76	0.74			
NP_004850.1	CLTC	clathrin heavy chain 1	0.71	0.88	1.40	0.85	1.46	2.67	0.69	0.93	0.81	0.56	1.32	2.55
NP_116120.1	TXNDC17	thioredoxin domain-containing protein 17	1.06	0.80	1.40									
NP_001070674.1	SEC31A	protein transport protein Sec31A isoform 4	0.76	1.05	1.39									
NP_113584.3	HUWE1	E3 ubiquitin-protein ligase HUWE1	0.93	0.70	1.39	1.01	0.91	2.34						
NP_001138303.1	PHB2	prohibitin-2 isoform 1	0.90	1.15	1.39				0.88	1.32	0.77	0.53	1.14	1.32
NP_003555.1	TAGLN2	transgelin-2	0.52	0.63	1.38	0.57	0.87	2.36	0.42	0.51	0.59	0.31	0.85	1.87
NP_005773.3	THOC4	THO complex subunit 4	0.96	1.05	1.38				1.19	1.22	0.97			
NP_001596.2	AARS	alanyl-tRNA synthetase, cytoplasmic	0.46	0.84	1.37	0.52	1.11		0.49	0.69				
NP_001273.1	AP2B1	AP-2 complex subunit beta isoform b	0.79	1.03	1.36							0.83	2.03	2.81
NP_066299.2	MYL6	myosin light polypeptide 6 isoform 1	0.63	0.57	1.36	0.58	0.73	2.12	0.67	0.54	0.80	0.38	0.98	1.43
NP_006704.3	SUB1	activated RNA polymerase II transcriptional coactivator p15	0.83	0.92	1.35				0.76	1.08	0.79			
NP_002256.2	KPNB1	importin subunit beta-1	0.79	0.97	1.35	0.92	1.35	2.62	0.92	0.95	0.85	0.67	0.98	1.62
NP_002939.2	RPL15	60S ribosomal protein L15	0.67	1.08	1.35				0.84	1.23	1.04	0.44	0.87	1.59
NP_068831.1	JUP	junction plakoglobin	0.50	0.10	1.35				0.09	0.02	0.11	0.27	0.32	3.31
NP_036558.3	SF3B3	splicing factor 3B subunit 3	0.78	1.15	1.34									
NP_001144.1	ANXA4	annexin A4	0.59	0.35	1.34	0.78	0.64	2.41		0.42		0.59	0.59	1.52
NP_112533.1	HNRNPA2B1	heterogeneous nuclear ribonucleoproteins A2/B1 isoform B1	0.92	0.87	1.34				0.92	0.91				
NP_001782.1	CDC42	cell division control protein 42 homolog isoform 1	0.61	0.55	1.33	0.68	0.70	2.43	0.55	0.44	0.43	0.37	1.08	0.69
NP_001122326.1	SRRT	serrate RNA effector molecule homolog isoform e	0.74	1.35	1.32									
NP_821133.1	TUBB	tubulin beta chain	0.56	0.64	1.32	0.67	0.97	2.75	0.58	0.60	0.82	0.61	0.77	2.46
NP_002119.1	HMGB1	high mobility group protein B1	0.76	0.83	1.32	0.94	1.29		0.69	0.79	0.86	0.69	0.92	1.90
NP_005057.1	SFPQ	splicing factor, proline- and glutamine-rich	0.97	1.14	1.32				0.96	1.25	0.79			
NP_001001329.1	PRKCSH	glucosidase 2 subunit beta isoform 2	0.34	0.88	1.31				0.45	0.95	0.54			
NP_620119.2	BAX	apoptosis regulator BAX isoform sigma	0.70	0.91	1.30	1.02	1.41	2.77	0.55	0.80		0.58	1.25	
NP_006462.1	MYL12A	myosin regulatory light chain 12A	0.55	0.67	1.30				0.74	0.59				
NP_002147.2	HSPD1	60 kDa heat shock protein, mitochondrial	0.88	1.02	1.29	0.75	1.07	1.78	0.90	1.14	0.76	0.47	0.97	1.09
NP_066964.1	XRCC5	X-ray repair cross-complementing protein 5	0.79	0.96	1.28	1.30	1.84		0.99	0.89	0.94			
NP_570603.2	AP2A1	AP-2 complex subunit alpha-1 isoform 2	0.80	1.18	1.28									
NP_005713.1	ACTR2	actin-related protein 2 isoform b	0.64	0.75	1.28									
NP_037506.2	PDCD6IP	programmed cell death 6-interacting protein isoform 1	0.54	0.55	1.27	0.61	0.65	2.56	0.49	0.43	0.61			
NP_001924.2	DLST	dihydrolipoyllysine-residue succinyltransferase component of 2-oxoglutarate dehydrogenase complex, mitochondrial	0.70		1.25				0.54	0.73		0.21	0.29	0.56
NP_004777.1	TXNL1	thioredoxin-like protein 1	0.76	1.27	1.25									
NP_061828.1	UBB	polyubiquitin-B precursor	0.76	0.71	1.24				0.67	0.72	0.85	0.59	1.43	3.68
NP_003676.2	KHSRP	far upstream element-binding protein 2	0.87	1.07	1.24									
NP_000994.1	RPLP1	60S acidic ribosomal protein P1 isoform 1	0.64	1.29	1.24	1.11	1.46	2.83	0.94	1.07	1.07	0.39	0.79	1.17
NP_001159491.1	AP1B1	AP-1 complex subunit beta-1 isoform c	0.57	0.58	1.23				0.56	1.74		0.42	0.73	1.70

NP_001611.1	AHNAK	neuroblast differentiation-associated protein AHNAK isoform 1	0.70	0.47	1.23	0.80	0.82	5.26	0.69	0.47	0.56	0.52	0.87	3.90
NP_005013.1	PFN1	profilin-1	0.61	0.64	1.23	0.84	0.91	2.71	0.60	0.63	0.70	0.03	0.03	
NP_008937.1	NUDT21	cleavage and polyadenylation specificity factor subunit 5	0.75	0.91	1.22									
NP_003134.1	SSBP1	single-stranded DNA-binding protein, mitochondrial precursor	0.80	0.82	1.22				0.87	0.89	0.72	0.55	0.99	
NP_647539.1	YWHAB	14-3-3 protein beta/alpha	0.74	0.61	1.21	0.72	0.83	1.90	0.64	0.61	0.57	0.58	0.89	2.25
NP_066953.1	PPIA	peptidyl-prolyl cis-trans isomerase A	0.64	0.73	1.21	1.00	1.77	2.66	0.59	0.62	0.56	0.40	0.69	1.42
NP_066544.1	HIST1H2AJ	histone cluster 1, H2aj	0.66	0.68	1.21	0.54	0.60	1.06	0.77	0.74	0.85	0.65	0.69	1.25
NP_004784.2	LONP1	lon protease homolog, mitochondrial precursor	0.82	0.84	1.20				1.23	1.42				
NP_002564.1	PAFAH1B3	platelet-activating factor acetylhydrolase IB subunit gamma	0.92	0.78	1.19	1.27	1.29							
NP_067676.2	HNRNPH3	heterogeneous nuclear ribonucleoprotein H3 isoform b	0.88	0.82	1.19									
NP_006808.1	ERP29	endoplasmic reticulum resident protein 29 isoform 1 precursor	0.49	1.13	1.19				0.49	1.23		0.10	0.19	0.29
NP_064505.1	UGGT1	UDP-glucose:glycoprotein glucosyltransferase 1 precursor	0.56	1.01	1.18									
NP_001186041.1	MDH1	malate dehydrogenase, cytoplasmic isoform 3	0.74	0.91	1.18	0.98	1.35	2.43	0.71	0.85	0.51	0.52	0.92	1.35
NP_001737.1	CANX	calnexin precursor	0.67	1.19	1.18				0.67	1.22	0.65	0.23	0.76	0.63
NP_003861.1	IQGAP1	ras GTPase-activating-like protein IQGAP1	0.75	0.41	1.18	0.68	0.51	2.31	0.76	0.33	0.60	0.45	0.91	1.78
NP_064601.3	RNPEP	aminopeptidase B	0.60	0.46	1.18									
NP_001145.1	ANXA5	annexin A5	1.03	1.16	1.18	1.30	1.73	2.33	1.03	1.10	0.62	0.62	1.15	0.81
NP_003325.2	UBA1	ubiquitin-like modifier-activating enzyme 1	0.87	0.79	1.17	1.16	1.21	2.27	0.82	0.71	0.58	0.55	0.71	0.87
NP_001509.3	GTF2I	general transcription factor II-I isoform 4	0.79	0.94	1.17				0.01	0.01				
NP_944490.1	SRI	sorcin isoform b	0.50	0.54	1.16				0.43	0.46				
NP_775083.1	CAST	calpastatin isoform b	0.37	0.60	1.16	0.46	0.95	3.03						
NP_006380.1	HYOU1	hypoxia up-regulated protein 1 precursor	0.94	1.76	1.16	0.56	1.27	1.28						
NP_057310.1	VPS29	vacuolar protein sorting-associated protein 29 isoform 1	0.59	0.67	1.16									
NP_001119523.1	HDGF	hepatoma-derived growth factor isoform c	0.58	0.76	1.16	0.60	1.06							
NP_001894.2	CTNNA1	catenin alpha-1	0.76	0.34	1.16	0.86	0.53	9.00	0.85	0.47	0.56	0.72	0.93	3.16
NP_001738.2	CAPG	macrophage-capping protein	0.71	0.27	1.16	1.07	0.85	3.34				0.58	0.31	1.19
NP_004334.1	CALR	calreticulin precursor	0.42	0.87	1.15							0.04	0.04	0.14
NP_004921.1	CAPZB	F-actin-capping protein subunit beta isoform 1	0.70	1.14	1.15							0.46	1.09	2.29
NP_001093640.1	IMMT	mitochondrial inner membrane protein isoform 3	0.73	0.78	1.15				0.71	0.97	0.60			
NP_001121134.1	GSN	gelsolin isoform b	0.46	0.20	1.14	0.58	0.25	2.42	0.55	0.17	0.61	0.15	0.11	0.62
NP_001166.3	ARHGDIB	rho GDP-dissociation inhibitor 2	1.17	0.52	1.14									
NP_001078936.1	CTNND1	catenin delta-1 isoform 3A	0.75	0.30	1.13				0.97	0.38	0.69	0.75	0.80	3.21
NP_001895.1	CTNNB1	catenin beta-1	0.81	0.36	1.13									
NP_002037.2	GAPDH	glyceraldehyde-3-phosphate dehydrogenase	0.56	1.26	1.13	0.65	1.82	2.29	0.55	1.07	0.62	0.37	1.20	1.25
NP_001011553.2	SEPT7	septin-7 isoform 2	0.53	0.61	1.12								1.11	
NP_000933.1	PPIB	peptidyl-prolyl cis-trans isomerase B precursor	0.70	1.17	1.12				0.66	1.27	0.59	0.30	0.70	0.73
NP_001132913.1	BCAP31	B-cell receptor-associated protein 31 isoform b	0.65	0.91	1.11							0.28	0.59	0.87
NP_005887.2	IDH1	isocitrate dehydrogenase [NADP] cytoplasmic	0.55	0.59	1.11	0.53	0.60	1.35	0.55	0.57	0.51			
NP_057226.1	HSD17B12	estradiol 17-beta-dehydrogenase 12	0.19	0.32	1.11				0.22	0.45			0.33	
NP_002097.1	H2AFZ	histone H2A.Z	0.49	0.60	1.10				0.51	0.60	0.61			
NP_005304.3	PDIA3	protein disulfide-isomerase A3 precursor	0.70	0.89	1.10	0.46	0.92	1.00	0.73	1.02	0.62	0.25	0.49	0.57
NP_003536.1	HIST1H4E	histone H4	0.66	0.57	1.10				0.67	0.69	0.66	0.54	0.54	0.71

NP_938148.1	GANAB	neutral alpha-glucosidase AB isoform 2	0.73	1.05	1.09				0.66	1.08	0.54	0.33	0.58	0.78
NP_940684.1	PTPLB	3-hydroxyacyl-CoA dehydratase 2	0.39	0.43	1.09				0.12	0.13	0.16	0.16	0.34	0.82
NP_001092.1	ACTB	actin, cytoplasmic 1	0.81	0.84	1.09	0.92	1.25	2.90	0.81	0.77	0.95	0.56	1.70	2.55
NP_000282.1	PGK1	phosphoglycerate kinase 1	0.52	0.69	1.08	0.64	0.99	2.29	0.44	0.57	0.47	0.32	0.62	0.92
NP_003290.1	HSP90B1	endoplasmin precursor	0.72	1.95	1.08	0.52	1.89		0.77	2.12	0.68	0.29	1.07	0.66
NP_000356.1	TPI1	triosephosphate isomerase isoform 1	0.60	1.23	1.07	0.77	1.88	1.97	0.56	1.02	0.57	0.35	1.26	0.80
NP_998776.1	SLC25A3	phosphate carrier protein, mitochondrial isoform b precursor	0.69	1.05	1.07				0.55	1.23	0.51	0.56	1.54	1.79
NP_002406.1	MIF	macrophage migration inhibitory factor	0.56	0.78	1.07				0.72	0.66				
NP_036226.1	PRDX5	peroxiredoxin-5, mitochondrial isoform a precursor	0.46	0.61	1.07	0.57	0.78	2.44						
NP_003960.1	UBE2M	NEDD8-conjugating enzyme Ubc12	0.81	1.39	1.07				0.94	1.23				
NP_001008491.1	SEPT2	septin-2	0.57	0.72	1.07				0.70	0.42	0.71	0.20	0.71	0.70
NP_579899.1	MYOF	myoferlin isoform b	0.36	0.40	1.06							0.51	1.10	3.08
NP_006746.1	TALDO1	transaldolase	0.46	0.70	1.06	0.65	1.13	2.23	0.38	0.53	0.45	0.43	0.77	
NP_001013269.1	SLC3A2	4F2 cell-surface antigen heavy chain isoform f	0.90	0.38	1.05	0.90	0.99	4.65	1.06	0.39	0.67	1.04	1.46	4.82
NP_859048.1	PRDX1	peroxiredoxin-1	0.63	0.92	1.05				0.63	0.78	0.58	0.37	0.87	1.17
NP_004068.2	CS	citrate synthase, mitochondrial precursor	0.59	1.31	1.04	0.17	0.86		0.63	1.27	0.60	0.39	1.27	1.30
NP_001976.1	ETFB	electron transfer flavoprotein subunit beta isoform 1	0.87	0.64	1.04				0.73	0.70	0.41	0.34	0.68	0.69
NP_002941.1	RPN1	dolichyl-diphosphooligosaccharide--protein glycosyltransferase subunit 1 precursor	0.69	1.13	1.04				0.89	1.39	1.18	0.37	0.85	1.08
NP_003115.1	SPR	sepiapterin reductase	0.89	1.01	1.03									
NP_000025.1	ALDOA	fructose-bisphosphate aldolase A isoform 1	0.40	0.66	1.03	0.46	0.91	1.58	0.38	0.60	0.51	0.22	0.63	0.97
NP_005909.2	MDH2	malate dehydrogenase, mitochondrial precursor	0.75	0.69	1.02	0.57	0.41	1.18	0.79	0.79	0.55	0.42	0.75	1.17
NP_005498.1	CFL1	cofilin-1	0.49	0.65	1.02	0.63	0.82	2.23	0.46	0.53	0.49	0.36	1.71	3.73
NP_000134.2	FH	fumarate hydratase, mitochondrial precursor	0.66	0.72	1.00				0.73	0.84	0.51	0.32	0.62	0.93
NP_277035.2	HK1	hexokinase-1 isoform HKI-td	0.64	1.01	0.98				0.67	0.95	0.63			
NP_663723.1	YWHAZ	14-3-3 protein zeta/delta	0.44	0.38	0.98	0.47	0.49	1.65	0.44	0.34	0.52	0.30	0.47	1.36
NP_005557.1	LDHA	L-lactate dehydrogenase A chain isoform 1	0.52	0.63	0.97	0.60	0.87	1.72	0.48	0.55	0.47	0.31	0.59	0.85
NP_054817.2	PRDX3	thioredoxin-dependent peroxide reductase, mitochondrial isoform b	0.69	0.69	0.97	0.58	0.70	1.62	0.82	0.81	0.60	0.46	0.86	
NP_005800.3	PRDX2	peroxiredoxin-2 isoform a	0.66	0.91	0.97	0.92	1.33	2.06	0.76	0.90	0.65	0.38	0.89	0.92
NP_005511.1	HNRNPH1	heterogeneous nuclear ribonucleoprotein H	0.60	1.09	0.97				0.88	1.01	0.78			
NP_149073.1	SARNP	SAP domain-containing ribonucleoprotein	0.70	1.06	0.96									
NP_008839.2	RAC1	ras-related C3 botulinum toxin substrate 1 isoform Rac1	0.53	0.66	0.95				0.61	0.64	0.65	0.69	1.60	3.43
NP_001121188.1	ETFA	electron transfer flavoprotein subunit alpha, mitochondrial isoform b	0.81	0.51	0.94				0.83	0.58	0.39	0.32	0.51	0.74
NP_000010.1	ACAT1	acetyl-CoA acetyltransferase, mitochondrial precursor	0.79	0.55	0.91									
NP_002853.2	PYGB	glycogen phosphorylase, brain form	0.48	0.41	0.91	0.51	0.53	1.53	0.46	0.37	0.46	0.22	0.32	0.41
NP_112243.1	RAB1B	ras-related protein Rab-1B	0.43	0.51	0.90	0.63	1.00	2.16	0.42	0.51	0.39	0.11	0.71	6.91
NP_003365.1	VDAC1	voltage-dependent anion-selective channel protein 1	0.58	0.56	0.90				0.61	0.63	0.55	0.31	0.55	0.75
NP_002558.1	PEBP1	phosphatidylethanolamine-binding protein 1 preproprotein	0.37	0.96	0.90				0.33	0.61	0.49			
NP_002379.2	MCM3	DNA replication licensing factor MCM3	0.64	0.79	0.89									
NP_001760.1	CD9	CD9 antigen	0.45	0.32	0.89				0.53	0.33		0.26	0.52	
NP_057417.3	SRRM2	serine/arginine repetitive matrix protein 2	0.58	0.54	0.88									
NP_004902.1	PDIA4	protein disulfide-isomerase A4 precursor	0.57	0.75	0.88									
NP_057152.2	FIS1	mitochondrial fission 1 protein	0.49	0.54	0.88									
NP_001001937.1	ATP5A1	ATP synthase subunit alpha, mitochondrial precursor	0.68	0.79	0.88				0.70	0.89	0.48	0.41	0.83	0.81
NP_056107.1	ESYT1	extended synaptotagmin-1 isoform 2	0.61	0.99	0.87							0.22	0.97	

NP_001089.1	ACO2	aconitate hydratase, mitochondrial precursor	0.67	0.83	0.87				0.80	0.91	0.45	0.49	1.04	0.94
NP_001003962.1	CAPNS1	calpain small subunit 1	0.68	0.43	0.87	0.78	0.61	1.91	0.67	0.39	0.44	0.48	0.44	0.90
NP_004083.3	ECHS1	enoyl-CoA hydratase, mitochondrial precursor	0.49	0.58	0.86				0.16	0.18		0.21	0.67	
NP_004539.1	NDUFB10	NADH dehydrogenase [ubiquinone] 1 beta subcomplex subunit 10	0.63	0.66	0.86				0.53	0.58		0.46	1.05	
NP_055112.2	ETHE1	protein ETHE1, mitochondrial precursor	0.74	0.41	0.86				0.75	0.31				
NP_001034455.1	ATP6V1E1	V-type proton ATPase subunit E 1 isoform b	0.31	0.23	0.86	0.40	0.48	1.16	0.37	0.39				
NP_005991.1	TUBA4A	tubulin alpha-4A chain	0.37		0.85	0.71	1.00	2.79	0.37		0.31	0.32	0.86	1.74
NP_000476.1	APRT	adenine phosphoribosyltransferase isoform a	0.42	0.43	0.84	0.53	0.62	1.66	0.27	0.24	0.30	0.31	0.41	
NP_001186221.1	HSD17B4	peroxisomal multifunctional enzyme type 2 isoform 3	0.76	1.02	0.84									
NP_076997.1	TMEM109	transmembrane protein 109 precursor	0.61	0.85	0.83				0.52	0.96		0.40	0.57	
NP_001677.2	ATP5B	ATP synthase subunit beta, mitochondrial precursor	0.67	0.83	0.83	0.44	0.62	0.62	0.71	0.88	0.48	0.40	0.80	0.82
NP_000909.2	P4HB	protein disulfide-isomerase precursor	0.52	1.15	0.83							0.28	0.02	0.65
NP_066997.3	KIAA1967	p30 DBC protein	0.46	0.45	0.82				0.38	0.57	0.32			
NP_006102.2	ACAA2	3-ketoacyl-CoA thiolase, mitochondrial	0.61	0.48	0.82	0.50	0.46		0.62	0.57	0.47	0.32	0.63	0.72
NP_001389.2	ECH1	delta(3,5)-Delta(2,4)-dienoyl-CoA isomerase, mitochondrial precursor	0.53	0.46	0.81				0.53	0.56	0.41	0.29	0.50	0.93
NP_003370.2	EZR	ezrin	0.45	0.27	0.79	0.60	0.37	1.98	0.37	0.29	0.36	0.24	0.56	1.54
NP_005546.2	KRT6B	keratin, type II cytoskeletal 6B	0.44	0.02	0.79				73.22	1.00	140.61			
NP_001226.2	SERPINH1	serpin H1 precursor	1.26	11.98	0.78				1.95	10.10	0.68	0.09	3.79	0.10
NP_001182461.1	SPTAN1	spectrin alpha chain, brain isoform 3	0.56	0.60	0.78	0.06	1.74						1.41	
NP_059447.2	MVP	major vault protein	0.14	0.32	0.77	0.16	0.48		0.24	0.36		0.03	0.03	0.27
NP_000166.2	GPI	glucose-6-phosphate isomerase isoform 2	0.69	0.98	0.77	0.81	1.46	1.16	0.57	0.81	0.34	0.39	0.96	0.67
NP_002071.2	GOT2	aspartate aminotransferase, mitochondrial precursor	0.48	0.45	0.76				0.36	0.50	0.33		0.44	
NP_057049.5	NDUFA13	NADH dehydrogenase [ubiquinone] 1 alpha subcomplex subunit 13	0.52	0.63	0.76							0.56	0.91	1.10
NP_001688.1	ATP5O	ATP synthase subunit O, mitochondrial precursor	0.63	0.79	0.76				0.73	0.90	0.45	0.34	0.78	0.63
NP_001153706.1	ATP1A1	sodium/potassium-transporting ATPase subunit alpha-1 isoform d	0.62	0.49	0.75	0.39	0.75	1.50	0.65	0.54	0.35			
NP_116139.1	ABHD14B	abhydrolase domain-containing protein 14B	0.57	0.75	0.73									
NP_001177258.1	ATP5G3	ATP synthase lipid-binding protein, mitochondrial isoform B precursor	0.48	0.66	0.73				0.60	0.86	0.48			
NP_005660.4	REEP5	receptor expression-enhancing protein 5	0.35	0.42	0.73									
NP_005552.3	LAMP1	lysosome-associated membrane glycoprotein 1 precursor	0.41	0.47	0.72				0.53	0.49		0.45	1.22	1.35
NP_001003785.1	ATP5H	ATP synthase subunit d, mitochondrial isoform b	0.59	0.76	0.72				0.66	0.85	0.44	0.44	0.81	1.00
NP_064696.1	MGST1	microsomal glutathione S-transferase 1	0.30	0.91	0.70							0.26	0.74	
NP_001029031.1	ACADVL	very long-chain specific acyl-CoA dehydrogenase, mitochondrial isoform 2 precursor	0.31	0.39	0.69				0.49	0.25				
NP_003356.2	UQCRC1	cytochrome b-c1 complex subunit 1, mitochondrial precursor	0.55	0.57	0.69				0.77	0.58		0.25	0.62	0.54
NP_000445.1	SOD1	superoxide dismutase [Cu-Zn]	0.51	0.71	0.68				0.47	0.73	0.39			
NP_003312.3	TUFM	elongation factor Tu, mitochondrial precursor	0.45	0.52	0.65				0.32	0.73	0.24	0.29	0.66	0.71
NP_004246.2	COX5A	cytochrome c oxidase subunit 5A, mitochondrial precursor	0.50	0.51	0.65				0.43	0.53	0.32			
YP_003024029.1	COX2	cytochrome c oxidase subunit II	0.51	0.42	0.64				0.48	0.45	0.37	0.29	0.36	0.54
NP_001120695.1	CD59	CD59 glycoprotein preproprotein	0.24	0.36	0.63								1.34	
NP_003125.3	SRP14	signal recognition particle 14 kDa protein	0.78	1.09	0.63	1.05	2.00							
NP_006730.2	MCM5	DNA replication licensing factor MCM5	0.57	0.68	0.62									
NP_005316.1	HIST1H1A	histone H1.1	1.13	1.30	0.62									

NP_002645.3	PKM2	pyruvate kinase isozymes M1/M2 isoform a	0.48	0.62	0.61	0.52	0.84	1.15	0.49	0.55	0.33	0.28	0.54	0.52
NP_877423.1	MCM4	DNA replication licensing factor MCM4	0.48	0.79	0.60									
NP_001852.1	COX4I1	cytochrome c oxidase subunit 4 isoform 1, mitochondrial precursor	0.43	0.19	0.60									
NP_001907.2	CYC1	cytochrome c1, heme protein, mitochondrial	0.47	0.52	0.59				0.47	0.60		0.31	0.56	1.93
NP_004517.2	MCM2	DNA replication licensing factor MCM2	0.48	0.73	0.58									
NP_005653.3	VDAC3	voltage-dependent anion-selective channel protein 3 isoform b	0.37	0.81	0.57				0.44	0.54				
NP_006301.3	NPEPPS	puromycin-sensitive aminopeptidase	0.43	0.60	0.56	0.67	1.03	0.86				0.09	0.09	
NP_001744.2	CAV1	caveolin-1 isoform alpha	0.43	0.40	0.55				0.47	0.48		0.49	0.86	1.50
NP_002297.2	LGALS3	galectin-3 isoform 1	0.36	0.22	0.55	0.40	0.30	0.89	0.29	0.22		0.31	0.24	0.71
NP_000215.1	KRT18	keratin, type I cytoskeletal 18	0.52	0.35	0.54				0.50	0.39	0.77			
NP_005130.1	ANXA3	annexin A3	0.14	0.05	0.54	0.53	0.10	3.09	0.42	0.02	0.76	0.02	0.02	0.16
NP_001123291.1	CYB5R3	NADH-cytochrome b5 reductase 3 isoform 2	0.37	0.47	0.54				0.34	0.42				
NP_036450.1	LETM1	LETM1 and EF-hand domain-containing protein 1, mitochondrial precursor	0.36	0.38	0.53				0.64	0.75				
NP_003357.2	UQCRC2	cytochrome b-c1 complex subunit 2, mitochondrial precursor	0.45	0.62	0.52				0.50	0.66	0.43	0.26	0.56	0.64
NP_001124161.1	HMGB2	high mobility group protein B2	0.64	0.75	0.50							0.05	0.05	
NP_005906.2	MCM6	DNA replication licensing factor MCM6	0.46	0.94	0.44	9.96	11.28			0.38				
NP_000415.2	KRT5	keratin, type II cytoskeletal 5	0.20	0.17	0.44				0.22	0.18	0.29	0.17	0.26	1.01
NP_066270.1	CKMT1B	creatine kinase U-type, mitochondrial precursor	0.53	0.22	0.44				0.53	0.24	0.17	0.27	0.15	0.44
NP_001900.1	CTSD	cathepsin D preproprotein	0.28	0.59	0.44	0.04	0.04	0.12	0.35	0.65	0.27	0.36	1.82	0.65
NP_001531.1	HSPB1	heat shock protein beta-1	0.16	0.26	0.41	0.17	0.30	0.85	0.18	0.23	0.24	0.10	0.27	0.41
NP_060530.3	IARS2	isoleucyl-tRNA synthetase, mitochondrial precursor	0.24	0.25	0.40				0.72	0.43	0.52			
NP_005554.1	STMN1	stathmin isoform a	0.46	0.49	0.39				0.43	0.40	0.27			
NP_006818.3	TMED10	transmembrane emp24 domain-containing protein 10 precursor	0.25	0.33	0.39				0.29	0.35		0.05	0.05	
NP_004915.2	ACTN4	alpha-actinin-4	0.62	0.57	0.39	0.66	0.82	1.97	0.65	0.56	0.53	0.35	1.19	2.58
NP_071504.2	PC	pyruvate carboxylase, mitochondrial precursor	0.38	0.77	0.37	1.20	2.77		0.30	0.79	0.13	0.24	0.90	
NP_004869.1	PTGES	prostaglandin E synthase	1.10	0.28	0.25				1.32	0.33		0.48	0.22	
NP_000413.1	KRT17	keratin, type I cytoskeletal 17	0.23	0.12	0.22				0.25	0.13	0.22	0.25	0.16	1.04
NP_002023.2	FTH1	ferritin heavy chain	0.30	0.53	0.21	0.36	0.71		0.21	0.41		0.23	0.71	
NP_005547.3	KRT7	keratin, type II cytoskeletal 7	0.17	0.11	0.20				0.17	0.13	0.17	0.14	0.14	0.57
NP_002264.1	KRT8	keratin, type II cytoskeletal 8	0.11	0.09	0.19				0.11	0.11	0.21	0.09	0.06	0.66
NP_001141.2	ANPEP	aminopeptidase N precursor	0.47	0.59	0.15	0.41	1.01					0.36	1.13	0.17
NP_002266.2	KRT15	keratin, type I cytoskeletal 15	0.04	0.03	0.14				0.02	0.02	0.06	0.02	0.02	0.32
NP_005262.1	GLUD1	glutamate dehydrogenase 1, mitochondrial precursor	1.04	0.56	0.12				1.10	0.64		0.53	0.70	
NP_000058.1	CA2	carbonic anhydrase 2	0.01	0.03	0.05	0.03	0.05	0.12	0.03	0.03	0.05			
NP_001619.1	AKR1B1	aldose reductase	0.39	5.85		0.75	7.74	2.33				0.39	5.28	
NP_055841.2	MON2	protein MON2 homolog	0.71	3.99										
NP_001138616.1	SLC1A5	neutral amino acid transporter B(0) isoform 2	3.85	3.79										
NP_006816.2	CKAP4	cytoskeleton-associated protein 4	0.73	3.19										
NP_653333.1	HNRNPUL1	heterogeneous nuclear ribonucleoprotein U-like protein 1 isoform d	1.43	3.07										
NP_057368.3	CNOT1	CCR4-NOT transcription complex subunit 1 isoform a	1.06	2.54										
NP_000137.2	FTL	ferritin light chain		2.11		1.15	4.79							
NP_000926.2	PLOD2	procollagen-lysine,2-oxoglutarate 5-dioxygenase 2 isoform 2 precursor	0.12	2.05										
NP_079199.2	NUP210	nuclear pore membrane glycoprotein 210 precursor	0.99	2.03										

NP_009109.3	STRAP	serine-threonine kinase receptor-associated protein	0.81	1.76										
NP_001122386.1	PCBP2	poly(rC)-binding protein 2 isoform g	0.84	1.69										
NP_005040.2	PWP2	periodic tryptophan protein 2 homolog	2.04	1.69										
NP_995585.2	PARD3B	partitioning defective 3 homolog B isoform a	0.99	1.65										
NP_942127.1	ACLY	ATP-citrate synthase isoform 2	0.97	1.64		1.02	2.33		0.79	1.56		0.56	1.32	
NP_079172.2	ERMP1	endoplasmic reticulum metallopeptidase 1		1.51										
NP_001015054.1	MPG	DNA-3-methyladenine glycosylase isoform c	0.93	1.51										
NP_002863.1	RAC2	ras-related C3 botulinum toxin substrate 2 precursor		1.49					2.74	1.11				
NP_001139247.1	FKBP5	peptidyl-prolyl cis-trans isomerase FKBP5 isoform 1		1.45		0.53	1.46							
NP_008924.1	NPM3	nucleoplasmin-3		1.44										
NP_001019637.1	SOD2	superoxide dismutase [Mn], mitochondrial isoform B precursor	0.30	1.39			1.59							
NP_001157666.1	PLIN3	perilipin-3 isoform 3	1.39	1.38										
NP_003082.1	SNRPB	small nuclear ribonucleoprotein-associated proteins B and B' isoform B	0.78	1.35										
NP_001186709.1	PSMB2	proteasome subunit beta type-2 isoform 3	0.94	1.34					0.94	1.38	1.12			
NP_001303.1	CRIP2	cysteine-rich protein 2	0.17	1.33					0.19	1.17		0.04	2.09	
NP_001008800.1	CCT3	T-complex protein 1 subunit gamma isoform c	1.21	1.31								0.79	1.12	
NP_000041.2	ASS1	argininosuccinate synthase	0.43	1.29		0.59	1.51		0.37	1.41				
NP_005720.1	PPIF	peptidyl-prolyl cis-trans isomerase F, mitochondrial precursor	1.18	1.27					2.08	2.04		0.69	1.28	2.05
XP_001717770.2	LOC389217	protein SET-like	1.45	1.22										
NP_665906.1	HMGA1	high mobility group protein HMG-I/HMG-Y isoform a	1.35	1.19					1.40	1.02				
NP_001152763.1	MICAL1	NEDD9-interacting protein with calponin homology and LIM domains isoform 2	1.02	1.19										
NP_002780.1	PSMA4	proteasome subunit alpha type-4 isoform 1	1.06	1.18										
NP_001186702.1	PSMA5	proteasome subunit alpha type-5 isoform 2	0.91	1.18										
NP_003818.2	NAPA	alpha-soluble NSF attachment protein	0.61	1.17									1.06	
NP_694546.1	PSMC4	26S protease regulatory subunit 6B isoform 2	0.86	1.17										
NP_065123.1	EIF5A2	eukaryotic translation initiation factor 5A-2	0.91	1.16					0.91	1.07		0.36	0.79	
NP_115982.1	HINT2	histidine triad nucleotide-binding protein 2, mitochondrial precursor	1.10	1.15										
NP_003923.2	ST13	hsc70-interacting protein	0.83	1.15										
NP_001005409.1	SF3A1	splicing factor 3A subunit 1 isoform 2	0.97	1.13										
NP_001129609.1	RPL28	60S ribosomal protein L28 isoform 5	1.16	1.12										
NP_038461.2	TNPO2	transportin-2 isoform 2	0.97	1.10										
NP_112552.1	HNRNPK	heterogeneous nuclear ribonucleoprotein K isoform b	1.01	1.10		1.31	1.93		0.91	1.17	1.01			
NP_665699.1	ZNF396	zinc finger protein 396	0.76	1.06										
NP_001099040.1	NUDT19	nucleoside diphosphate-linked moiety X motif 19, mitochondrial precursor	1.22	1.06										
NP_001037.1	SLC12A2	solute carrier family 12 member 2	0.84	1.05								1.17	2.97	2.65
NP_001018149.1	NME2	nucleoside diphosphate kinase B isoform a	0.83	1.02										
NP_004580.1	SCO1	protein SCO1 homolog, mitochondrial precursor		1.02					0.71	0.93				
NP_002285.1	LAMP2	lysosome-associated membrane glycoprotein 2 isoform A precursor	0.93	1.02										
NP_003303.2	TST	thiosulfate sulfurtransferase	0.79	1.01										
NP_054737.1	MRPS28	28S ribosomal protein S28, mitochondrial	1.30	1.01					1.31	0.89				
NP_001344.2	AKR1C1	aldo-keto reductase family 1 member C1	0.29	1.01		0.69	3.47							
NP_002344.2	TACSTD2	tumor-associated calcium signal transducer 2 precursor	72.65	1.00								0.13	0.05	0.44

NP_066924.1	CLDN1	claudin-1		1.00							11.62	1.00	
NP_000985.1	RPL32	60S ribosomal protein L32		1.00				1.15	1.32	1.28			
NP_001155238.1	AHCY	adenosylhomocysteinase isoform 2	1.42	0.96									
NP_004530.1	NARS	asparaginyl-tRNA synthetase, cytoplasmic	0.87	0.92	0.74	1.18		0.84					
NP_006073.2	TUBA1B	tubulin alpha-1B chain	0.83	0.91				0.89	0.92	0.90			
NP_006397.1	PRDX4	peroxiredoxin-4	0.57	0.91									
NP_001131074.1	MEMO1	protein MEMO1 isoform 2	0.77	0.91									
NP_001203.1	C1QBP	complement component 1 Q subcomponent-binding protein, mitochondrial precursor	0.99	0.90				1.00	1.04	0.71			
NP_001182356.1	SRSF2	serine/arginine-rich splicing factor 2	0.90	0.90				1.01	0.98				
NP_942584.1	MTX1	metaxin-1 isoform 2		0.89									
NP_002435.1	MSN	moesin	0.62	0.88	0.70	1.09	3.32					1.98	
NP_004823.1	GSTO1	glutathione S-transferase omega-1 isoform 1	1.12	0.87	1.19	1.87	4.98						
NP_066402.2	HIST1H2BJ	histone H2B type 1-J	0.85	0.87									
NP_003042.3	SLC16A1	monocarboxylate transporter 1	0.80	0.86							0.18	0.28	0.47
NP_001136407.1	MXD3	max dimerization protein 3 isoform b	0.58	0.85									
NP_004883.2	SEC22B	vesicle-trafficking protein SEC22b	0.49	0.82				0.57	0.95				
NP_001655.1	RHOA	transforming protein RhoA precursor	0.70	0.80				0.80	0.69	0.96	0.46	1.11	
NP_076425.1	MRPS34	28S ribosomal protein S34, mitochondrial	0.99	0.79									
NP_000311.2	QDPR	dihydropteridine reductase	1.00	0.78									
NP_001077007.1	POTEE	POTE ankyrin domain family member E	0.75	0.77	0.25	1.78		0.60	0.70	0.81			
NP_919223.1	HNRNPA3	heterogeneous nuclear ribonucleoprotein A3	0.80	0.77				0.90	0.85				
NP_005165.1	ATP5C1	ATP synthase subunit gamma, mitochondrial isoform H (heart) precursor	0.61	0.77				0.77	0.78		0.27		0.51
NP_660298.2	MRPL10	39S ribosomal protein L10, mitochondrial isoform a precursor	0.97	0.74									
NP_001020131.1	ARPC4	actin-related protein 2/3 complex subunit 4 isoform b	0.83	0.74				0.92	0.74				
NP_006383.2	NOP56	nucleolar protein 56	0.69	0.74									
NP_057134.1	MRPL11	39S ribosomal protein L11, mitochondrial isoform a	0.67	0.72									
NP_003514.2	HIST1H2BE	histone H2B type 1-C/E/F/G/I	0.69	0.71									
NP_003119.2	SPTBN1	spectrin beta chain, brain 1 isoform 1	0.57	0.70									
NP_005712.1	ACTR3	actin-related protein 3	0.55	0.68									
NP_004484.1	HSD17B10	3-hydroxyacyl-CoA dehydrogenase type-2 isoform 1	0.67	0.68									
NP_004993.1	NDUFA9	NADH dehydrogenase [ubiquinone] 1 alpha subcomplex subunit 9, mitochondrial precursor	0.56	0.67									
NP_000265.1	OAT	ornithine aminotransferase, mitochondrial isoform 1 precursor	0.91	0.67									
NP_001075109.1	PRKDC	DNA-dependent protein kinase catalytic subunit isoform 2	0.63	0.66									
NP_008939.1	RTN4	reticulon-4 isoform C	0.56	0.65									
NP_001377.1	DPYSL2	dihydropyrimidinase-related protein 2 isoform 2	0.24	0.65	0.17	0.68	0.48	0.05	0.08				
NP_000512.1	HEXB	beta-hexosaminidase subunit beta preproprotein	0.67	0.65									
NP_006631.2	SEPT9	septin-9 isoform c	0.64	0.63				0.70	0.70				
NP_002991.2	SDHB	succinate dehydrogenase [ubiquinone] iron-sulfur subunit, mitochondrial precursor	0.77	0.62									
NP_613075.1	H2AFY	core histone macro-H2A.1 isoform 1		0.60				0.74	0.66	0.65			
NP_001123477.1	ACTN1	alpha-actinin-1 isoform c	0.68	0.60	0.78	0.84		0.69	0.54	0.87			
NP_066952.1	PPA1	inorganic pyrophosphatase	0.64	0.59									
NP_005110.2	THRAP3	thyroid hormone receptor-associated protein 3	1.63	0.59									
NP_006817.1	YWHAQ	14-3-3 protein theta	0.70	0.59				0.90	0.97				

NP_001137152.1	GSTK1	glutathione S-transferase kappa 1 isoform c	0.42	0.58									
NP_057376.2	TRAP1	heat shock protein 75 kDa, mitochondrial precursor	0.54	0.57									
NP_002203.1	EIF6	eukaryotic translation initiation factor 6 isoform a	1.21	0.54									
NP_658985.2	APOA1BP	apolipoprotein A-I-binding protein precursor		0.54									
NP_055416.2	EHD2	EH domain-containing protein 2	0.15	0.53									
NP_001654.1	ARF6	ADP-ribosylation factor 6	0.60	0.52									
NP_001670.1	ATP1B3	sodium/potassium-transporting ATPase subunit beta-3	0.44	0.52							0.36	1.14	1.99
NP_005318.3	HADH	hydroxyacyl-coenzyme A dehydrogenase, mitochondrial isoform 2 precursor		0.50									
XP_001718723.3	LOC100131514	mucin-3A-like	0.70	0.45									
NP_005882.2	ACAT2	acetyl-CoA acetyltransferase, cytosolic	0.32	0.45									
NP_001129612.1	CMPK1	UMP-CMP kinase isoform b	0.39	0.45									
NP_059980.2	TMED9	transmembrane emp24 domain-containing protein 9 precursor		0.44							0.04	0.04	
NP_001853.2	COX5B	cytochrome c oxidase subunit 5B, mitochondrial precursor	0.42	0.44							0.18	0.45	
NP_036364.2	PTRF	polymerase I and transcript release factor	0.50	0.43				0.50	0.42				
NP_003147.2	STIM1	stromal interaction molecule 1 precursor	0.51	0.43									
NP_149131.1	CALD1	caldesmon isoform 5	0.22	0.42									
NP_001161803.1	NDUFB4	NADH dehydrogenase [ubiquinone] 1 beta subcomplex subunit 4 isoform 2	0.61	0.41				0.65	1.00				
NP_006786.2	EHD1	EH domain-containing protein 1	0.56	0.39									
NP_203693.3	MYO1C	myosin-Ic isoform c	0.31	0.37							0.05	0.96	0.39
NP_001171500.1	ECI1	enoyl-CoA delta isomerase 1, mitochondrial isoform 2 precursor	0.35	0.36				0.33	0.17				
NP_001005189.1	OR6Y1	olfactory receptor 6Y1	0.80	0.35									
NP_958786.1	PLEC	plectin isoform 1a	0.56	0.35									
NP_644670.1	EHD4	EH domain-containing protein 4	0.78	0.33									
NP_001350.1	DECR1	2,4-dienoyl-CoA reductase, mitochondrial precursor	0.21	0.29				0.40	0.35		0.03	0.03	0.10
NP_060310.1	MRPL16	39S ribosomal protein L16, mitochondrial precursor	0.75	0.27									
NP_006654.2	PPP1R13L	relA-associated inhibitor	0.77	0.23									
NP_998816.1	SLC25A24	calcium-binding mitochondrial carrier protein SCaMC-1 isoform 2	0.44	0.19									
NP_004990.3	MYO6	myosin-VI	0.27	0.18									
NP_005313.1	HIST1H1B	histone H1.5	0.15	0.16				0.21	0.24	0.01			
NP_000427.1	OXCT1	succinyl-CoA:3-ketoacid-coenzyme A transferase 1, mitochondrial precursor	0.15	0.15				0.43					
NP_002696.3	PPL	periplakin	0.15	0.14					0.17				
NP_002274.1	KRT85	keratin, type II cuticular Hb5	0.16	0.12							0.02	0.02	
NP_001165806.1	PLS3	plastin-3 isoform 2		0.10	0.46	0.40	9.99						
NP_006077.2	TUBB3	tubulin beta-3 chain isoform 1	0.06	0.07	0.51	0.11	5.11				0.25	0.30	6.90
NP_001009186.1	CCT6A	T-complex protein 1 subunit zeta isoform b	0.04	0.04	0.94	1.69							
NP_005545.1	KRT6A	keratin, type II cytoskeletal 6A	0.10	0.03				0.05	0.02	0.03	0.04	0.02	0.25
NP_001447.2	FLNA	filamin-A isoform 1	0.03	0.03	0.01	0.01		0.02	0.02		0.06	0.06	
NP_000412.3	KRT10	keratin, type I cytoskeletal 10	0.03	0.03				0.02	0.02		0.02	0.02	1.91
NP_112598.2	EPPK1	epiplakin	0.03	0.03									
NP_005548.2	KRT16	keratin, type I cytoskeletal 16	0.02	0.02				0.02	0.04				
NP_003279.2	TPD52L2	tumor protein D54 isoform e	0.02	0.02									
NP_056138.1	UFL1	E3 UFM1-protein ligase 1		0.01									

NP_000217.2	KRT9	keratin, type I cytoskeletal 9	0.01	0.01		0.02	0.02	0.48	0.01	0.01		0.03	0.03	2.46
NP_787028.1	KRT79	keratin, type II cytoskeletal 79	0.01	0.01										
NP_001739.2	CAPN2	calpain-2 catalytic subunit isoform 1	1.32			0.70	0.71	1.33	0.94	0.51				
NP_002005.1	FKBP4	peptidyl-prolyl cis-trans isomerase FKBP4	1.23			1.54	2.37	4.54						
NP_001075.1	PLOD3	procollagen-lysine,2-oxoglutarate 5-dioxygenase 3 precursor	0.64											
NP_001106968.1	SEPT9	septin-9 isoform f										0.14	0.14	
NP_060713.1	SEPT11	septin-11										0.05	0.05	0.18
NP_942135.1	ACACA	acetyl-CoA carboxylase 1 isoform 4				0.91	2.60	50.48						
NP_001017992.1	ACTBL2	beta-actin-like protein 2				0.81	1.20							
NP_001093.1	ACTN1	alpha-actinin-1 isoform b										0.43	1.20	5.19
NP_001095.1	ACTN3	alpha-actinin-3				0.85	0.86							
NP_003650.1	AGPS	alkyldihydroxyacetonephosphate synthase, peroxisomal precursor											1.54	
NP_000678.1	AHCY	adenosylhomocysteinase isoform 1				1.46	2.11							
NP_000467.1	AK1	adenylate kinase isoenzyme 1				0.73	0.76	3.04	0.74	0.46				
NP_001189343.1	AKR1A1	alcohol dehydrogenase [NADP+]				0.62	0.89							
NP_000468.1	ALB	serum albumin preproprotein							0.02	0.02	0.80	0.02	0.02	1.30
NP_061877.1	ANKIB1	ankyrin repeat and IBR domain-containing protein 1							1.25	1.45				
NP_653299.3	ANKRD35	ankyrin repeat domain-containing protein 35							1.31	1.38				
NP_006392.1	ANP32B	acidic leucine-rich nuclear phosphoprotein 32 family member B				1.87	2.70	4.04	1.27	1.76	1.45			
NP_001621.2	ANXA8L2	annexin A8-like protein 2				6.06	4.11		69.58	1.00	121.41			
NP_036437.1	AP2A2	AP-2 complex subunit alpha-2 isoform 2							0.70	1.74		0.88	1.96	
NP_001651.1	ARF4	ADP-ribosylation factor 4							1.05	1.85				
NP_055976.1	ARL6IP1	ADP-ribosylation factor-like protein 6-interacting protein 1							0.49	1.43				
NP_005710.1	ARPC3	actin-related protein 2/3 complex subunit 3				0.92	1.00	4.54	0.46	0.43				
NP_005709.1	ARPC4	actin-related protein 2/3 complex subunit 4 isoform a										0.42	1.12	0.99
NP_001158228.1	ASPH	aspartyl/asparaginyl beta-hydroxylase isoform l							0.82	0.81				
NP_000692.2	ATP1A1	sodium/potassium-transporting ATPase subunit alpha-1 isoform a										0.37	0.84	1.48
NP_001668.1	ATP1B1	sodium/potassium-transporting ATPase subunit beta-1										0.23	0.15	
NP_001129237.1	ATP2A2	sarcoplasmic/endoplasmic reticulum calcium ATPase 2 isoform 3							0.03	2.99				
NP_001673.2	ATP2B1	plasma membrane calcium-transporting ATPase 1 isoform 1b										0.47	2.21	3.82
NP_001001396.1	ATP2B4	plasma membrane calcium-transporting ATPase 4 isoform 4a					2.37							
NP_001679.2	ATP5F1	ATP synthase subunit b, mitochondrial precursor							0.64	0.87	0.48	0.43	0.82	
NP_932071.1	BID	BH3-interacting domain death agonist isoform 3				8.73	20.18							
NP_000704.1	BLVRB	flavin reductase				0.57	1.02	1.74	0.41	0.69				
NP_940991.1	BSG	basigin isoform 2 precursor										0.59	1.01	3.50
NP_937798.3	C21orf33	ES1 protein homolog, mitochondrial isoform Ib precursor								0.58				
NP_057731.1	C8orf55	mesenchymal stem cell protein DSCD75 precursor							0.37	0.66				
NP_001007215.1	CACYBP	calcyclin-binding protein isoform 2				0.90	1.74							
NP_542157.1	CAPS	calcyphosin isoform b								1.00				

NP_001119527.1	CASK	peripheral plasma membrane protein CASK isoform 3							0.01	0.01	
NP_057671.2	CBX3	chromobox protein homolog 3				0.78	1.05				
NP_001120794.1	CBX5	chromobox protein homolog 5				0.64	1.47				
NP_005989.3	CCT3	T-complex protein 1 subunit gamma isoform a	1.21	1.68	5.08						
NP_001009570.1	CCT7	T-complex protein 1 subunit eta isoform b							0.55	1.19	
NP_001271.1	CIRBP	cold-inducible RNA-binding protein				0.75	0.84				
NP_039234.1	CLIC4	chloride intracellular channel protein 4	0.75	1.21	3.57	0.53	0.70		0.10	1.68	1.88
NP_620164.1	CMBL	carboxymethylenebutenolidase homolog	1.02	4.54							
NP_057392.1	CMPK1	UMP-CMP kinase isoform a	0.73	1.13	1.97	0.54	0.70				
NP_001120668.1	CNBP	cellular nucleic acid-binding protein isoform 6				1.16	1.28				
NP_055070.1	CNPY2	protein canopy homolog 2 isoform 1 precursor				0.79	1.94		0.17	0.35	
NP_009294.1	COMT	catechol O-methyltransferase isoform S-COMT	0.06	0.06	0.12	0.64	0.05				
NP_955476.1	COPE	coatomer subunit epsilon isoform c				0.88	1.13				
NP_937832.1	COPS8	COP9 signalosome complex subunit 8 isoform 2				0.71	1.00				
NP_001018080.1	CORO1B	coronin-1B							0.44	1.15	15.47
NP_055140.1	CORO1C	coronin-1C isoform 1							0.56	3.40	30.28
NP_066972.1	COTL1	coactosin-like protein				0.59	0.50	0.66			
NP_001185792.1	CPNE1	copine-1 isoform c	1.30	0.87	2.83						
NP_001180499.1	CSRP1	cysteine and glycine-rich protein 1 isoform 3				0.13	0.15		0.07	0.07	
NP_001899.1	CTSB	cathepsin B preproprotein					0.11				
NP_001014837.1	CUTA	protein CutA isoform 2	0.02	1.21	0.09						
NP_061820.1	CYCS	cytochrome c				0.77	1.14				
NP_004386.2	DBN1	drebrin isoform a				1.27	1.78		0.08	2.43	8.42
NP_001128513.1	DCTN1	dynactin subunit 1 isoform 4	2.23	1.49							
NP_005207.2	DDOST	dolichyl-diphosphooligosaccharide--protein glycosyltransferase 48 kDa subunit precursor							0.06	0.06	0.13
NP_006377.2	DDX17	probable ATP-dependent RNA helicase DDX17 isoform 1							1.55	1.91	
NP_076950.1	DDX50	ATP-dependent RNA helicase DDX50				1.14	2.65				
NP_001073280.1	DIAPH1	protein diaphanous homolog 1 isoform 2	0.80	0.52	4.88						
NP_057390.1	DNAJB11	dnaJ homolog subfamily B member 11 precursor							0.05	0.05	
NP_056005.1	DNAJC9	dnaJ homolog subfamily C member 9							2.06	2.24	7.08
NP_569710.2	DPP3	dipeptidyl peptidase 3	0.63	1.04	2.41						
NP_001008844.1	DSP	desmoplakin isoform II							0.45	0.47	
NP_001011546.1	DSTN	destrin isoform b				0.37	0.55		0.06	1.34	
NP_543010.3	DTD1	D-tyrosyl-tRNA(Tyr) deacylase 1				1.12	1.17				
NP_001158503.1	DTYMK	thymidylate kinase isoform 2				0.62	0.67				
NP_653166.2	DUOXA1	dual oxidase maturation factor 1	6.97	1.00							
NP_001020420.1	DUT	deoxyuridine 5'-triphosphate nucleotidohydrolase, mitochondrial isoform 3	1.21	1.59	1.96	0.93	0.96				
NP_001369.1	DYNC1I2	cytoplasmic dynein 1 intermediate chain 2	1.07	1.32							
NP_006570.1	EBP	3-beta-hydroxysteroid-Delta(8), Delta(7)-isomerase							0.28	0.34	
NP_694880.1	EDF1	endothelial differentiation-related factor 1 isoform beta				1.41	2.44				
NP_003557.2	EEA1	early endosome antigen 1	0.35								
NP_001123528.1	EEF1D	elongation factor 1-delta isoform 4	0.89	1.92		0.89	0.60				
NP_958440.1	EGFR	epidermal growth factor receptor isoform c precursor							13.49	18.48	
NP_001191439.1	EIF4A1	eukaryotic initiation factor 4A-I isoform 2				1.11	1.63				
NP_852131.1	EIF6	eukaryotic translation initiation factor 6 isoform c				1.26	0.98				

NP_001975.1	ESD	S-formylglutathione hydrolase	1.01	1.49	3.07						
NP_055703.1	FAM3C	family with sequence similarity 3, member C precursor				2.04	0.93				
NP_055744.2	FASTKD2	FAST kinase domain-containing protein 2				0.68	0.73				
NP_001129294.1	FDPS	farnesyl pyrophosphate synthase isoform b	1.01	1.82	15.08						
NP_001164408.1	FUS	RNA-binding protein FUS isoform 3				0.93	1.47	1.04			
NP_001035810.1	G6PD	glucose-6-phosphate 1-dehydrogenase isoform b	1.01	3.33	7.06						
NP_002038.2	GARS	glycyl-tRNA synthetase	0.68	1.24	2.75						
NP_056280.2	GEMIN5	gem-associated protein 5	29.93	1.00							
NP_005253.3	GFER	FAD-linked sulfhydryl oxidase ALR				0.34	0.31				
NP_001159897.1	GNAI2	guanine nucleotide-binding protein G(i) subunit alpha-2 isoform 2							0.57	2.29	
NP_006089.1	GNB2L1	guanine nucleotide-binding protein subunit beta-2-like 1				1.03	1.39				
NP_932332.1	GNPNAT1	glucosamine 6-phosphate N-acetyltransferase	1.06	1.39							
NP_000572.2	GPX1	glutathione peroxidase 1 isoform 1				0.36	0.22				
NP_079472.1	GRPEL1	grpE protein homolog 1, mitochondrial precursor				1.21	1.16				
NP_057001.1	GSTK1	glutathione S-transferase kappa 1 isoform a				0.37	0.41				
NP_001177931.1	GSTO1	glutathione S-transferase omega-1 isoform 2				0.85	0.88	0.91			
NP_002096.1	H2AFX	histone H2A.x					0.54				
NP_689953.1	HIBADH	3-hydroxyisobutyrate dehydrogenase, mitochondrial precursor				0.43	0.36		0.23	0.25	
NP_005312.1	HIST1H1E	histone H1.4				0.53		1.11			
NP_066409.1	HIST1H2AD	histone H2A type 1-D				0.75	0.81				
NP_066406.1	HIST1H2BB	histone H2B type 1-B	0.75	0.84	1.21						
NP_619790.1	HIST1H2BD	histone H2B type 1-D				0.61	0.71				
NP_003515.1	HIST1H2BH	histone H2B type 1-H				0.65	0.64				
NP_542160.1	HIST1H2BK	histone H2B type 1-K				0.71	0.76		0.70	0.71	1.24
NP_003510.1	HIST1H2BL	histone H2B type 1-L				1.04	1.01	1.16			
NP_003512.1	HIST1H2BM	histone H2B type 1-M				0.33	0.41				
NP_003511.1	HIST1H2BN	histone H2B type 1-N				0.60	0.71				
NP_001091678.1	HNRNPF	heterogeneous nuclear ribonucleoprotein F	1.42	1.93	6.20						
NP_055093.2	HSPA4L	heat shock 70 kDa protein 4L	1.57	1.60	7.02						
NP_004499.2	IDI1	isopentenyl-diphosphate Delta-isomerase 1	7.54	21.75	109.05						
NP_004506.2	ILF2	interleukin enhancer-binding factor 2				0.90	1.25	1.38			
NP_060755.1	IMP3	U3 small nucleolar ribonucleoprotein protein IMP3				1.85	1.46				
NP_001129674.1	ISOC2	isochorismatase domain-containing protein 2, mitochondrial isoform 3				0.84	1.28				
NP_002194.2	ITGA2	integrin alpha-2 precursor							1.20	1.78	11.84
NP_001138471.1	ITGAV	integrin alpha-V isoform 2							0.89	1.81	
NP_004512.1	KIF5B	kinesin-1 heavy chain	1.13	1.22	8.29						
NP_004976.2	KRAS	GTPase KRas isoform b precursor				0.61	0.36				
NP_778238.1	KRT73	keratin, type II cytoskeletal 73							0.05	0.05	
NP_002275.1	KRT86	keratin, type II cuticular Hb6	0.47								
NP_003928.1	KYNU	kynureninase isoform a	0.29	1.35							
NP_064502.9	LARS	leucyl-tRNA synthetase, cytoplasmic	1.07	1.47	3.50						
NP_001073585.1	LDB3	LIM domain-binding protein 3 isoform 4		0.29							
NP_060832.1	LIN7C	protein lin-7 homolog C							0.36	0.76	
NP_005563.1	LMNA	prelamin-A/C isoform 2				0.70	0.65	0.84	0.65	0.53	
XP_003119960.1	LOC100507556	putative uncharacterized protein NCRNA00269-like							0.42	0.64	

XP_003119966.1	LOC100507801	60S ribosomal protein L25-like				0.66	0.98				
XP_003119503.1	LOC100508006	aldo-keto reductase family 1 member C2-like isoform 3				0.18	0.99		0.07	1.16	0.03
XP_003119502.1	LOC100508006	aldo-keto reductase family 1 member C2-like isoform 2	0.06	0.93							
XP_003120348.1	LOC100508181	ran-specific GTPase-activating protein-like	1.16	1.48	3.60	1.04	1.02	1.03	0.61	0.94	
XP_003120000.1	LOC100508643	ras-related protein Rap-1b-like protein-like				0.70	1.06	0.66			
XP_001717228.2	LOC729595	high mobility group protein B3-like				1.09	0.47	0.16			
XP_001129414.1	LOC731751	DNA-dependent protein kinase catalytic subunit-like	1.13	1.30		0.50	0.52	0.43			
NP_001131025.1	LRRFIP1	leucine-rich repeat flightless-interacting protein 1 isoform 5		1.30	0.00						
NP_689557.1	LSM12	protein LSM12 homolog				1.11	2.15				
NP_000886.1	LTA4H	leukotriene A-4 hydrolase	1.03	1.56	2.98						
NP_006321.1	LYPLA1	acyl-protein thioesterase 1	0.58	0.71	2.01	0.37	0.34				
NP_054752.3	METTL7A	methyltransferase-like protein 7A precursor				0.16	1.07		0.05	0.82	
NP_054797.2	MRPL13	ras-related protein Rap-1A precursor							0.58	0.78	
NP_071344.1	MRPL17	39S ribosomal protein L17, mitochondrial precursor				0.86	1.01				
NP_000245.2	MTR	methionine synthase	1.09	2.83	48.82						
NP_057323.3	MYO15A	myosin-XV							0.54	0.90	1.61
NP_036355.2	MYO1B	myosin-Ib isoform 2							1.70	1.58	7.86
NP_005737.1	NAMPT	nicotinamide phosphoribosyltransferase precursor	5.91	25.48							
NP_660202.3	NAPRT1	nicotinate phosphoribosyltransferase	0.17								
NP_001139749.1	NCEH1	neutral cholesterol ester hydrolase 1 isoform c							0.45	1.56	5.83
NP_004995.1	NDUFB8	NADH dehydrogenase [ubiquinone] 1 beta subcomplex subunit 8, mitochondrial precursor				0.75	0.67				
NP_001186911.1	NDUFS1	NADH-ubiquinone oxidoreductase 75 kDa subunit, mitochondrial isoform 3							0.09	0.74	
NP_004542.1	NDUFS3	NADH dehydrogenase [ubiquinone] iron-sulfur protein 3, mitochondrial precursor				0.75	0.70				
NP_077718.3	NDUFS7	NADH dehydrogenase [ubiquinone] iron-sulfur protein 7, mitochondrial precursor				0.86	0.92				
NP_066552.2	NDUFV2	NADH dehydrogenase [ubiquinone] flavoprotein 2, mitochondrial precursor							0.42	0.98	
NP_060308.1	NHP2	H/ACA ribonucleoprotein complex subunit 2 isoform a				1.19	1.30				
NP_000260.1	NME1	nucleoside diphosphate kinase A isoform b				0.73	0.99	0.80			
NP_001018146.1	NME1-NME2	NME1-NME2 protein	1.06	1.86	3.25	0.80	0.94	0.92	0.59	1.10	
NP_006160.1	NNMT	nicotinamide N-methyltransferase	1.64	5.77	0.49						
NP_001032827.1	NPM1	nucleophosmin isoform 3	1.56	1.37	2.70						
NP_071355.1	NSUN3	putative methyltransferase NSUN3	0.93	1.64							
NP_002517.1	NT5E	5'-nucleotidase isoform 1 preproprotein							5.23	19.46	11.56
NP_001165376.1	NUDT16	U8 snoRNA-decapping enzyme isoform 3	1.48	0.66	42.17						
NP_001003941.1	OGDH	2-oxoglutarate dehydrogenase, mitochondrial isoform 2 precursor				1.08	1.02				
NP_112241.2	PABPC3	polyadenylate-binding protein 3				0.92	1.82		0.51	1.08	
NP_009193.2	PARK7	protein DJ-1	2.35	3.05	5.81	1.26	1.57	1.00			
NP_001609.2	PARP1	poly [ADP-ribose] polymerase 1				0.96					
NP_065090.1	PCNP	PEST proteolytic signal-containing nuclear protein				0.56	0.73				
NP_037364.1	PDCD6	programmed cell death protein 6	0.88	1.16							
NP_071736.1	PDF	peptide deformylase, mitochondrial precursor				0.49	0.71				

NP_001166927.1	PDHA1	pyruvate dehydrogenase E1 component subunit alpha, somatic form, mitochondrial isoform 4 precursor				1.32	1.71				
NP_066272.1	PDLIM1	PDZ and LIM domain protein 1							0.42	1.23	7.80
NP_036525.1	PFAS	phosphoribosylformylglycinamidine synthase	8.76	11.68	22.44						
NP_002618.1	PFKP	6-phosphofructokinase type C isoform 1	3.41	2.30	16.37						
NP_444252.1	PFN2	profilin-2 isoform a				0.52	1.28				
NP_002624.2	PGM1	phosphoglucomutase-1 isoform 1	0.86	1.19	1.72						
NP_006658.1	PGRMC1	membrane-associated progesterone receptor component 1				0.84	1.43	1.55			
NP_000287.3	PKD1	polycystin-1 isoform 2 precursor				1.16	1.38				
NP_958783.1	PLEC	plectin isoform 1d	0.46	0.48	30.21	0.58	0.37	0.41	0.40	0.48	
NP_857634.1	PRDX5	peroxiredoxin-5, mitochondrial isoform b precursor				0.24	0.10	0.27			
NP_009129.1	PROSC	proline synthase co-transcribed bacterial homolog protein	0.53	0.55	1.99	0.36					
NP_066977.1	PSAT1	phosphoserine aminotransferase isoform 2	1.83	6.26							
NP_002778.1	PSMA2	proteasome subunit alpha type-2	1.27	1.70	3.80	1.05	1.41	1.07			
NP_001096138.1	PSMA4	proteasome subunit alpha type-4 isoform 2	1.02	1.88		0.99	1.44				
NP_002781.2	PSMA5	proteasome subunit alpha type-5 isoform 1				0.87	1.35				
NP_002783.1	PSMA7	proteasome subunit alpha type-7				0.79	1.51				
NP_001020268.1	PSMA8	proteasome subunit alpha type-7-like isoform 3	1.04	1.88							
NP_001186708.1	PSMB2	proteasome subunit beta type-2 isoform 2	1.51	2.02							
NP_002786.2	PSMB3	proteasome subunit beta type-3				1.01	1.20	1.03			
NP_002787.2	PSMB4	proteasome subunit beta type-4				1.10	1.21				
NP_002809.2	PSME2	proteasome activator complex subunit 2				0.10	2.67				
NP_001093136.1	PTAR1	protein prenyltransferase alpha subunit repeat-containing protein 1				0.67	1.20		0.36	1.08	
NP_002815.3	PTMS	parathymosin				0.24	1.12				
NP_001157412.1	PYGL	glycogen phosphorylase, liver form isoform 2	7.19	5.16	51.68	15.24	1.00	398.23			
NP_004654.1	RAB11A	ras-related protein Rab-11A isoform 1				0.37	0.47		0.11	0.96	
NP_057406.2	RAB14	ras-related protein Rab-14				0.60	1.36	0.73			
NP_004152.1	RAB1A	ras-related protein Rab-1A isoform 1				0.75	1.04	1.26			
NP_002860.2	RAB6A	ras-related protein Rab-6A isoform a				0.87	1.01				
NP_005393.2	RALA	ras-related protein Ral-A precursor							0.34	0.50	3.75
NP_002872.1	RALB	ras-related protein Ral-B precursor							0.30	0.85	
NP_002875.1	RAP1A	ras-related protein Rap-1A precursor							0.57	2.20	
NP_002878.2	RARS	arginyl-tRNA synthetase, cytoplasmic							0.71	1.20	2.82
NP_006734.1	RBM3	putative RNA-binding protein 3				0.45	0.66				
NP_005096.1	RBM8A	RNA-binding protein 8A				1.20	1.32				
NP_061185.1	RCC2	protein RCC2				0.65		0.94			
NP_056338.2	REXO2	oligoribonuclease, mitochondrial precursor					1.39				
NP_001036143.1	RHOC	rho-related GTP-binding protein RhoC precursor				0.86	0.69				
NP_001656.2	RHOG	rho-related GTP-binding protein RhoG precursor				0.76	1.05				
NP_542784.1	RPL10L	60S ribosomal protein L10-like				0.99	1.16		0.52	0.92	
NP_000979.1	RPL27	60S ribosomal protein L27							0.33	0.20	0.91
NP_000982.2	RPL28	60S ribosomal protein L28 isoform 2				0.88	1.16	0.90	0.46	0.88	1.62
NP_001093163.1	RPL31	60S ribosomal protein L31 isoform 3	1.09	1.55	3.85						
NP_296374.1	RPL34	60S ribosomal protein L34				1.29	1.41				
NP_000988.1	RPL37	60S ribosomal protein L37				1.08	1.24				

NP_001016.1	RPS23	40S ribosomal protein S23				0.90	1.20	1.18	0.37	0.89	1.33
NP_001017.1	RPS24	40S ribosomal protein S24 isoform c				0.97	1.27				
NP_006657.1	RUVBL2	ruvB-like 2							0.78	1.24	2.66
NP_002957.1	S100A10	protein S100-A10							0.48	0.80	3.60
NP_055439.1	S100A6	protein S100-A6				0.30	0.25	0.34			
NP_006799.1	SEC61B	protein transport protein Sec61 subunit beta				1.17	2.48				
NP_003002.2	SET	protein SET isoform 2	1.80	2.21	4.46						
NP_006833.2	SF3B2	splicing factor 3B subunit 2				0.95	1.04	0.91			
NP_001035887.1	SLC16A3	monocarboxylate transporter 4							0.48	1.58	2.72
NP_005619.1	SLC1A5	neutral amino acid transporter B(0) isoform 1							1.59	4.43	6.34
NP_005975.1	SLC25A1	tricarboxylate transport protein, mitochondrial precursor				0.36	0.43				
NP_001158890.1	SLC25A11	mitochondrial 2-oxoglutarate/malate carrier protein isoform 3							0.38	0.81	1.27
NP_003477.4	SLC7A5	large neutral amino acids transporter small subunit 1				1.02	0.39	0.75	0.66	0.91	3.05
NP_112487.1	SLIRP	SRA stem-loop-interacting RNA-binding protein, mitochondrial precursor				1.25	1.66	1.73			
NP_570710.1	SNAP23	synaptosomal-associated protein 23 isoform SNAP23B							0.17	1.27	
NP_003078.2	SNCG	gamma-synuclein				2.47	0.69				
NP_004588.1	SNRPD2	small nuclear ribonucleoprotein Sm D2 isoform 1				0.92	1.16				
NP_000627.2	SOD2	superoxide dismutase [Mn], mitochondrial isoform A precursor				0.30	1.32				
NP_003962.3	SPAG9	C-Jun-amino-terminal kinase-interacting protein 4 isoform 3	0.54	0.93							
NP_004167.3	SREBF1	sterol regulatory element-binding protein 1 isoform b	0.87	0.56	2.55						
NP_001191123.1	SRP19	signal recognition particle 19 kDa protein isoform 3				0.90	1.14				
NP_067026.3	SRPRB	signal recognition particle receptor subunit beta							0.24	0.78	1.85
NP_008855.1	SRSF1	serine/arginine-rich splicing factor 1 isoform 1				1.00	1.13	0.84			
NP_006810.1	STIP1	stress-induced-phosphoprotein 1	1.41	2.00	4.79						
NP_004090.4	STOM	erythrocyte band 7 integral membrane protein isoform a							0.70	2.74	3.76
NP_001005849.1	SUMO2	small ubiquitin-related modifier 2 isoform b precursor				0.65	1.11				
NP_689508.3	TARS	threonyl-tRNA synthetase, cytoplasmic	1.26	1.63	14.73						
NP_006584.1	TBR1	T-box brain protein 1	1.58	2.22							
NP_110379.2	TCP1	T-complex protein 1 subunit alpha isoform a	1.06	0.25	3.69						
NP_001008897.1	TCP1	T-complex protein 1 subunit alpha isoform b				0.90	1.16	0.90			
NP_001180305.1	TERT	telomerase reverse transcriptase isoform 2				1.13	1.33				
NP_003225.2	TFRC	transferrin receptor protein 1				0.73	3.30				
NP_001027454.1	TMPO	thymopoietin isoform beta				0.79	1.31				
NP_061882.2	TOLLIP	toll-interacting protein							0.81	3.96	34.70
NP_064628.1	TOMM22	mitochondrial import receptor subunit TOM22 homolog				1.22	1.26				
NP_003277.1	TOP1	DNA topoisomerase 1				1.53	2.44	1.20			
NP_955391.1	TPD52L2	tumor protein D54 isoform f				0.55					
NP_001036817.1	TPM3	tropomyosin alpha-3 chain isoform 3							0.35	1.30	1.54
NP_003282.2	TPP2	tripeptidyl-peptidase 2	1.45	2.01	3.98						
NP_003286.1	TPT1	translationally-controlled tumor protein				1.59	1.05	2.81			
NP_116093.1	TUBA1C	tubulin alpha-1C chain	0.95	1.27	2.94				0.55	0.87	1.87
NP_877420.1	TXNRD1	thioredoxin reductase 1, cytoplasmic isoform 2		3.28							

NP_001107227.1	TYMP	thymidine phosphorylase precursor	0.20	0.34	0.01				0.17	0.31	
NP_005490.1	UBA2	SUMO-activating enzyme subunit 2	1.31	1.62							
NP_060697.4	UBA6	ubiquitin-like modifier-activating enzyme 6	1.06	0.94							
NP_066289.2	UBC	polyubiquitin-C	0.86	1.43							
NP_003338.1	UBE2L3	ubiquitin-conjugating enzyme E2 L3	1.32	1.83	3.93	0.63	0.84	0.82			
NP_444295.1	UBQLN1	ubiquilin-1 isoform 2	0.81	1.22							
NP_065816.2	UBR4	E3 ubiquitin-protein ligase UBR4							0.13	4.70	
NP_003355.1	UPP1	uridine phosphorylase 1	1.80	0.27		2.14		1.07			
NP_005994.2	UQCRFS1	cytochrome b-c1 complex subunit Rieske, mitochondrial				0.41	0.58				
NP_003361.1	VASP	vasodilator-stimulated phosphoprotein				0.58	0.45	0.46		0.99	
NP_006364.2	VAT1	synaptic vesicle membrane protein VAT-1 homolog							0.54	0.53	2.33
NP_060676.2	VPS35	vacuolar protein sorting-associated protein 35	0.62	0.64							
NP_998810.1	WARS	tryptophanyl-tRNA synthetase, cytoplasmic isoform b	1.43	3.84	6.95				0.79	2.46	
NP_059830.1	WDR1	WD repeat-containing protein 1 isoform 1	0.71	0.85	2.17						
NP_056053.1	WWC1	protein KIBRA isoform 3				0.91	1.22				
NP_000370.2	XDH	xanthine dehydrogenase/oxidase	18.95	5.29	125.53						
NP_055839.3	XPO7	exportin-7	0.52	0.55	4.10	0.38	0.46				
NP_009166.2	XPOT	exportin-T	7.30	17.19							
NP_004550.2	YBX1	nuclease-sensitive element-binding protein 1				0.50	0.97	0.60			
NP_036611.2	YWHAG	14-3-3 protein gamma	0.90	1.47	4.08	0.95	0.87	1.33	0.64	1.66	3.59
