# Supplementary material for: Integrated Proteomic and Metabolic Analysis of Breast Cancer Progression
Source: PLoS One. 2013 Sep 27;8(9):e76220. doi: 10.1371/journal.pone.0076220 (PMC3785415; doi:10.1371/journal.pone.0076220)
Supplement: Table S2 — Functional annotation clustering of cytosol enriched proteins. (PDF) [file pone.0076220.s002.pdf]

**Table S2.** Functional annotation clustering of cytosol enriched proteins

| Annotation Cluster 1 | Enrichment Score: 10.380168698390056 | | | | |
|---|---|---|---|---|---|
| Category | Term | Count | % | PValue | Fold Enrichment |
| GOTERM_BP_FAT | GO:0006007~glucose catabolic process | 12 | 9.09 | 5.5E-12 | 21.70 |
| GOTERM_BP_FAT | GO:0019320~hexose catabolic process | 12 | 9.09 | 4.0E-11 | 18.24 |
| GOTERM_BP_FAT | GO:0046365~monosaccharide catabolic process | 12 | 9.09 | 5.6E-11 | 17.72 |
| GOTERM_BP_FAT | GO:0046164~alcohol catabolic process | 12 | 9.09 | 2.4E-10 | 15.54 |

| Annotation Cluster 2 | Enrichment Score: 8.911831563001835 | | | | |
|---|---|---|---|---|---|
| Category | Term | Count | % | PValue | Fold Enrichment |
| SP_PIR_KEYWORDS | glycolysis | 10 | 7.58 | 1.4E-11 | 33.12 |
| GOTERM_BP_FAT | GO:0006096~glycolysis | 10 | 7.58 | 4.6E-10 | 22.31 |
| KEGG_PATHWAY | hsa00010:Glycolysis / Gluconeogenesis | 10 | 7.58 | 2.9E-07 | 10.46 |

| Annotation Cluster 3 | Enrichment Score: 8.028021927784092 | | | | |
|---|---|---|---|---|---|
| Category | Term | Count | % | PValue | Fold Enrichment |
| GOTERM_BP_FAT | GO:0044275~cellular carbohydrate catabolic process | 13 | 9.85 | 2.2E-11 | 16.04 |
| GOTERM_BP_FAT | GO:0016052~carbohydrate catabolic process | 13 | 9.85 | 4.4E-10 | 12.51 |
| GOTERM_BP_FAT | GO:0006006~glucose metabolic process | 13 | 9.85 | 2.2E-08 | 8.91 |
| GOTERM_BP_FAT | GO:0019318~hexose metabolic process | 13 | 9.85 | 2.7E-07 | 7.10 |
| GOTERM_BP_FAT | GO:0005996~monosaccharide metabolic process | 13 | 9.85 | 1.3E-06 | 6.14 |

| Annotation Cluster 4 | Enrichment Score: 6.117884930664276 | | | | |
|---|---|---|---|---|---|
| Category | Term | Count | % | PValue | Fold Enrichment |
| SP_PIR_KEYWORDS | ribosome | 10 | 7.58 | 1.6E-09 | 19.96 |
| GOTERM_CC_FAT | GO:0022626~cytosolic ribosome | 10 | 7.58 | 3.1E-08 | 14.09 |
| SP_PIR_KEYWORDS | ribosomal protein | 10 | 7.58 | 5.7E-06 | 7.75 |
| KEGG_PATHWAY | hsa03010:Ribosome | 10 | 7.58 | 7.1E-06 | 7.22 |
| SP_PIR_KEYWORDS | ribonucleoprotein | 10 | 7.58 | 1.3E-04 | 5.22 |

| Annotation Cluster 5 | Enrichment Score: 5.177701919800204 | | | | |
|---|---|---|---|---|---|
| Category | Term | Count | % | PValue | Fold Enrichment |
| SP_PIR_KEYWORDS | endonexin fold | 5 | 3.79 | 6.5E-07 | 66.24 |
| SMART | SM00335:ANX | 5 | 3.79 | 7.2E-07 | 64.85 |
| SP_PIR_KEYWORDS | annexin | 5 | 3.79 | 2.0E-06 | 52.04 |
| UP_SEQ_FEATURE | repeat:Annexin 1 | 5 | 3.79 | 2.0E-06 | 51.71 |
| UP_SEQ_FEATURE | repeat:Annexin 3 | 5 | 3.79 | 2.0E-06 | 51.71 |
| UP_SEQ_FEATURE | repeat:Annexin 2 | 5 | 3.79 | 2.0E-06 | 51.71 |
| UP_SEQ_FEATURE | repeat:Annexin 4 | 5 | 3.79 | 2.0E-06 | 51.71 |
| SP_PIR_KEYWORDS | calcium/phospholipid-binding | 5 | 3.79 | 2.6E-06 | 48.57 |
| INTERPRO | IPR018252:Annexin repeat, conserved site | 5 | 3.79 | 3.4E-06 | 45.07 |

| | | | | | |
|---|---|---|---|---|---|
| INTERPRO | IPR018502:Annexin repeat | 5 | 3.79 | 3.4E-06 | 45.07 |
| INTERPRO | IPR001464:Annexin | 5 | 3.79 | 3.4E-06 | 45.07 |
| GOTERM_MF_FAT | GO:0004859~phospholipase inhibitor activity | 5 | 3.79 | 3.6E-06 | 43.63 |
| GOTERM_MF_FAT | GO:0055102~lipase inhibitor activity | 5 | 3.79 | 9.7E-06 | 34.90 |
| SP_PIR_KEYWORDS | phospholipid binding | 5 | 3.79 | 1.4E-05 | 33.12 |
| PIR_SUPERFAMILY | PIRSF002359:annexin I | 5 | 3.79 | 1.5E-05 | 30.21 |
| GOTERM_MF_FAT | GO:0005544~calcium-dependent phospholipid binding | 5 | 3.79 | 9.8E-05 | 20.13 |
| GOTERM_MF_FAT | GO:0005543~phospholipid binding | 5 | 3.79 | 8.7E-02 | 2.96 |

| Annotation Cluster 6 | Enrichment Score: 4.793971688099384 | | | | |
|---|---|---|---|---|---|
| Category | Term | Count | % | PValue | Fold Enrichment |
| UP_SEQ_FEATURE | repeat:HEAT 6 | 6 | 4.55 | 1.7E-06 | 28.96 |
| UP_SEQ_FEATURE | repeat:HEAT 5 | 6 | 4.55 | 3.9E-06 | 24.82 |
| UP_SEQ_FEATURE | repeat:HEAT 4 | 6 | 4.55 | 1.1E-05 | 20.20 |
| UP_SEQ_FEATURE | repeat:HEAT 3 | 6 | 4.55 | 2.3E-05 | 17.38 |
| UP_SEQ_FEATURE | repeat:HEAT 2 | 6 | 4.55 | 4.8E-05 | 14.98 |
| UP_SEQ_FEATURE | repeat:HEAT 1 | 6 | 4.55 | 4.8E-05 | 14.98 |
| INTERPRO | IPR000357:HEAT | 6 | 4.55 | 7.1E-05 | 13.77 |

| Annotation Cluster 7 | Enrichment Score: 4.641813968720388 | | | | |
|---|---|---|---|---|---|
| Category | Term | Count | % | PValue | Fold Enrichment |
| SMART | SM00101:14_3_3 | 4 | 3.03 | 5.1E-06 | 103.76 |
| UP_SEQ_FEATURE | site:Interaction with phosphoserine on interacting protein | 4 | 3.03 | 1.1E-05 | 82.74 |
| INTERPRO | IPR000308:14-3-3 protein | 4 | 3.03 | 1.6E-05 | 72.12 |
| PIR_SUPERFAMILY | PIRSF000868:14-3-3 | 4 | 3.03 | 8.3E-05 | 41.43 |
| PIR_SUPERFAMILY | PIRSF000868:14-3-3 protein | 4 | 3.03 | 8.3E-05 | 41.43 |

| Annotation Cluster 8 | Enrichment Score: 4.460457489749598 | | | | |
|---|---|---|---|---|---|
| Category | Term | Count | % | PValue | Fold Enrichment |
| UP_SEQ_FEATURE | domain:Importin N-terminal | 5 | 3.79 | 2.0E-06 | 51.71 |
| INTERPRO | IPR001494:Importin-beta, N-terminal | 5 | 3.79 | 4.7E-06 | 42.07 |
| GOTERM_BP_FAT | GO:0000059~protein import into nucleus, docking | 5 | 3.79 | 1.7E-05 | 30.84 |
| GOTERM_MF_FAT | GO:0008565~protein transporter activity | 5 | 3.79 | 9.3E-03 | 6.02 |

| Annotation Cluster 9 | Enrichment Score: 4.145530740431766 | | | | |
|---|---|---|---|---|---|
| Category | Term | Count | % | PValue | Fold Enrichment |
| GOTERM_BP_FAT | GO:0042981~regulation of apoptosis | 21 | 15.91 | 6.4E-05 | 2.74 |
| GOTERM_BP_FAT | GO:0043067~regulation of programmed cell death | 21 | 15.91 | 7.4E-05 | 2.71 |
| GOTERM_BP_FAT | GO:0010941~regulation of cell death | 21 | 15.91 | 7.7E-05 | 2.70 |

| Annotation Cluster 10 | Enrichment Score: 3.9005096948506717 | | | | |
|---|---|---|---|---|---|
| Category | Term | Count | % | PValue | Fold Enrichment |

| | | | | | |
|---|---|---|---|---|---|
| INTERPRO | IPR013126:Heat shock protein 70 | 4 | 3.03 | 9.9E-05 | 42.07 |
| INTERPRO | IPR001023:Heat shock protein Hsp70 | 4 | 3.03 | 9.9E-05 | 42.07 |
| INTERPRO | IPR018181:Heat shock protein 70, conserved site | 4 | 3.03 | 2.0E-04 | 33.65 |

Annotation Cluster 11 Enrichment Score: 3.234845013098904

| Category | Term | Count | % | PValue | Fold Enrichment |
|---|---|---|---|---|---|
| GOTERM_BP_FAT | GO:0043066~negative regulation of apoptosis | 12 | 9.09 | 5.3E-04 | 3.55 |
| GOTERM_BP_FAT | GO:0043069~negative regulation of programmed cell death | 12 | 9.09 | 6.0E-04 | 3.51 |
| GOTERM_BP_FAT | GO:0060548~negative regulation of cell death | 12 | 9.09 | 6.1E-04 | 3.50 |

Annotation Cluster 12 Enrichment Score: 3.155627276223362

| Category | Term | Count | % | PValue | Fold Enrichment |
|---|---|---|---|---|---|
| UP_SEQ_FEATURE | repeat:HEAT 23 | 3 | 2.27 | 2.8E-04 | 108.60 |
| UP_SEQ_FEATURE | repeat:HEAT 22 | 3 | 2.27 | 2.8E-04 | 108.60 |
| UP_SEQ_FEATURE | repeat:HEAT 21 | 3 | 2.27 | 2.8E-04 | 108.60 |
| UP_SEQ_FEATURE | repeat:HEAT 20 | 3 | 2.27 | 2.8E-04 | 108.60 |
| UP_SEQ_FEATURE | repeat:HEAT 24 | 3 | 2.27 | 2.8E-04 | 108.60 |
| UP_SEQ_FEATURE | repeat:HEAT 16 | 3 | 2.27 | 2.8E-04 | 108.60 |
| UP_SEQ_FEATURE | repeat:HEAT 17 | 3 | 2.27 | 2.8E-04 | 108.60 |
| UP_SEQ_FEATURE | repeat:HEAT 18 | 3 | 2.27 | 2.8E-04 | 108.60 |
| UP_SEQ_FEATURE | repeat:HEAT 19 | 3 | 2.27 | 2.8E-04 | 108.60 |
| UP_SEQ_FEATURE | repeat:HEAT 15 | 3 | 2.27 | 9.6E-04 | 62.06 |
| UP_SEQ_FEATURE | repeat:HEAT 14 | 3 | 2.27 | 1.6E-03 | 48.27 |
| UP_SEQ_FEATURE | repeat:HEAT 13 | 3 | 2.27 | 2.0E-03 | 43.44 |
| UP_SEQ_FEATURE | repeat:HEAT 12 | 3 | 2.27 | 2.0E-03 | 43.44 |
| UP_SEQ_FEATURE | repeat:HEAT 11 | 3 | 2.27 | 2.5E-03 | 39.49 |
| UP_SEQ_FEATURE | repeat:HEAT 9 | 3 | 2.27 | 4.6E-03 | 28.96 |
| UP_SEQ_FEATURE | repeat:HEAT 10 | 3 | 2.27 | 4.6E-03 | 28.96 |

Annotation Cluster 13 Enrichment Score: 3.098788033864193

| Category | Term | Count | % | PValue | Fold Enrichment |
|---|---|---|---|---|---|
| GOTERM_BP_FAT | GO:0043623~cellular protein complex assembly | 10 | 7.58 | 2.3E-05 | 6.47 |
| GOTERM_BP_FAT | GO:0034622~cellular macromolecular complex assembly | 10 | 7.58 | 3.2E-03 | 3.30 |
| GOTERM_BP_FAT | GO:0034621~cellular macromolecular complex subunit organization | 10 | 7.58 | 6.8E-03 | 2.94 |

Annotation Cluster 14 Enrichment Score: 3.0058451605448546

| Category | Term | Count | % | PValue | Fold Enrichment |
|---|---|---|---|---|---|
| GOTERM_MF_FAT | GO:0032553~ribonucleotide binding | 32 | 24.24 | 7.7E-04 | 1.82 |
| GOTERM_MF_FAT | GO:0032555~purine ribonucleotide binding | 32 | 24.24 | 7.7E-04 | 1.82 |
| GOTERM_MF_FAT | GO:0017076~purine nucleotide binding | 32 | 24.24 | 1.6E-03 | 1.75 |

Annotation Cluster 15 Enrichment Score: 2.986663397389544

| Category | Term | Count | % | PValue | Fold Enrichment |
|---|---|---|---|---|---|
| UP_SEQ_FEATURE | active site:Cysteine sulfenic acid (-SOH) intermediate | 4 | 3.03 | 6.2E-06 | 96.53 |
| | | | | | |
| INTERPRO | IPR000866:Alkyl hydroperoxide reductase/ Thiol specific antioxidant/ Mal allergen | 4 | 3.03 | 1.6E-05 | 72.12 |
| SP_PIR_KEYWORDS | antioxidant | 4 | 3.03 | 2.5E-05 | 64.76 |
| GOTERM_MF_FAT | GO:0051920~peroxiredoxin activity | 4 | 3.03 | 4.5E-05 | 52.35 |
| SP_PIR_KEYWORDS | peroxidase | 4 | 3.03 | 6.4E-04 | 23.32 |
| UP_SEQ_FEATURE | domain:Thioredoxin | 4 | 3.03 | 1.5E-03 | 17.55 |
| GOTERM_BP_FAT | GO:0034614~cellular response to reactive oxygen species | 4 | 3.03 | 2.8E-03 | 13.98 |
| INTERPRO | IPR017936:Thioredoxin-like | 4 | 3.03 | 2.8E-03 | 14.02 |
| SP_PIR_KEYWORDS | Redox-active center | 4 | 3.03 | 2.9E-03 | 13.88 |
| GOTERM_MF_FAT | GO:0004601~peroxidase activity | 4 | 3.03 | 3.4E-03 | 13.09 |
| GOTERM_MF_FAT | GO:0016684~oxidoreductase activity, acting on peroxide as acceptor | 4 | 3.03 | 3.4E-03 | 13.09 |
| GOTERM_BP_FAT | GO:0034599~cellular response to oxidative stress | 4 | 3.03 | 7.7E-03 | 9.76 |
| GOTERM_MF_FAT | GO:0016209~antioxidant activity | 4 | 3.03 | 9.9E-03 | 8.91 |
| GOTERM_BP_FAT | GO:0045454~cell redox homeostasis | 4 | 3.03 | 2.2E-02 | 6.66 |
| GOTERM_BP_FAT | GO:0000302~response to reactive oxygen species | 4 | 3.03 | 3.4E-02 | 5.59 |
| | | | | | |
| Annotation Cluster 16 | Enrichment Score: 2.9553752490507255 | | | | |
| Category | Term | Count | % | PValue | Fold Enrichment |
| GOTERM_BP_FAT | GO:0046496~nicotinamide nucleotide metabolic process | 5 | 3.79 | 5.4E-04 | 13.11 |
| GOTERM_BP_FAT | GO:0006769~nicotinamide metabolic process | 5 | 3.79 | 5.4E-04 | 13.11 |
| GOTERM_BP_FAT | GO:0009820~alkaloid metabolic process | 5 | 3.79 | 5.9E-04 | 12.79 |
| GOTERM_BP_FAT | GO:0019362~pyridine nucleotide metabolic process | 5 | 3.79 | 6.5E-04 | 12.48 |
| GOTERM_BP_FAT | GO:0006733~oxidoreduction coenzyme metabolic process | 5 | 3.79 | 1.5E-03 | 10.08 |
| GOTERM_BP_FAT | GO:0043603~cellular amide metabolic process | 5 | 3.79 | 1.9E-03 | 9.36 |
| GOTERM_BP_FAT | GO:0019748~secondary metabolic process | 5 | 3.79 | 6.7E-03 | 6.64 |
| | | | | | |
| Annotation Cluster 17 | Enrichment Score: 2.9081886504762418 | | | | |
| Category | Term | Count | % | PValue | Fold Enrichment |
| GOTERM_CC_FAT | GO:0005643~nuclear pore | 6 | 4.55 | 6.1E-04 | 8.67 |
| GOTERM_BP_FAT | GO:0006606~protein import into nucleus | 6 | 4.55 | 1.3E-03 | 7.32 |
| GOTERM_BP_FAT | GO:0051170~nuclear import | 6 | 4.55 | 1.5E-03 | 7.15 |
| GOTERM_BP_FAT | GO:0034504~protein localization in nucleus | 6 | 4.55 | 2.0E-03 | 6.69 |
| | | | | | |
| Annotation Cluster 18 | Enrichment Score: 2.893563616432318 | | | | |
| Category | Term | Count | % | PValue | Fold Enrichment |
| GOTERM_BP_FAT | GO:0070271~protein complex biogenesis | 15 | 11.36 | 3.0E-04 | 3.11 |
| GOTERM_BP_FAT | GO:0006461~protein complex assembly | 15 | 11.36 | 3.0E-04 | 3.11 |
| GOTERM_BP_FAT | GO:0065003~macromolecular complex assembly | 15 | 11.36 | 4.1E-03 | 2.37 |
| GOTERM_BP_FAT | GO:0043933~macromolecular complex subunit organization | 15 | 11.36 | 7.2E-03 | 2.22 |

| Annotation Cluster 19 | Enrichment Score: 2.8087837900394588 | | | | |
|---|---|---|---|---|---|
| Category | Term | Count | % | PValue | Fold Enrichment |
| SMART | SM00262:GEL | 3 | 2.27 | 7.8E-04 | 68.09 |
| UP_SEQ_FEATURE | repeat:Gelsolin-like 1 | 3 | 2.27 | 1.3E-03 | 54.30 |
| UP_SEQ_FEATURE | repeat:Gelsolin-like 2 | 3 | 2.27 | 1.3E-03 | 54.30 |
| UP_SEQ_FEATURE | repeat:Gelsolin-like 3 | 3 | 2.27 | 1.3E-03 | 54.30 |
| INTERPRO | IPR007122:Gelsolin | 3 | 2.27 | 1.7E-03 | 47.33 |
| INTERPRO | IPR007123:Gelsolin region | 3 | 2.27 | 5.2E-03 | 27.04 |

| Annotation Cluster 20 | Enrichment Score: 2.7952415595592583 | | | | |
|---|---|---|---|---|---|
| Category | Term | Count | % | PValue | Fold Enrichment |
| SP_PIR_KEYWORDS | pentose shunt | 3 | 2.27 | 1.4E-04 | 145.72 |
| GOTERM_BP_FAT | GO:0006098~pentose-phosphate shunt | 3 | 2.27 | 3.1E-03 | 34.96 |
| GOTERM_BP_FAT | GO:0006739~NADP metabolic process | 3 | 2.27 | 9.8E-03 | 19.66 |

| Annotation Cluster 21 | Enrichment Score: 2.350023807460341 | | | | |
|---|---|---|---|---|---|
| Category | Term | Count | % | PValue | Fold Enrichment |
| INTERPRO | IPR002194:Chaperonin TCP-1, conserved site | 3 | 2.27 | 2.6E-03 | 37.86 |
| INTERPRO | IPR017998:Chaperone, tailless complex polypeptide 1 | 3 | 2.27 | 3.2E-03 | 34.42 |
| INTERPRO | IPR002423:Chaperonin Cpn60/TCP-1 | 3 | 2.27 | 6.0E-03 | 25.24 |
| PIR_SUPERFAMILY | PIRSF002584:molecular chaperone t-complex-type | 3 | 2.27 | 7.7E-03 | 21.75 |

| Annotation Cluster 22 | Enrichment Score: 1.9426658366719283 | | | | |
|---|---|---|---|---|---|
| Category | Term | Count | % | PValue | Fold Enrichment |
| GOTERM_BP_FAT | GO:0032507~maintenance of protein location in cell | 4 | 3.03 | 6.8E-03 | 10.23 |
| GOTERM_BP_FAT | GO:0045185~maintenance of protein location | 4 | 3.03 | 1.0E-02 | 8.74 |
| GOTERM_BP_FAT | GO:0051651~maintenance of location in cell | 4 | 3.03 | 1.0E-02 | 8.74 |
| GOTERM_BP_FAT | GO:0051235~maintenance of location | 4 | 3.03 | 2.3E-02 | 6.55 |

| Annotation Cluster 23 | Enrichment Score: 1.9076713225639956 | | | | |
|---|---|---|---|---|---|
| Category | Term | Count | % | PValue | Fold Enrichment |
| INTERPRO | IPR019479:Peroxiredoxin, C-terminal | 3 | 2.27 | 6.0E-04 | 75.72 |
| PIR_SUPERFAMILY | PIRSF000239:alkyl hydroperoxide reductase C22 protein | 3 | 2.27 | 1.8E-03 | 43.51 |
| GOTERM_BP_FAT | GO:0042744~hydrogen peroxide catabolic process | 3 | 2.27 | 1.1E-02 | 18.51 |
| GOTERM_BP_FAT | GO:0070301~cellular response to hydrogen peroxide | 3 | 2.27 | 1.2E-02 | 17.48 |
| GOTERM_BP_FAT | GO:0042743~hydrogen peroxide metabolic process | 3 | 2.27 | 2.3E-02 | 12.58 |
| GOTERM_BP_FAT | GO:0042542~response to hydrogen peroxide | 3 | 2.27 | 9.8E-02 | 5.62 |
| GOTERM_BP_FAT | GO:0006800~oxygen and reactive oxygen species metabolic process | 3 | 2.27 | 1.3E-01 | 4.70 |

| Annotation Cluster 24 | Enrichment Score: 1.7055341777652488 | | | | |
|---|---|---|---|---|---|
| Category | Term | Count | % | PValue | Fold Enrichment |
| GOTERM_MF_FAT | GO:0032559~adenyl ribonucleotide binding | 24 | 18.18 | 1.3E-02 | 1.68 |

| | | | | | |
|---|---|---|---|---|---|
| GOTERM_MF_FAT | GO:0030554~adenyl nucleotide binding | 24 | 18.18 | 2.3E-02 | 1.59 |
| GOTERM_MF_FAT | GO:0001883~purine nucleoside binding | 24 | 18.18 | 2.6E-02 | 1.57 |
