# Supplementary material for: Integrated Proteomic and Metabolic Analysis of Breast Cancer Progression
Source: PLoS One. 2013 Sep 27;8(9):e76220. doi: 10.1371/journal.pone.0076220 (PMC3785415; doi:10.1371/journal.pone.0076220)
Supplement: Table S3 — Functional annotation clustering of mitochondrial enriched proteins. (PDF) [file pone.0076220.s003.pdf]

Table S3. Functional annotation clustering of mitochondrial enriched proteins

Annotation Cluster 1	Enrichment Score: 7.102275595840315				
Category	Term	Count	%	PValue	Fold Enrichment
UP_SEQ_FEATURE	region of interest:Coil 2	7	14.58	1.07E-08	43.55
UP_SEQ_FEATURE	region of interest:Linker 12	7	14.58	1.07E-08	43.55
UP_SEQ_FEATURE	region of interest:Coil 1B	7	14.58	1.85E-08	39.82
UP_SEQ_FEATURE	region of interest:Coil 1A	7	14.58	1.85E-08	39.82
UP_SEQ_FEATURE	region of interest:Linker 1	7	14.58	1.85E-08	39.82
UP_SEQ_FEATURE	region of interest:Rod	7	14.58	2.02E-08	39.26
UP_SEQ_FEATURE	region of interest:Head	7	14.58	2.39E-08	38.18
UP_SEQ_FEATURE	region of interest:Tail	7	14.58	2.81E-08	37.16
SP_PIR_KEYWORDS	Intermediate filament	7	14.58	3.44E-08	35.96
INTERPRO	IPR018039:Intermediate filament protein, conserved site	7	14.58	4.92E-08	33.74
INTERPRO	IPR016044:Filament	7	14.58	4.92E-08	33.74
INTERPRO	IPR001664:Intermediate filament protein	7	14.58	5.35E-08	33.28
PIR_SUPERFAMILY	PIRSF002282:cytoskeletal keratin	7	14.58	4.67E-07	22.33
GOTERM_CC_FAT	GO:0005882~intermediate filament	7	14.58	3.56E-05	10.87
GOTERM_CC_FAT	GO:0045111~intermediate filament cytoskeleton	7	14.58	4.02E-05	10.63

Annotation Cluster 2	Enrichment Score: 3.5973888394005247				
Category	Term	Count	%	PValue	Fold Enrichment
INTERPRO	IPR003054:Type II keratin	4	8.33	6.56E-05	49.58
UP_SEQ_FEATURE	site:Stutter	4	8.33	7.22E-05	48.27
GOTERM_CC_FAT	GO:0045095~keratin filament	4	8.33	3.41E-03	12.91

Annotation Cluster 3	Enrichment Score: 3.2303540206247274				
Category	Term	Count	%	PValue	Fold Enrichment
GOTERM_BP_FAT	GO:0032507~maintenance of protein location in cell	4	8.33	3.36E-04	28.69
GOTERM_BP_FAT	GO:0051651~maintenance of location in cell	4	8.33	5.36E-04	24.51
GOTERM_BP_FAT	GO:0045185~maintenance of protein location	4	8.33	5.36E-04	24.51
GOTERM_BP_FAT	GO:0051235~maintenance of location	4	8.33	1.24E-03	18.38

Annotation Cluster 4	Enrichment Score: 2.5370204671851457				
Category	Term	Count	%	PValue	Fold Enrichment
GOTERM_BP_FAT	GO:0016477~cell migration	6	12.50	2.14E-03	6.39
GOTERM_BP_FAT	GO:0051674~localization of cell	6	12.50	3.38E-03	5.75
GOTERM_BP_FAT	GO:0048870~cell motility	6	12.50	3.38E-03	5.75

Annotation Cluster 5	Enrichment Score: 2.4451900472245636				
Category	Term	Count	%	PValue	Fold Enrichment
GOTERM_CC_FAT	GO:0008305~integrin complex	4	8.33	1.31E-04	39.18

SP_PIR_KEYWORDS	integrin	4	8.33	1.37E-04	39.10
GOTERM_BP_FAT	GO:0007229~integrin-mediated signaling pathway	4	8.33	1.61E-03	16.80
GOTERM_BP_FAT	GO:0007160~cell-matrix adhesion	4	8.33	3.20E-03	13.22
GOTERM_BP_FAT	GO:0031589~cell-substrate adhesion	4	8.33	4.20E-03	12.00
GOTERM_CC_FAT	GO:0043235~receptor complex	4	8.33	7.36E-03	9.79
KEGG_PATHWAY	hsa04512:ECM-receptor interaction	4	8.33	1.28E-02	7.81
SP_PIR_KEYWORDS	receptor	4	8.33	7.55E-01	1.01

Annotation Cluster 6 Enrichment Score: 2.2738113902647066

Category	Term	Count	%	PValue	Fold Enrichment
UP_SEQ_FEATURE	short sequence motif:Effector region	4	8.33	1.65E-03	16.77
INTERPRO	IPR013753:Ras	4	8.33	5.26E-03	11.11
INTERPRO	IPR001806:Ras GTPase	4	8.33	6.51E-03	10.28
SP_PIR_KEYWORDS	prenylation	4	8.33	6.53E-03	10.28
INTERPRO	IPR005225:Small GTP-binding protein	4	8.33	1.16E-02	8.31

Annotation Cluster 7 Enrichment Score: 2.0849452533958215

Category	Term	Count	%	PValue	Fold Enrichment
GOTERM_CC_FAT	GO:0005912~adherens junction	5	10.42	1.94E-03	9.16
GOTERM_CC_FAT	GO:0070161~anchoring junction	5	10.42	2.83E-03	8.26
GOTERM_CC_FAT	GO:0030054~cell junction	5	10.42	1.02E-01	2.74

Annotation Cluster 8 Enrichment Score: 1.7775336376209747

Category	Term	Count	%	PValue	Fold Enrichment
GOTERM_BP_FAT	GO:0042981~regulation of apoptosis	8	16.67	1.60E-02	2.93
GOTERM_BP_FAT	GO:0043067~regulation of programmed cell death	8	16.67	1.69E-02	2.90
GOTERM_BP_FAT	GO:0010941~regulation of cell death	8	16.67	1.72E-02	2.89

Annotation Cluster 9 Enrichment Score: 1.7471630816038997

Category	Term	Count	%	PValue	Fold Enrichment
UP_SEQ_FEATURE	nucleotide phosphate-binding region:GTP	5	10.42	5.89E-03	6.75
SP_PIR_KEYWORDS	gtp-binding	5	10.42	8.41E-03	6.09
GOTERM_MF_FAT	GO:0005525~GTP binding	5	10.42	3.15E-02	4.06
GOTERM_MF_FAT	GO:0019001~guanyl nucleotide binding	5	10.42	3.43E-02	3.95
GOTERM_MF_FAT	GO:0032561~guanyl ribonucleotide binding	5	10.42	3.43E-02	3.95

Annotation Cluster 10 Enrichment Score: 1.5916771030995296

Category	Term	Count	%	PValue	Fold Enrichment
GOTERM_BP_FAT	GO:0008064~regulation of actin polymerization or depolymerization	3	6.25	1.75E-02	14.46
GOTERM_BP_FAT	GO:0030832~regulation of actin filament length	3	6.25	1.86E-02	14.00
GOTERM_BP_FAT	GO:0032956~regulation of actin cytoskeleton organization	3	6.25	3.53E-02	9.91
GOTERM_BP_FAT	GO:0032970~regulation of actin filament-based process	3	6.25	3.75E-02	9.59

Annotation Cluster 11 Enrichment Score: 1.4678664420681806

Category	Term	Count	%	PValue	Fold Enrichment
GOTERM_CC_FAT	GO:0030018~Z disc	3	6.25	1.09E-02	18.52
GOTERM_CC_FAT	GO:0031674~I band	3	6.25	1.53E-02	15.49
GOTERM_CC_FAT	GO:0030017~sarcomere	3	6.25	4.47E-02	8.70
GOTERM_CC_FAT	GO:0030016~myofibril	3	6.25	5.58E-02	7.68
GOTERM_CC_FAT	GO:0044449~contractile fiber part	3	6.25	5.76E-02	7.54
GOTERM_CC_FAT	GO:0043292~contractile fiber	3	6.25	6.50E-02	7.04

Annotation Cluster 12 Enrichment Score: 1.3667793271236799

Category	Term	Count	%	PValue	Fold Enrichment
GOTERM_BP_FAT	GO:0048514~blood vessel morphogenesis	4	8.33	3.29E-02	5.58
GOTERM_BP_FAT	GO:0001568~blood vessel development	4	8.33	4.77E-02	4.80
GOTERM_BP_FAT	GO:0001944~vasculature development	4	8.33	5.06E-02	4.69

Annotation Cluster 13 Enrichment Score: 1.3613681043910073

Category	Term	Count	%	PValue	Fold Enrichment
BIOCARTA	h_malPathway:Role of MAL in Rho-Mediated Activation of SRF	3	6.25	8.49E-03	18.91
BIOCARTA	h_rasPathway:Ras Signaling Pathway	3	6.25	9.39E-03	17.96
BIOCARTA	h_p38mapkPathway:p38 MAPK Signaling Pathway	3	6.25	2.47E-02	10.89
KEGG_PATHWAY	hsa05211:Renal cell carcinoma	3	6.25	6.35E-02	7.03
KEGG_PATHWAY	hsa04370:VEGF signaling pathway	3	6.25	7.16E-02	6.56
UP_SEQ_FEATURE	propeptide:Removed in mature form	3	6.25	1.09E-01	5.22
KEGG_PATHWAY	hsa04062:Chemokine signaling pathway	3	6.25	3.03E-01	2.63

Annotation Cluster 14 Enrichment Score: 1.3555925712828896

Category	Term	Count	%	PValue	Fold Enrichment
GOTERM_BP_FAT	GO:0070271~protein complex biogenesis	6	12.50	2.54E-02	3.49
GOTERM_BP_FAT	GO:0006461~protein complex assembly	6	12.50	2.54E-02	3.49
GOTERM_BP_FAT	GO:0065003~macromolecular complex assembly	6	12.50	6.85E-02	2.65
GOTERM_BP_FAT	GO:0043933~macromolecular complex subunit organization	6	12.50	8.53E-02	2.49
