# Supplementary figures and images for: Integrated Proteomic and Metabolic Analysis of Breast Cancer Progression
Source: PLoS One. 2013 Sep 27;8(9):e76220. doi: 10.1371/journal.pone.0076220 (PMC3785415; doi:10.1371/journal.pone.0076220)

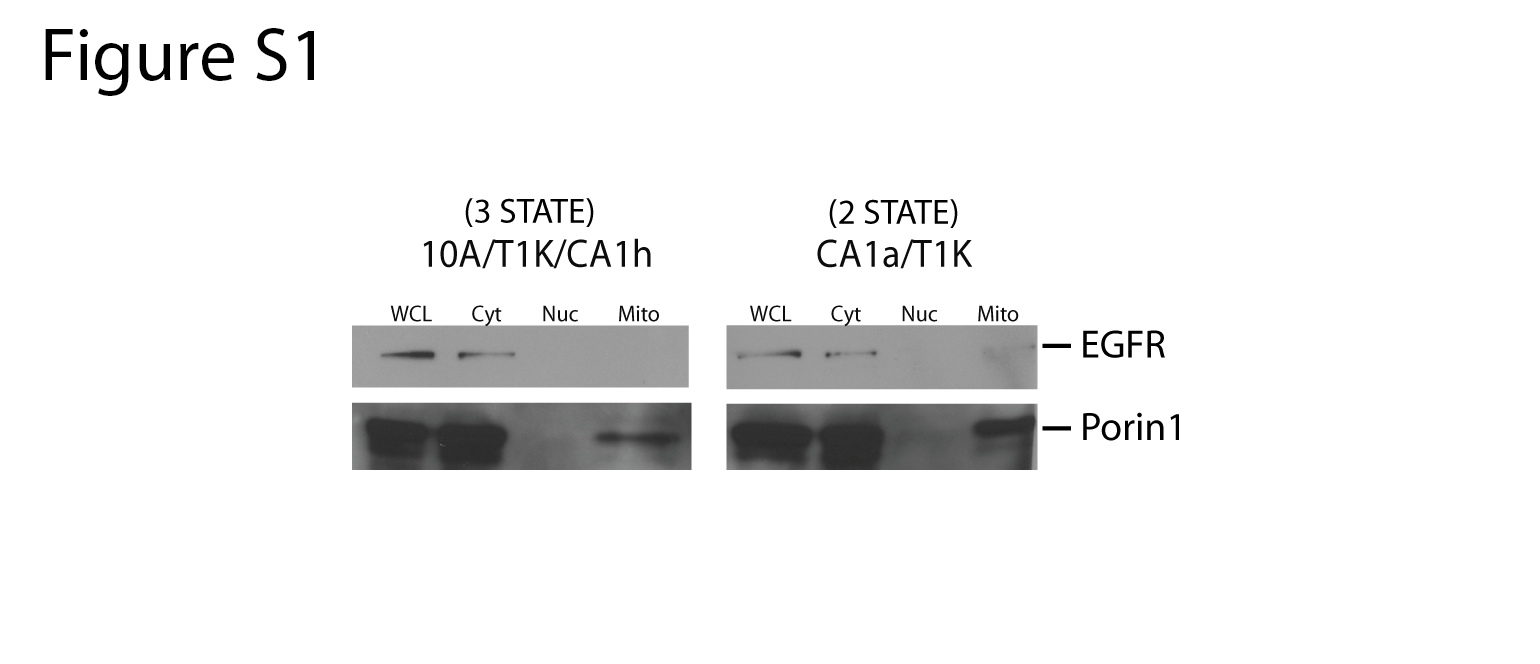

Supplement: Figure S1 — Western blot of approximately 25 µg of SILAC whole cell lysate, as well as cytosolic, nuclear and mitochondrial fractions, probed for EGFR and Mitochondrial Porin. (TIF) [file pone.0076220.s004.tif]

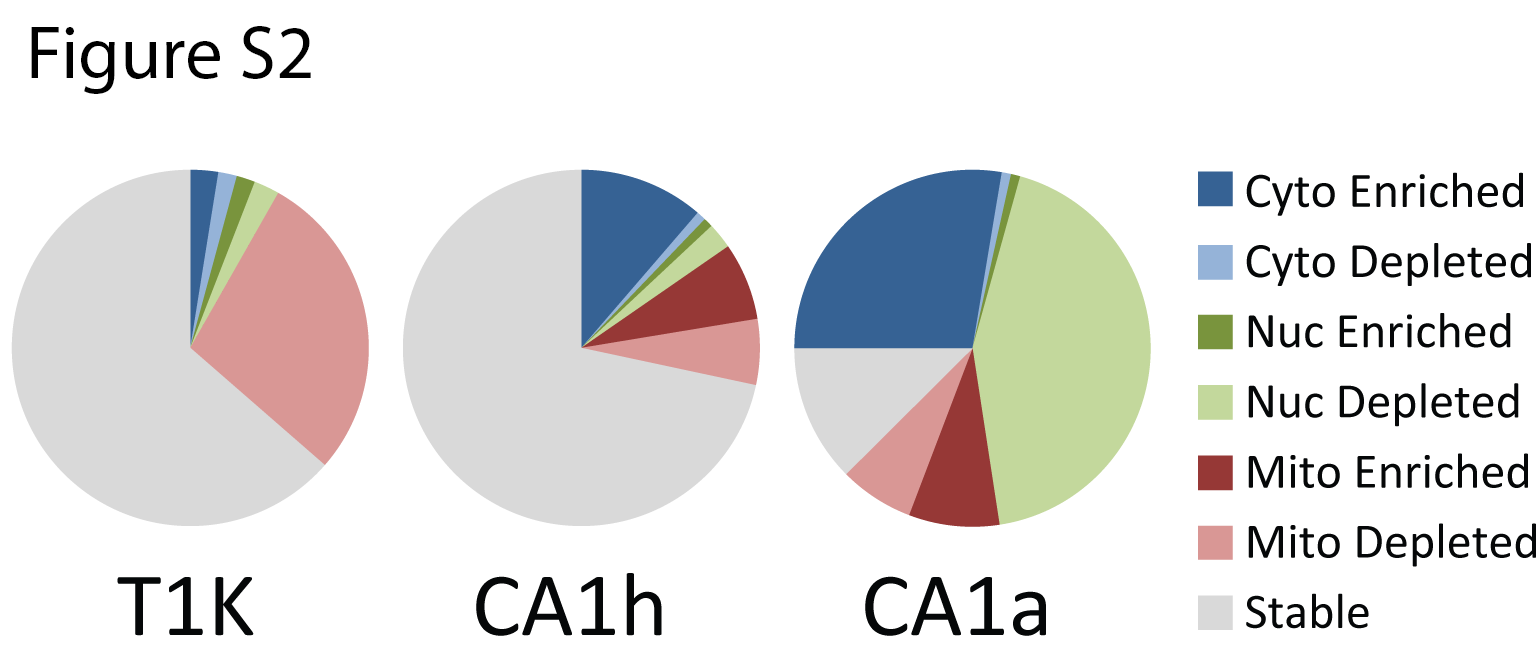

Supplement: Figure S2 — Quantitative delocalization of proteins relative to MCF10A parental cells. Relative enrichment of proteins was calculated by dividing fold change ratios within subcellular fractions by fold change ratios at the whole cell level to determine if changes in subcellular stoichiometries were indicative of protein delocalization (colors) or if the differences were consistent with changes in whole cell protein expression (gray color). Proteins were considered to be delocalized if there was greater than a 50% difference between the subcellular fraction ratio and the whole cell lysate ratio. (TIF) [file pone.0076220.s005.tif]
